# Assessment of Corticosteroid Therapy and Death or Disability According to Pretreatment Risk of Death or Bronchopulmonary Dysplasia in Extremely Preterm Infants

**DOI:** 10.1001/jamanetworkopen.2023.12277

**Published:** 2023-05-08

**Authors:** Erik A. Jensen, Laura Elizabeth Wiener, Matthew A. Rysavy, Kevin C. Dysart, Marie G. Gantz, Eric C. Eichenwald, Rachel G. Greenberg, Heidi M. Harmon, Matthew M. Laughon, Kristi L. Watterberg, Michele C. Walsh, Bradley A. Yoder, Scott A. Lorch, Sara B. DeMauro

**Affiliations:** 1 Division of Neonatology and Department of Pediatrics, Children’s Hospital of Philadelphia and University of Pennsylvania, Philadelphia; 2 Biostatistics and Epidemiology Division, RTI International, Research Triangle Park, North Carolina; 3 Department of Pediatrics, University of Texas McGovern Medical School, Houston; 4 Neonatal/Perinatal Medicine, Nemours Children’s Hospital, Wilmington, Delaware; 5 Department of Pediatrics and Duke Clinical Research Institute, Duke University School of Medicine, Durham, North Carolina; 6 Stead Family Department of Pediatrics, University of Iowa, Iowa City; 7 Department of Pediatrics, The University of North Carolina at Chapel Hill, Chapel Hill; 8 University of New Mexico Health Sciences Center, Albuquerque; 9 Eunice Kennedy Shriver National Institute of Child Health and Human Development, National Institutes of Health, Bethesda, Maryland; 10 Division of Neonatology, University of Utah, Salt Lake City

## Abstract

**Importance:**

Meta-analyses suggest that corticosteroids may be associated with increased survival without cerebral palsy in infants at high risk of bronchopulmonary dysplasia (BPD) but are associated with adverse neurologic outcomes in low-risk infants. Whether this association exists in contemporary practice is uncertain because most randomized clinical trials administered corticosteroids earlier and at higher doses than currently recommended.

**Objective:**

To evaluate whether the pretreatment risk of death or grade 2 or 3 BPD at 36 weeks’ postmenstrual age modified the association between postnatal corticosteroid therapy and death or disability at 2 years’ corrected age in extremely preterm infants.

**Design, Setting, and Participants:**

This cohort study analyzed data on 482 matched pairs of infants from 45 participating US hospitals in the National Institute of Child Health and Human Development Neonatal Research Network Generic Database (GDB). Infants were included in the cohort if they were born at less than 27 weeks’ gestation between April 1, 2011, and March 31, 2017; survived the first 7 postnatal days; and had 2-year death or developmental follow-up data collected between January 2013 and December 2019. Corticosteroid-treated infants were propensity score matched with untreated controls. Data were analyzed from September 1, 2019, to November 30, 2022.

**Exposure:**

Systemic corticosteroid therapy to prevent BPD that was initiated between day 8 and day 42 after birth.

**Main Outcomes and Measures:**

The primary outcome was death or moderate to severe neurodevelopmental impairment at 2 years’ corrected age. The secondary outcome was death or moderate to severe cerebral palsy at 2 years’ corrected age.

**Results:**

A total of 482 matched pairs of infants (mean [SD] gestational age, 24.1 [1.1] weeks]; 270 males [56.0%]) were included from 656 corticosteroid-treated infants and 2796 potential controls. Most treated infants (363 [75.3%]) received dexamethasone. The risk of death or disability associated with corticosteroid therapy was inversely associated with the estimated pretreatment probability of death or grade 2 or 3 BPD. The risk difference for death or neurodevelopmental impairment associated with corticosteroids decreased by 2.7% (95% CI, 1.9%-3.5%) for each 10% increase in the pretreatment risk of death or grade 2 or 3 BPD. This risk transitioned from estimated net harm to benefit when the pretreatment risk of death or grade 2 or 3 BPD exceeded 53% (95% CI, 44%-61%). For death or cerebral palsy, the risk difference decreased by 3.6% (95% CI, 2.9%-4.4%) for each 10% increase in the risk of death or grade 2 or 3 BPD and transitioned from estimated net harm to benefit at a pretreatment risk of 40% (95% CI, 33%-46%).

**Conclusions and Relevance:**

Results of this study suggested that corticosteroids were associated with a reduced risk of death or disability in infants at moderate to high pretreatment risk of death or grade 2 or 3 BPD but with possible harm in infants at lower risk.

## Key Points

**Question:**

Does the risk of death or neurodevelopmental disability associated with systemic corticosteroid therapy to prevent bronchopulmonary dysplasia (BPD) vary according to the estimated pretreatment probability of death or grade 2 or 3 BPD in extremely preterm infants?

**Findings:**

In this cohort study of 482 matched pairs of infants, corticosteroid therapy was associated with a possible increased risk of death or neurodevelopmental impairment at 2 years’ corrected age in infants with an estimated pretreatment probability of death or grade 2 or 3 BPD of less than 44% but a possible decreased risk of death or neurodevelopmental impairment in infants with a pretreatment probability greater than 61%. Similar associations were observed for death or cerebral palsy at pretreatment probabilities of less than 33% and greater than 46%.

**Meaning:**

Findings of this study suggest that postnatal corticosteroid therapy for the prevention of BPD in extremely preterm infants should be restricted to those at moderate to high risk of death or grade 2 or 3 BPD to avoid possible neurologic injury in infants with lower risk.

## Teaser

This cohort study examines the association between postnatal corticosteroid therapy to prevent bronchopulmonary dysplasia (BPD) and risk of death or neurodevelopmental impairment and death or cerebral palsy in infants born at less than 27 weeks’ gestation.

## Introduction

The use of postnatal corticosteroids to prevent bronchopulmonary dysplasia (BPD) is among the most controversial topics in neonatal medicine.^1^ Randomized clinical trials conducted in the 1980s and 1990s showed that dexamethasone improved weaning from respiratory support and reduced the risk of BPD in preterm infants.^2,3,4^ These findings led to prevalent administration of prolonged, high-dose corticosteroid therapy in preterm newborns.^1,5^ Subsequently, publication of trial follow-up data showed an increased risk of adverse neurodevelopment, particularly cerebral palsy (CP), with high-dose dexamethasone.^6,7,8,9^ This result prompted widespread recommendations against the use of systemic corticosteroids to prevent or treat BPD.^10,11^ A series of systematic reviews then proposed a more balanced approach by weighing the risks of neurodevelopmental impairment (NDI) due to corticosteroids against risks of BPD, itself associated with developmental disability.^12,13,14^ Metaregression analyses of randomized clinical trials by Doyle et al^12,15^ suggested that corticosteroids were associated with decreased rates of death or CP among infants at high risk of developing BPD. These findings prompted several pediatric societies to issue revised recommendations that indicated clinicians may consider low-dose corticosteroid therapy for very preterm infants receiving mechanical ventilation after 1 to 2 weeks of age due to the increased risk of BPD in this population.^16,17,18^ However, most trials that informed these revised guidelines initiated corticosteroids during the first week after birth or prescribed higher cumulative doses than the current recommendations.^15,19,20^ As a result, these trial data may not accurately characterize the risks and benefits of postnatal corticosteroid treatment strategies used to prevent BPD in contemporary extremely preterm infants. To address this knowledge gap, we performed a propensity score–matched cohort study to evaluate whether the pretreatment risk of death or grade 2 or 3 BPD at 36 weeks’ postmenstrual age (PMA) modified the association between postnatal corticosteroid therapy and death or disability at 2 years’ corrected age in extremely preterm infants.^21^

## Methods

### Design and Population

In this retrospective propensity score–matched cohort study, we used prospective data from the National Institute of Child Health and Human Development Neonatal Research Network (NRN) Generic Database (GDB)^22^ and Follow-up Study. Eligible infants were born at less than 27 weeks’ gestation between April 1, 2011, and March 31, 2017; survived the first 7 postnatal days; and had 2-year death or developmental follow-up data collected between January 2013 and December 2019. Infants who were enrolled in the NRN Hydrocortisone for BPD trial,^23,24^ treated with systemic corticosteroids for BPD beginning prior to postnatal day 8 or after day 42, had severe congenital disease, or had missing data for key variables were excluded. The institutional review boards at each of the 45 US hospitals participating in the GDB and Follow-up Study approved data collection. Per individual hospital guidelines, data were collected under a waiver of consent or after informed consent was obtained from parents or legal guardians. The Children's Hospital of Philadelphia Institutional Review Board deemed the present study exempt from review because it used deidentified data. We followed the Strengthening the Reporting of Observational Studies in Epidemiology (STROBE) reporting guideline.^25^

### Exposure and Outcomes

We evaluated the exposure of systemic corticosteroid therapy for BPD prevention that was initiated between day 8 and day 42 after birth. This range was selected to exclude infants who received corticosteroids in the first postnatal week and infants who were treated at ages when serial respiratory support data were no longer recorded. The GDB included information on the corticosteroid type and date of initiation but not the dose, treatment duration, or presence of subsequent treatment courses.

The primary outcome of this study was the composite of death between corticosteroid initiation (or the equivalent PMA in the untreated controls) and year 2 follow-up or moderate to severe NDI, which was defined as a Bayley-III cognitive or motor composite score lower than 85, Gross Motor Function Classification System (GMFCS) level 2 or higher, moderate to severe CP, and/or severe visual or hearing impairment.^26,27^ The secondary outcome was the composite of death or moderate to severe CP, which was defined as GMFCS level 2 or higher and a clinical diagnosis of CP at year 2 follow-up.^27^ Post hoc sensitivity analyses were performed using composite outcomes of death or severe NDI and death or severe CP.^26,27^ Severe NDI was defined as a Bayley-III cognitive or motor composite score lower than 70, GMFCS level 4 or higher, severe CP, and/or severe visual or hearing impairment.^26,27^ Severe CP was defined as GMFCS level 4 or higher and a clinical diagnosis of CP.^27^

Grade 2 or 3 BPD was defined as treatment with mechanical ventilation, noninvasive positive airway pressure, or nasal cannula delivering greater than 2 L/min. Grade 2 or 3 BPD was assessed as a composite with death at 36 weeks’ PMA.^21^

### Pretreatment Risk and Matched Cohort

We estimated the pretreatment probability of death or grade 2 or 3 BPD at 36 weeks’ PMA for all eligible untreated controls using a logistic regression model that was fitted with 39 fixed and repeatedly measured variables (eAppendix in Supplement 1) that characterize respiratory state or are known or believed to be associated with the study outcomes.^28^ Parameter estimates from this model were used to calculate the pretreatment probability of death or grade 2 or 3 BPD in corticosteroid-treated infants, with data collected until corticosteroid initiation. Dates of treatment or diagnosis were used to determine the age at occurrence for many time-variable covariates. Respiratory support and oxygen therapy data were available at 24 hours, 3 days, 7 days, 14 days, and 28 days. To minimize bias and maximize the number of evaluated infants, we included infants who were missing values for multilevel demographic variables (maternal educational level and race and ethnicity) in the missing categories during model fitting. Maternal race and ethnicity reported in the GDB were self-identified and selected from the following options: American Indian or Alaska Native, Asian, Black, Hispanic or Latino, Native Hawaiian or other Pacific Islander, White, more than 1 race, unknown, or not reported. Maternal race and ethnicity were included in this study because they are known to be associated with differences in care and outcomes in preterm infants.^29^ The composite of death or grade 2 or 3 BPD at 36 weeks’ PMA was chosen to classify pretreatment risk to account for early posttreatment death as a competing outcome and to provide a broad evaluable range of risks.^30^

Propensity scores that were based on the probability that infants would receive corticosteroids to prevent BPD were used to match treated infants to untreated controls. Propensity scores were calculated with logistic regression models that were fitted with the same explanatory variables as those used for estimating the probability of death or grade 2 or 3 BPD.^28^ For each eligible untreated control, separate propensity scores were generated for each completed PMA week the infant was alive between 23 weeks’ and 32 weeks’ PMA. For each infant who received corticosteroids, a single propensity score corresponding to the PMA week at treatment initiation was computed. Untreated infants were matched without replacement to treated infants using the 1:1 greedy nearest neighbor method, with a maximum caliper of 0.25 times the pooled estimate of the common SD of the propensity score logits^31^ and exact matching on the following variables: gestational age, PMA week at corticosteroid initiation (or corresponding PMA in controls), last known mode of respiratory support prior to corticosteroid therapy and corresponding age at data collection, and decile of the estimated probability of death or grade 2 or 3 BPD.

Infant characteristics were summarized using means (SDs) for continuous data and rates (proportions) for categorical data. Standardized differences, expressed as absolute values, were used to compare baseline characteristics between corticosteroid-treated infants and untreated controls. Standardized difference values less than 0.1 indicated negligible between-group differences.

### Statistical Analysis

The primary analysis used logistic regression to estimate the probability of death or moderate to severe NDI for each matched infant based on corticosteroid treatment status and the same covariates for estimating the probability of death or grade 2 or 3 BPD. Risk difference values for each matched pair were calculated by subtracting the model-estimated probability of death or NDI in the untreated infants from the matched corticosteroid-treated infants. Risk differences were then regressed on the pretreatment probability of death or grade 2 or 3 BPD in each untreated matched control using linear regression. The slope and corresponding 95% CI for the regression line indicated whether the treatment effect of corticosteroids varied according to the probability of death or grade 2 or 3 BPD. Equivalent analyses were performed for the composite outcomes of death or moderate to severe CP, death or severe NDI, and death or severe CP.

Secondary analyses evaluated the association between corticosteroids and the study outcomes, which were stratified by the pretreatment probability of death or grade 2 or 3 BPD classified as a 5-level categorical variable. The number of strata was selected by investigator consensus; strata cut points were chosen to produce similarly sized groups with clinically interpretable ranges. Stratum-specific odds ratios (ORs) were computed using a logistic regression model that was fitted with corticosteroid treatment status, the 5-level probability of death or grade 2 or 3 BPD, treatment-by-stratum interaction term, study center, and PMA week at corticosteroid initiation. A Wald test on the interaction term was performed to quantify whether the treatment effect of corticosteroids differed across strata.

Finally, we conducted exploratory analyses to assess for drug-specific treatment effects for the prespecified outcomes of death or moderate to severe NDI and death or moderate to severe CP. Terms for corticosteroid type, probability of death or grade 2 or 3 BPD, and their interaction were added to the linear regression models used in the primary analyses. Separate logistic regression models for dexamethasone and hydrocortisone computed drug-specific ORs that were stratified by pretreatment risk subgroups.

All analyses were conducted using SAS, version 9.4 (SAS Institute Inc). Statistical inferences were based on 2-tailed tests, with significance set at 2-sided *P* < .05. Additional details of the statistical approach are provided in the eAppendix in Supplement 1. Data were analyzed from September 1, 2019, to November 30, 2022.

## Results

**Figure 1.**
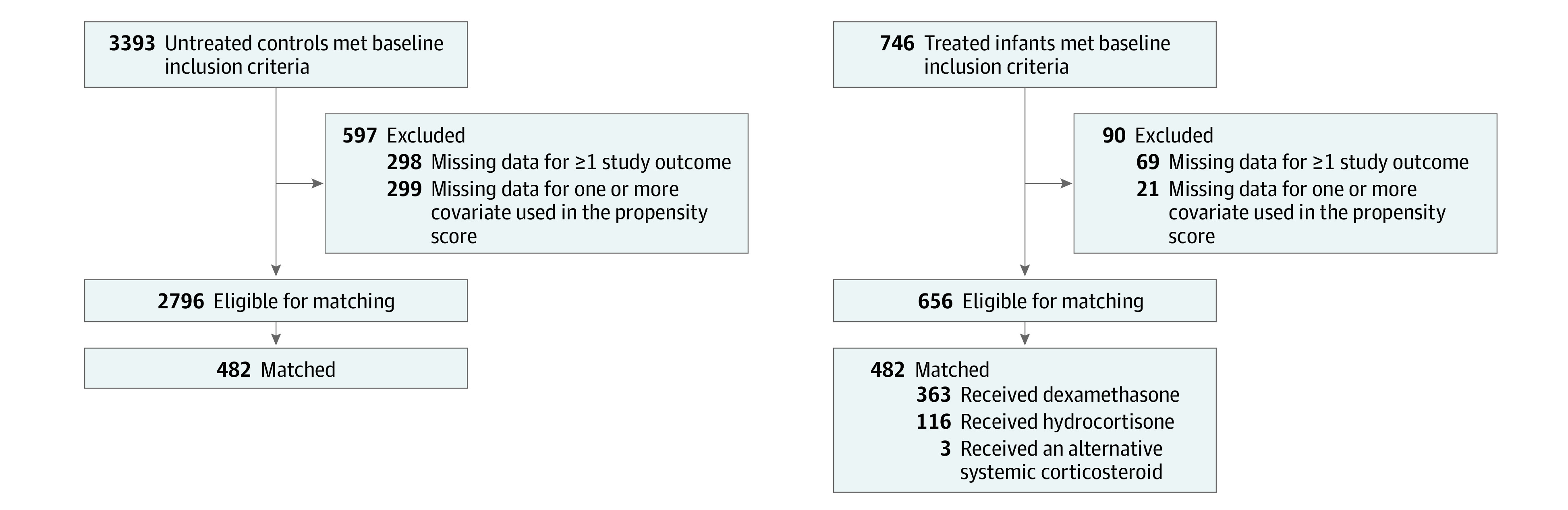
Study Flow Diagram

The cohort included 482 matched pairs of infants (mean [SD] gestational age, 24.1 [1.1] weeks]; 212 females [44%] and 270 males [56.0%]) from 656 eligible corticosteroid-treated infants and 2796 potential controls (Figure 1). Most demographic and pretreatment clinical characteristics were similar between eligible infants and those who were excluded due to missing data (eTable 1 and eTable 2 in Supplement 1) and between eligible matched and unmatched infants who received corticosteroids (eTable 3 in Supplement 1). The few observed differences suggested modestly higher mean disease acuity in the matched infants compared with excluded or unmatched infants. All evaluated characteristics were well balanced in the matched cohort (Table 1; eAppendix, eTables 4 and 5, and eFigure 1 in Supplement 1). The estimated mean (SD) pretreatment probability of death or grade 2 or 3 BPD at 36 weeks’ PMA was 0.53% (0.22%) in both the matched treated and untreated infants. The mean (SD) age at corticosteroid initiation was 25.2 (8.5) days (distribution is shown in eFigure 2 in Supplement 1). Of the treated infants, 363 (75.3%) received dexamethasone, 116 (24.1%) received hydrocortisone, and 3 (0.6%) received alternative corticosteroids.

**Table 1.** Characteristics of Eligible and Matched Infants by Corticosteroid Treatment Status^a^

Characteristic	Eligible sample			Matched sample		
	Corticosteroid-treated infants, No./total No. (%)	Untreated controls, No./total No. (%)	Standardized difference	Corticosteroid- treated infants, No./total No. (%)	Untreated controls, No./total No. (%)	Standardized difference
**Maternal**						
Antenatal corticosteroids	597/656 (91.0)	2507/2796 (89.7)	0.05	435/482 (90.2)	427/482 (88.6)	0.05
Antenatal antibiotics	497/656 (75.8)	2095/2796 (74.9)	0.02	357/482 (74.1)	345/482 (71.6)	0.06
Multiple birth	179/656 (27.3)	695/2796 (24.9)	0.06	127/482 (26.3)	127/482 (26.3)	0.00
Race and ethnicity^b^						
Black race	277/636 (43.6)	1243/2719 (45.7)	0.04	198/469 (42.2)	201/473 (42.5)	0.01
White race	327/636 (54.4)	1320/2719 (48.5)	0.04	242/469 (51.6)	247/473 (52.2)	0.01
Other race	32/636 (5.0)	156/2719 (5.7)	0.04	29/469 (6.2)	25/473 (5.3)	0.01
Hispanic or Latino ethnicity	67/650 (10.3)	419/2765 (15.2)	0.15	53/478 (11.1)	54/479 (11.3)	0.01
Non-Hispanic or non-Latino ethnicity	583/650 (89.7)	2346/2719 (86.3)	0.15	425/478 (88.9)	425/479 (88.7)	0.01
Greater than high school educational level	283/520 (54.4)	1131/2261 (50.0)	0.09	207/384 (53.9)	223/382 (58.4)	0.09
**Demographic and perinatal**						
Gestational age, mean (SD), wk	24.3 (1.1)	24.9 (1.1)	0.57	24.4 (1.1)	24.4 (1.1)	0.00
Birth weight, mean (SD), g	673 (135)	763 (166)	0.59	682 (135)	672 (146)	0.08
Female sex	282/656 (43.0)	1434/2796 (51.3)	0.17	212/482 (44.0)	212/482 (44.0)	0.00
Male sex	374/656 (57.0)	1362/2796 (48.7)	0.17	270/482 (56.0)	270/482 (56.0)	0.00
Endotracheal tube placed in the delivery room	573/656 (87.3)	2133/2796 (76.3)	0.29	415/482 (86.1)	426/482 (88.4)	0.07
Received surfactant	634/656 (96.6)	2471/2796 (88.4)	0.32	463/482 (96.1)	469/482 (97.3)	0.07
Received indomethacin in the first 24 h	243/656 (37.0)	1179/2796 (42.2)	0.10	182/482 (37.8)	187/482 (38.8)	0.02
Treated for hypotension in the first 24 h	241/656 (36.7)	825/2796 (29.5)	0.15	191/482 (39.6)	180/482 (37.3)	0.05
**Postnatal^c^**						
Grade 3 or 4 IVH	NA	NA	NA	68/482 (14.1)	69/428 (14.3)	0.01
Culture-confirmed sepsis or meningitis	NA	NA	NA	102/482 (21.2)	92/482 (19.1)	0.05
Surgery for necrotizing enterocolitis or intestinal perforation	NA	NA	NA	35/482 (7.3)	41/482 (8.5)	0.05
Days on positive airway pressure	NA	NA	NA	20.4 (7.7)	20.4 (7.6)	0.002
Days on invasive ventilation	NA	NA	NA	18.6 (7.8)	18.5 (8.0)	0.01
Last recorded mode of support						0.00
HFV	NA	NA	NA	213/482 (44.2)	213/482 (44.2)	
Conventional ventilation	NA	NA	NA	240/482 (49.8)	240/482 (49.8)	
NIMV, nCPAP, or NC	NA	NA	NA	29/482 (6.0)	29/482 (6.0)	
Last recorded Fio_2_, mean (SD)	NA	NA	NA	0.57 (0.24)	0.56 (0.24)	0.02
Pretreatment probability of death or grade 2 or 3 BPD at 36 weeks’ PMA, mean (SD)	NA	NA	NA	0.53 (0.22)	0.53 (0.22)	0.01

Abbreviations: BPD, bronchopulmonary dysplasia; Fio_2_, fraction of inspired oxygen; HFV, high-frequency ventilation; IVH, intraventricular hemorrhage; NA, not applicable; NC, nasal cannula; nCPAP, nasal continuous positive airway pressure; NIMV, noninvasive mechanical ventilation; PMA, postmenstrual age.

^a^ Data are expressed as the number (%) of infants unless otherwise indicated. Data are shown for the infants with known values for the specified covariates. A full list of all variables used to estimate the propensity score values is provided in the eAppendix in Supplement 1. Comparisons of all propensity score variables between corticosteroid-treated and untreated controls who were eligible or included in the matched cohort are provided in eTables 4 and 5 in Supplement 1. The distribution of propensity score values in the matched cohort is shown in eFigure 1 in Supplement 1.

^b^ Race and ethnicity were self-identified in the Generic Database and selected from the following options: Black, Hispanic or Latino, or White. The “other” catgory included American Indian or Alaska Native, Asian, Native Hawaiian or other Pacific Islander, more than 1 race, unknown, or not reported.

^c^ Values indicate postnatal exposures recorded prior to corticosteroid initiation or the same PMA week in the matched controls. Similar values could not be computed for the eligible untreated controls prior to matching on a specific PMA.

Table 2 reports the study outcomes in the full matched cohort. There were no differences in the adjusted odds of death or moderate to severe NDI (adjusted OR [aOR], 0.92; 95% CI, 0.68-1.24) or death or moderate to severe CP (aOR, 0.83; 95% CI, 0.60-1.15) at 2 years’ corrected age associated with corticosteroid therapy. The adjusted odds of death or grade 2 or 3 BPD at 36 weeks’ PMA were not significantly different between the groups (aOR, 1.35; 95% CI, 0.98-1.86). The rates and timing of mortality are shown in eTable 6 in Supplement 1.

**Table 2.** Outcomes in Matched Infants Associated With Corticosteroid Treatment Status^a^

Outcome	Observed rates		Adjusted OR (95% CI)^b^
	Corticosteroid-treated infants, No./total No. (%)	Untreated controls, No./total No. (%)	
Death or moderate to severe NDI at 2 years’ corrected age	323/470 (68.7)	332/482 (68.9)	0.92 (0.68-1.24)
Death prior to 2 years’ corrected age	93/482 (19.3)	118/482 (24.5)	0.86 (0.59-1.23)
Moderate to severe NDI among survivors	230/377 (61.0)	214/364 (58.8)	1.00 (0.72-1.38)
Death or moderate to severe cerebral palsy at 2 years’ corrected age	130/482 (27.0)	154/482 (32.0)	0.83 (0.60-1.15)
Moderate to severe cerebral palsy among survivors	37/389 (9.5)	36/364 (9.9)	0.77 (0.44-1.35)
Death or grade 2 or 3 BPD at 36 weeks’ PMA	297/478 (62.1)	259/482 (53.7)	1.35 (0.98-1.86)

Abbreviations: NDI, neurodevelopmental impairment; OR, odds ratio; PMA, postmenstrual age.

^a^ Values shown in the table exclude infants for whom developmental outcome data at 2 years’ corrected age were not available.

^b^ Models were adjusted for estimated pretreatment probability of death or grade 2 or 3 bronchopulmonary dysplasia at 36 weeks’ PMA (classified as a 5-level categorical variable), corticosteroid treatment by probability of death or grade 2 or 3 bronchopulmonary dysplasia stratum interaction term, PMA week at corticosteroid treatment or the corresponding PMA in the untreated matched controls, and study center.

**Figure 2.**
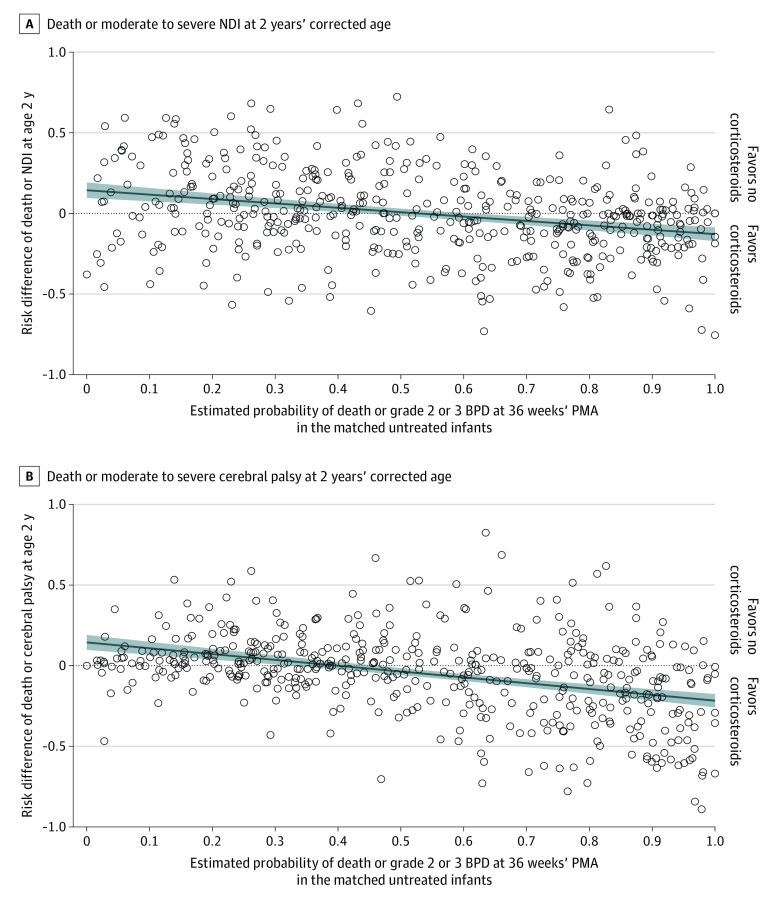
Risk Differences for Death or Disability vs the Pretreatment Probability of Death or Grade 2 or 3 Bronchopulmonary Dysplasia (BPD) at 36 Weeks’ Postmenstrual Age (PMA) Circles represent values for individual matched pairs; shaded areas represent regression lines and 95% CIs. For death or neurodevelopmental impairment (NDI), the regression line equation is y = 0.144 – 0.272x and crosses the x-axis at an estimated probability of death or grade 2 or 3 BPD of 53% (95% CI, 44%-61%). For death or cerebral palsy (CP), the regression line equation is y = 0.144 − 0.361x and crosses the x-axis at 40% (95% CI, 33%-46%). Logistic regression model c-statistic values in the matched cohort are 0.753 for death or grade 2 or 3 BPD at 36 weeks’ PMA, 0.766 for death or NDI at 2 years, and 0.812 for death or CP at 2 years.

There was an inverse association between the pretreatment probability of death or grade 2 or 3 BPD at 36 weeks’ PMA and the risk differences for death or disability associated with corticosteroid therapy (Figure 2). For each 10% increase in the probability of death or grade 2 or 3 BPD, the risk difference for death or moderate to severe NDI associated with corticosteroids decreased by 2.7% (95% CI, 1.9%-3.5%). The fitted regression line crossed the x-axis at a probability of death or grade 2 or 3 BPD of 53% (95% CI, 44%-61%); the CIs for this regression analysis represented the point of intersection between the 95% confidence bands of the fitted regression line and the x-axis. These values quantified the pretreatment probability of death or grade 2 or 3 BPD where the risk of death or moderate to severe NDI associated with corticosteroids transitioned from estimated net harm to benefit. The corresponding analysis for death or moderate to severe CP also found an inverse association. For each 10% increase in the pretreatment probability of death or grade 2 or 3 BPD, the risk difference for death or CP associated with corticosteroid therapy decreased by 3.6% (95% CI, 2.9%-4.4%). The fitted regression line crossed the x-axis at a probability of death or grade 2 or 3 BPD of 40% (95% CI, 33%-46%). The post hoc sensitivity analyses demonstrated similar inverse associations between the probability of death or grade 2 or 3 BPD and the risks of death or severe disability associated with corticosteroids (eFigure 3 in Supplement 1).

eFigures 4 and 5 in Supplement 1 show the results of the secondary analyses that classified the pretreatment probability of death or grade 2 or 3 BPD at 36 weeks’ PMA as a 5-level categorical variable. Consistent with the results of the primary analyses, the OR point estimates for the death or disability outcomes decreased from values greater than 1 (favoring no treatment) to values less than 1 (favoring corticosteroids) as the pretreatment probability of death or grade 2 or 3 BPD increased. There was evidence of significant effect modification by the pretreatment probability of death or grade 2 or 3 BPD for the outcomes of death or moderate to severe CP (treatment × probability-strata interaction *P* = .03) and death or severe NDI (treatment × probability-strata interaction *P* = .004).

**Figure 3.**
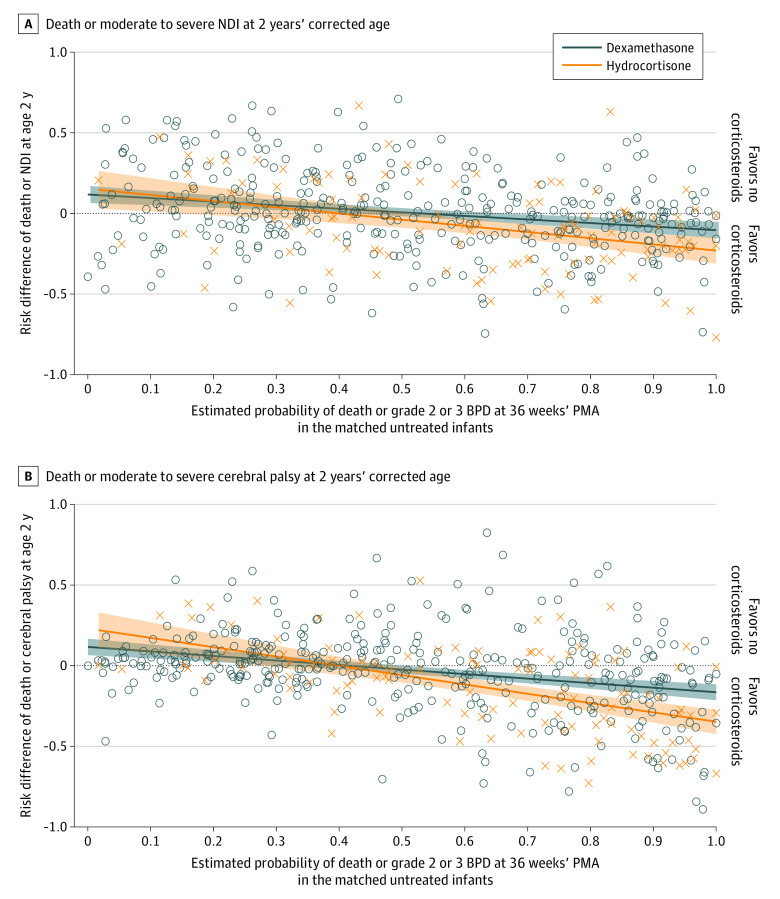
Risk Differences for Death or Disability Stratified by Treatment With Dexamethasone or Hydrocortisone Circles and Xs represent values computed for individual matched pairs with dexamethasone and hydrocortisone treatment, respectively; shaded areas represent regression lines and 95% CIs. The regression line for death or neurodevelopmental impairment (NDI) is y = 0.131 – 0.221x for dexamethasone and y = 0.167 – 0.384x for hydrocortisone. For dexamethasone, the regression line crosses the x-axis at a pretreatment probability of death or grade 2 or 3 bronchopulmonary dysplasia (BPD) of 59% (95% CI, 48%-74%) for dexamethasone and 44% (95% CI, 21%-56%) for hydrocortisone. The regression line for death or cerebral palsy crosses the x-axis at 42% (95% CI, 31%-50%) for dexamethasone and 40% (95% CI, 28%-48%) for hydrocortisone. PMA indicates postmenstrual age.

There was no evidence of a drug-specific treatment effect for the primary outcome of death or moderate to severe NDI (drug × pretreatment probability interaction *P* = .11) (Figure 3). There was a possible treatment advantage associated with dexamethasone for death or moderate to severe CP (interaction *P* = .002) (Figure 3). In the 5-level categorical analysis, dexamethasone compared with no corticosteroid therapy was associated with lower adjusted odds of death or moderate to severe CP among infants in the strata with estimated pretreatment probabilities of death or grade 2 or 3 BPD that were greater than 65% (eFigure 6 in Supplement 1).

## Discussion

This propensity score–matched cohort study examined the association between systemic corticosteroid therapy initiated after the first postnatal week for BPD prevention and death or disability at 2 years’ corrected age in a contemporary, multicenter cohort of extremely preterm infants. When averaged over the full matched cohort, postnatal corticosteroids were not associated with a significant increase or decrease in the risk of death or moderate to severe NDI or death or moderate to severe CP. However, the pretreatment probability of death or grade 2 or 3 BPD at 36 weeks’ PMA modified the association between corticosteroid therapy and the risks of death or disability. Corticosteroids were associated with a reduced risk of death or disability among infants at moderate to high risk of death or grade 2 or 3 BPD but also were associated with a possible increased risk of death or disability among infants at low risk of death or grade 2 or 3 BPD.

Metaregressions by Doyle et al^12,15^ suggested that the risk of BPD, defined as oxygen therapy at 36 weeks’ PMA, modified the association between corticosteroid therapy and death or CP. These previous studies differed methodologically from the present study. Doyle et al^12,15^ used summarized trial results and defined baseline risks with the observed rates of BPD in the trial control groups. In contrast, we used participant-level observational data and stratified infants based on the estimated pretreatment risk of death or grade 2 or 3 BPD. Moreover, current guidelines recommend the administration of corticosteroids at lower treatment doses and later chronological ages than were studied in some of the trials evaluated by Doyle et al.^15,16,17,18^ Despite these differences, we identified similar baseline risks for BPD at which the risk-to-benefit balance favored corticosteroid use for the prevention of death or CP. Doyle et al^15^ reported a treatment benefit when BPD risk exceeded 46% (95% CI, 33%-60%). We estimated that a potential benefit exceeded harm at a pretreatment probability of death or grade 2 or 3 BPD greater than 40% (95% CI, 33%-46%). Collectively, these studies provide further evidence that postnatal corticosteroid therapy may be associated with reduced risk of adverse neurologic sequelae in extremely preterm infants at moderate to high risk of BPD but also may be associated with harm to neurodevelopment in extremely preterm infants at low risk of BPD.

We did not identify a significant difference in the odds of death or grade 2 or 3 BPD associated with corticosteroids in the full matched cohort. This result should be interpreted cautiously. The study was not designed to evaluate death or grade 2 or 3 BPD as a primary treatment outcome and should not supplant meta-analyses of randomized clinical trials, which show reduced risk of death or BPD associated with corticosteroids initiated after the first postnatal week.^32^ We acknowledge that unmeasured differences in disease acuity between matched pairs may contribute to the modestly higher rates of death or grade 2 or 3 BPD observed in corticosteroid-treated infants. However, the finding of a possible small decrease, rather than increase, in the mean odds of death or disability in the full study cohort adds credibility to the results and suggests that residual confounding, if present, may mask a more favorable risk-to-benefit profile for corticosteroids.

Meta-analyses suggest that dexamethasone but not hydrocortisone may be associated with increased risk of adverse neurodevelopmental outcomes.^16,23,32,33^ We did not find evidence of a differential treatment effect associated with dexamethasone vs hydrocortisone for death or moderate to severe NDI at 2 years’ corrected age. However, there was a possible drug-specific treatment effect for death or moderate to severe CP. When stratifying the cohort into subgroups according to the pretreatment probability of death or grade 2 or 3 BPD, dexamethasone was associated with a significant reduction in the risk of death or CP among infants with risks of death or grade 2 or 3 BPD greater than 65%. There were no significant differences in the odds of death or CP associated with hydrocortisone. These exploratory analyses support the investigation of low-dose dexamethasone as a possible medication to reduce respiratory morbidity and improve neurologic outcomes in infants at high risk for BPD. These results should otherwise be interpreted with caution owing to the smaller number of infants in each subgroup and the multiplicity of statistical comparisons.

### Strengths and Limitations

This study has several strengths. First, to our knowledge, the study is among the largest multicenter analyses to examine long-term neurologic outcomes associated with corticosteroid therapy initiated after the first postnatal week in extremely preterm infants. Second, unlike in most randomized clinical trials of postnatal corticosteroids wherein frequent open-label corticosteroid use may bias study findings, we restricted matched controls to those who did not receive corticosteroids for the prevention of BPD.^34^ Some controls may have received corticosteroids for other indications, such as adrenal insufficiency or hypotension. Third, quantification of pretreatment risk using a composite of death or BPD at 36 weeks’ PMA, rather than BPD alone, may improve selection of patients for corticosteroid therapy owing to the increased probability of early death among infants at higher risk of BPD. We internally calculated the risk of death or grade 2 or 3 BPD using a large number of covariates in an effort to optimize pretreatment risk estimation and propensity score matching.^28^ More parsimonious models for estimating BPD risk in clinical practice are available online.^35,36^

The use of observational data is the primary study limitation. We used propensity score matching to improve causal inference but acknowledge the possibility of residual confounding by unmeasured differences among matched infants. Data for some covariates were only available for discrete time points and may not fully reflect the infants’ status immediately prior to corticosteroid therapy. Furthermore, we excluded a small number of corticosteroid-treated infants who could not be matched to a suitable control and those who were missing data for key study covariates. It is possible that alternative approaches to address missing data, including imputation, may produce modestly different study results. Additionally, we did not have information on the dose or duration of corticosteroid therapy. A recent analysis of the multicenter Prematurity and Respiratory Outcomes Program reported that extremely preterm infants who were treated with dexamethasone received a median daily dose of 0.2 mg/kg and a median cumulative dose of 1.2 mg/kg.^37^ Similar exposures may have occurred in the present study. More than one-half of the hospitals that participated in the Prematurity and Respiratory Outcomes Program contributed data to the present analysis as members of the NRN.^37^

## Conclusions

In this propensity score–matched cohort study, the pretreatment probability of death or grade 2 or 3 BPD at 36 weeks’ PMA modified the association between postnatal systemic corticosteroid therapy to prevent BPD and the risk of death or disability at 2 years’ corrected age. These findings were consistent with prior meta-analyses of randomized clinical trials and provide important evidence that contemporary dosing strategies for systemic corticosteroid therapy may be associated with decreased risk of adverse neurodevelopmental outcomes when restricted to preterm infants at moderate to high risk of death or BPD.^12,15^ The possible treatment advantage with dexamethasone found in the present study supports further unbiased evaluation of this medication to prevent BPD and improve neurodevelopment.

## Notes

Supplement 1. **eAppendix.** Additional Information on the Statistical Analyses **eTable 1.** Characteristics of Corticosteroid-Treated Infants Who Were Excluded Owing to Missing Data and Those Eligible for Matching **eTable 2.** Characteristics of Untreated Infants Who Were Excluded Owing to Missing Data and Those Eligible for Matching **eTable 3.** Characteristics of the Matched Corticosteroid-Treated Infants and Eligible But Unmatched Corticosteroid-Treated Infants (Excludes Infants With Missing Data) **eTable 4.** Characteristics of Corticosteroid-Treated Infants and Untreated Controls Eligible for Matching (Excludes Infants With Missing Data) **eTable 5.** Characteristics of Corticosteroid-Treated Infants and Untreated Controls in the Final Matched Cohort **eFigure 1.** Distribution of Propensity Score Values in the Matched Cohort **eFigure 2.** Distribution of the Timing of Corticosteroid Initiation **eTable 6.** Rates and Timing of Mortality in the Matched Cohort **eFigure 3.** Risk Differences for Death or Severe Disability Versus the Probability of Death or Grade 2-3 BPD **eFigure 4.** Odds Ratios for Death or Moderate to Severe Disability Stratified by the Pre-Treatment Probability of Death or Grade 2-3 BPD as a 5-Level Categorical Variable **eFigure 5.** Odds Ratios for Death or Severe Disability Stratified by the Pre-Treatment Probability of Death or Grade 2-3 BPD as a 5-Level Categorical Variable **eFigure 6.** Odds Ratios for Death or Moderate to Severe Disability Stratified by Treatment With Dexamethasone vs. Hydrocortisone

Supplement 2.
Nonauthor Contributors

Supplement 3.
Data Sharing Statement
