# Supplementary material for: Assessment of Corticosteroid Therapy and Death or Disability According to Pretreatment Risk of Death or Bronchopulmonary Dysplasia in Extremely Preterm Infants
Source: JAMA Netw Open. 2023 May 8;6(5):e2312277. doi: 10.1001/jamanetworkopen.2023.12277 (PMC10167571; doi:10.1001/jamanetworkopen.2023.12277)
Supplement: Supplement 1. — eAppendix. Additional Information on the Statistical Analyses eTable 1. Characteristics of Corticosteroid-Treated Infants Who Were Excluded Owing to Missing Data and Those Eligible for Matching eTable 2. Characteristics of Untreated Infants Who Were Excluded Owing to Missing Data and Those Eligible for Matching eTable 3. Characteristics of the Matched Corticosteroid-Treated Infants and Eligible But Unmatched Corticosteroid-Treated Infants (Excludes Infants With Missing Data) eTable 4. Characteristics of Corticosteroid-Treated Infants and Untreated Controls Eligible for Matching (Excludes Infants With Missing Data) eTable 5. Characteristics of Corticosteroid-Treated Infants and Untreated Controls in the Final Matched Cohort eFigure 1. Distribution of Propensity Score Values in the Matched Cohort eFigure 2. Distribution of the Timing of Corticosteroid Initiation eTable 6. Rates and Timing of Mortality in the Matched Cohort eFigure 3. Risk Differences for Death or Severe Disability Versus the Probability of Death or Grade 2-3 BPD eFigure 4. Odds Ratios for Death or Moderate to Severe Disability Stratified by the Pre-Treatment Probability of Death or Grade 2-3 BPD as a 5-Level Categorical Variable eFigure 5. Odds Ratios for Death or Severe Disability Stratified by the Pre-Treatment Probability of Death or Grade 2-3 BPD as a 5-Level Categorical Variable eFigure 6. Odds Ratios for Death or Moderate to Severe Disability Stratified by Treatment With Dexamethasone vs. Hydrocortisone [file jamanetwopen-e2312277-s001.pdf]

Supplemental Online Content

Jensen EA, Wiener LE, Rysavy MA, et al; Eunice Kennedy Shriver National Institute of Child Health and Human Development Neonatal Research Network. Assessment of corticosteroid therapy and death or disability according to pretreatment risk of death or bronchopulmonary dysplasia in extremely preterm infants. *JAMA Netw Open*. 2023;6(5):e2312277. doi:10.1001/jamanetworkopen.2023.12277

eAppendix. Additional Information on the Statistical Analyses

eTable 1. Characteristics of Corticosteroid-Treated Infants Who Were Excluded Owing to Missing Data and Those Eligible for Matching

eTable 2. Characteristics of Untreated Infants Who Were Excluded Owing to Missing Data and Those Eligible for Matching

eTable 3. Characteristics of the Matched Corticosteroid-Treated Infants and Eligible But Unmatched Corticosteroid-Treated Infants (Excludes Infants With Missing Data)

eTable 4. Characteristics of Corticosteroid-Treated Infants and Untreated Controls Eligible for Matching (Excludes Infants With Missing Data)

eTable 5. Characteristics of Corticosteroid-Treated Infants and Untreated Controls in the Final Matched Cohort

eFigure 1. Distribution of Propensity Score Values in the Matched Cohort

eFigure 2. Distribution of the Timing of Corticosteroid Initiation

eTable 6. Rates and Timing of Mortality in the Matched Cohort

eFigure 3. Risk Differences for Death or Severe Disability Versus the Probability of Death or Grade 2-3 BPD

eFigure 4. Odds Ratios for Death or Moderate-to-Severe Disability Stratified by the Pre-Treatment Probability of Death or Grade 2-3 BPD as a 5-Level Categorical Variable

eFigure 5. Odds Ratios for Death or Severe Disability Stratified by the Pre-Treatment Probability of Death or Grade 2-3 BPD as a 5-Level Categorical Variable

© 2023 Jensen EA et al. *JAMA Network Open.*

eFigure 6. Odds Ratios for Death or Moderate to Severe Disability Stratified by Treatment With Dexamethasone vs. Hydrocortisone

This supplemental material has been provided by the authors to give readers additional information about their work.

© 2023 Jensen EA et al. *JAMA Network Open.*

eAppendix. Additional information on the statistical analyses

Prediction of the pre-treatment probability of death or grade 2-3 bronchopulmonary dysplasia (BPD) by 36 weeks' postmenstrual age (PMA) in the eligible cohort:
The pre-treatment probability of death or grade 2-3 BPD at 36 weeks' PMA was predicted using data recorded on all eligible untreated infants at all possible match PMAs using logistic regression. The model parameter estimates were then used to predict pre-treatment probabilities for all eligible infants who received corticosteroid therapy. The covariates used in the model are listed below. All variables were included in regression models as a dichotomous or categorical variable unless otherwise indicated. For continuous variables, the assumption of linear relationships between the independent variable and log odds of the primary outcome was assessed via graphical inspection and by testing of Box-Tidwell transformations.

- Antenatal steroids
- Antenatal antibiotics
- Private insurance
- Outborn status
- Birthweight (continuous variable, centered)
- Gestational age (completed weeks as a continuous variable)
- Sex
- Small for gestational age
- Surfactant
- Multiple birth
- Magnesium sulfate
- C-section
- Indomethacin
- Maternal hypertension
- Chorioamnionitis
- Intubation in the delivery room
- Chest compressions/epinephrine in the delivery room
- Enteral feeds initiated within 7 days of age
- Birth year (continuous variable, centered)
- Mother's highest education (< high school, high school graduate, > high school, missing)
- Mother's self-reported race (Black, White, other, missing)
- Hispanic origin of mother by self-report (yes, no, missing)
- Highest mode of respiratory support at 24 hours of age (high frequency ventilation, conventional ventilation, non-invasive intermittent pressure ventilation, nasal continuous positive airway pressure, nasal cannula/oxygen hood/no support)
- FiO_2 at 24 hours of age (categorical: 21%, 22%-30%, 31%-40%, 41%-50%, 51%-99%, 100%)
- Intraventricular hemorrhage (IVH) prior to corticosteroid initiation (or the equivalent PMA week for the untreated infant)
- Sepsis or meningitis prior to corticosteroid initiation (or the equivalent PMA week for the untreated infant)
- Surgical necrotizing enterocolitis (NEC) or spontaneous gastrointestinal perforation (SIP) prior to corticosteroid initiation (or the equivalent PMA week for the untreated infant)
- Nitric oxide prior to corticosteroid initiation (or the equivalent PMA week for the untreated infant)
- Days on invasive mechanical ventilation (IMV) prior to corticosteroid initiation (or the equivalent PMA week for the untreated infant) (continuous variable)
- Days on positive airway pressure (PAP) prior to corticosteroid initiation (or the equivalent PMA week for the untreated infant) (continuous variable)
- Last recorded FiO_2 prior to corticosteroid initiation (or the equivalent PMA week for the untreated infant) (categorical: 21%, 22%-30%, 31%-40%, 41%-50%, 51%-99%, 100%)
- Hypotension treatment at less than 24 hours of age

© 2023 Jensen EA et al. *JAMA Network Open.*

- Last recorded highest mode of respiratory support (high frequency ventilation, conventional ventilation, non-invasive intermittent pressure ventilation, nasal continuous positive airway pressure, nasal cannula/oxygen hood/no support) prior to corticosteroid initiation (or the equivalent PMA week for the untreated infant)
- Day of last recorded respiratory support (snapshot) (7, 14, or 28 days of age) prior to corticosteroid initiation (or the equivalent PMA week for the untreated infant) (continuous variable)
- Interactions (all assessed prior to corticosteroid initiation, or the equivalent PMA week for the untreated infant):
    - Invasive mechanical ventilation days × snapshot day
    - Positive airway pressure days × snapshot day
    - Last known FiO_2 × snapshot day
    - Last known mode of respiratory support × snapshot day

Propensity score model covariates:
The propensity score model included the same covariates as the model used to predict the pre-treatment probability of death or grade 2-3 BPD at 36 weeks' PMA. However, the propensity score model also exact (force) matched on the following variables:

- PMA of steroid initiation or the equivalent PMA for the untreated infant) (week)
- Gestational age at birth (week)
- Last recorded highest mode of respiratory support (high frequency ventilation, conventional ventilation, non-invasive intermittent pressure ventilation, nasal continuous positive airway pressure, nasal cannula/oxygen hood/no support) prior to corticosteroid initiation (or the equivalent PMA week for the untreated infant)
- Day of last recorded respiratory support (snapshot) (7, 14, or 28 days of age) prior to corticosteroid initiation (or the equivalent PMA week for the untreated infant)
- Decile of the predicted pre-treatment probability of death or grade 2-3 BPD at 36 weeks' PMA (estimated using in the full eligible cohort)

Prediction of the pre-treatment probability of death or grade 2-3 BPD at 36 weeks' PMA in the matched sample:
The predicted pre-treatment probability of death or grade 2-3 BPD at 36 weeks' PMA was recalculated based on data recorded for the untreated infants in the matched cohort up until the same PMA as the 1:1 matched corticosteroid-treated infants initiated corticosteroid therapy. The same covariates used in the pre-match model were used for the matched infants with the following exceptions:

- PMA week at corticosteroid initiation for the corresponding matched, treated infant was included as a covariate
- Last recorded FiO_2 prior to the PMA week at corticosteroid initiation in the corresponding matched, treated infant was included as a continuous variable
- The model did not include last recorded mode respiratory support × snapshot day interaction

Odds ratios:
Stratifying by the pre-treatment probability of death or grade 2-3 BPD, logistic regression models used to estimate the within-stratum odds ratios for the 2-year corrected age composite outcomes (death or neurodevelopmental impairment and death or cerebral palsy) included the following explanatory (independent) variables:

- Center
- PMA week at corticosteroid initiation (or the equivalent PMA week in the untreated infant)
- Corticosteroid treatment status
- Stratified pre-treatment probability of death or grade 2-3 BPD at 36 weeks' PMA. Strata approximate the quintiles of the distribution: 0 to 0.25, >0.25 to 0.45, >0.45 to 0.65, >0.65 to 0.85, >0.85 to 1.00.
- Corticosteroid therapy status × pre-treatment probability of death or grade 2-3 BPD strata interaction

© 2023 Jensen EA et al. *JAMA Network Open.*

Predicted probability of death or neurodevelopmental impairment (NDI) and death or cerebral palsy (CP) at 2 years' corrected age:

The logistic regression models used to predict the probability of death or NDI and death or CP at 2 years' corrected age included the same covariates as the model used to predict the pre-treatment probability of death or grade 2-3 BPD at 36 weeks' PMA for the matched sample, with the addition of the following explanatory (independent) variables:

- Corticosteroid treatment status
- Predicted pre-treatment probability of death or grade 2-3 BPD at 36 weeks' PMA
- Corticosteroid therapy × pre-treatment probability of death or grade 2-3 BPD at 36 weeks' PMA interaction

© 2023 Jensen EA et al. *JAMA Network Open.*

eTable 1. Characteristics of corticosteroid-treated infants who were excluded owing to missing data and those eligible for matching.

Variable		*Corticosteroid treated – excluded due to missing data N = 90*	*Corticosteroid treated – eligible for matching N = 656*	*Standardized Difference*[a]
Antenatal steroids		79/89 (89%)	597/656 (91%)	0.0744
Antenatal antibiotics		62/84 (74%)	497/656 (76%)	0.0450
C-section		58/90 (64%)	419/656 (64%)	0.0119
Magnesium sulfate		65/85 (76%)	526/656 (80%)	0.0902
Mother's education	< High school	8/90 (9%)	86/656 (13%)	0.2229
	High school	28/90 (31%)	151/656 (23%)	0.2229
	> High school	33/90 (37%)	283/656 (43%)	0.2229
	Missing	21/90 (23%)	136/656 (21%)	0.2229
Mother's race	Black	28/90 (31%)	277/656 (42%)	0.2870
	White	56/90 (62%)	327/656 (50%)	0.2870
	Other	5/90 (6%)	32/656 (5%)	0.2870
	Missing	1/90 (1%)	20/656 (3%)	0.2870
Hispanic origin of mother	Hispanic or Latino	7/90 (8%)	67/656 (10%)	0.0700
	Not Hispanic or Latino	82/90 (91%)	583/656 (89%)	0.0700
	Missing	1/90 (1%)	6/656 (1%)	0.0700
Multiple birth		22/90 (24%)	179/656 (27%)	0.0649
Private insurance		37/90 (41%)	262/656 (40%)	0.0239
Maternal hypertension		20/90 (22%)	174/656 (27%)	0.1003
Birth weight (g), n, mean (SD)		90, 734.8 (160.6)	656, 673.1 (135.3)	0.4154
Birth year	2011	12/90 (13%)	88/656 (13%)	0.3493
	2012	15/90 (17%)	97/656 (15%)	0.3493
	2013	5/90 (6%)	98/656 (15%)	0.3493
	2014	9/90 (10%)	95/656 (14%)	0.3493
	2015	20/90 (22%)	114/656 (17%)	0.3493
	2016	21/90 (23%)	126/656 (19%)	0.3493
	2017	8/90 (9%)	38/656 (6%)	0.3493
Gestational age (wk), n, mean (SD)		90, 24.7 (1.1)	656, 24.3 (1.1)	0.3485
Outborn		5/90 (6%)	9/656 (1%)	0.2303
Male		45/90 (50%)	374/656 (57%)	0.1409

© 2023 Jensen EA et al. *JAMA Network Open.*

eTable 1. Characteristics of corticosteroid-treated infants who were excluded owing to missing data and those eligible for matching.

Variable		*Corticosteroid treated – excluded due to missing data N = 90*	*Corticosteroid treated – eligible for matching N = 656*	*Standardized Difference*[a]
Small for gestational age		4/90 (4%)	61/656 (9%)	0.1928
Chest compressions/epinephrine in the delivery room		10/90 (11%)	50/656 (8%)	0.1200
Intubation in the delivery room		77/90 (86%)	573/656 (87%)	0.0524
Surfactant		86/90 (96%)	634/656 (97%)	0.0564
Chorioamnionitis		9/86 (10%)	128/656 (20%)	0.2555
Treatment for hypotension in the first 24hr after birth		23/90 (26%)	241/656 (37%)	0.2432
Indomethacin in the first 24hr after birth		35/90 (39%)	243/656 (37%)	0.0380
Enteral feeds initiated within 7 days after birth		79/90 (88%)	556/656 (85%)	0.0879
Highest mode of respiratory support at 24hr of age	High frequency ventilation	23/89 (26%)	155/656 (24%)	0.2267
	Conventional ventilation	58/89 (65%)	398/656 (61%)	0.2267
	NIMV	1/89 (1%)	27/656 (4%)	0.2267
	nCPAP	6/89 (7%)	70/656 (11%)	0.2267
	NC, Hood, or No support	1/89 (1%)	6/656 (1%)	0.2267
FiO_2 at 24hr of age	21%	20/89 (22%)	96/656 (15%)	0.2561
	(21%, 30%]	32/89 (36%)	253/656 (39%)	0.2561
	(30%, 40%]	18/89 (20%)	160/656 (24%)	0.2561
	(40%, 50%]	5/89 (6%)	60/656 (9%)	0.2561
	(50%, 100%)	8/89 (9%)	61/656 (9%)	0.2561
	100%	6/89 (7%)	26/656 (4%)	0.2561
Center	3	7/90 (8%)	52/656 (8%)	0.6526
	4	1/90 (1%)	9/656 (1%)	0.6526
	5		5/656 (1%)	0.6526
	9	1/90 (1%)	6/656 (1%)	0.6526
	11	14/90 (16%)	75/656 (11%)	0.6526
	12	4/90 (4%)	17/656 (3%)	0.6526
	14	3/90 (3%)	37/656 (6%)	0.6526

© 2023 Jensen EA et al. *JAMA Network Open.*

eTable 1. Characteristics of corticosteroid-treated infants who were excluded owing to missing data and those eligible for matching.

Variable		*Corticosteroid treated – excluded due to missing data N = 90*	*Corticosteroid treated – eligible for matching N = 656*	*Standardized Difference*[a]
Center, *cont.*	15		14/656 (2%)	0.6526
	16	2/90 (2%)	62/656 (9%)	0.6526
	18	7/90 (8%)	49/656 (7%)	0.6526
	19	8/90 (9%)	93/656 (14%)	0.6526
	24	12/90 (13%)	53/656 (8%)	0.6526
	25	5/90 (6%)	26/656 (4%)	0.6526
	26	1/90 (1%)	14/656 (2%)	0.6526
	27	5/90 (6%)	66/656 (10%)	0.6526
	28	15/90 (17%)	48/656 (7%)	0.6526
	29		6/656 (1%)	0.6526
	30	2/90 (2%)	7/656 (1%)	0.6526
	31	3/90 (3%)	17/656 (3%)	0.6526

[a] Data are absolute values

Abbreviations: FiO_2, fraction of inspired oxygen; n, number of infants with evaluable data for the specified characteristic; NC, nasal cannula; nCPAP, nasal continuous positive airway pressure; NIMV, non-invasive mechanical ventilation; SD, standard deviation

© 2023 Jensen EA et al. *JAMA Network Open.*

eTable 2. Characteristics of untreated infants who were excluded owing to missing data and those eligible for matching.

Variable		*Untreated – excluded due to missing data N = 597*	*Untreated – eligible for matching N = 2796*	*Standardized Difference*[a]
Antenatal corticosteroids		538/592 (91%)	2507/2796 (90%)	0.0410
Antenatal antibiotics		416/566 (73%)	2095/2796 (75%)	0.0327
C-section		357/594 (60%)	1781/2796 (64%)	0.0741
Antenatal magnesium sulfate		455/570 (80%)	2176/2796 (78%)	0.0489
Mother's education	< High school	80/597 (13%)	441/2796 (16%)	0.1554
	High school	144/597 (24%)	689/2796 (25%)	0.1554
	> High school	227/597 (38%)	1131/2796 (40%)	0.1554
	Missing	146/597 (24%)	535/2796 (19%)	0.1554
Mother's race	Black	237/597 (40%)	1243/2796 (44%)	0.1268
	White	286/597 (48%)	1320/2796 (47%)	0.1268
	Other	51/597 (9%)	156/2796 (6%)	0.1268
	Missing	23/597 (4%)	77/2796 (3%)	0.1268
Hispanic origin of mother	Hispanic or Latino	78/597 (13%)	419/2796 (15%)	0.0983
	Not Hispanic or Latino	510/597 (85%)	2346/2796 (84%)	0.0983
	Missing	9/597 (2%)	31/2796 (1%)	0.0983
Multiple birth		138/597 (23%)	695/2796 (25%)	0.0408
Private insurance		206/596 (35%)	1037/2796 (37%)	0.0527
Maternal hypertension		102/588 (17%)	642/2796 (23%)	0.1403
Birth weight (g), n, mean (SD)		597, 809.1 (161.1)	2796, 763 (166.3)	0.2814
Birth year	2011	75/597 (13%)	378/2796 (14%)	0.1324
	2012	113/597 (19%)	493/2796 (18%)	0.1324
	2013	84/597 (14%)	468/2796 (17%)	0.1324
	2014	105/597 (18%)	479/2796 (17%)	0.1324
	2015	99/597 (17%)	481/2796 (17%)	0.1324
	2016	99/597 (17%)	405/2796 (14%)	0.1324
	2017	22/597 (4%)	92/2796 (3%)	0.1324
Gestational age (wk), n, mean (SD)		597, 25.2 (0.9)	2796, 24.9 (1.1)	0.2719

© 2023 Jensen EA et al. *JAMA Network Open.*

eTable 2. Characteristics of untreated infants who were excluded owing to missing data and those eligible for matching.

Variable		*Untreated – excluded due to missing data N = 597*	*Untreated – eligible for matching N = 2796*	*Standardized Difference*[a]
Outborn		37/597 (6%)	52/2796 (2%)	0.2220
Male		306/593 (52%)	1362/2796 (49%)	0.0578
Small for gestational age		20/593 (3%)	160/2796 (6%)	0.1130
Chest compressions/epinephrine in the delivery room		46/596 (8%)	227/2796 (8%)	0.0148
Intubation in the delivery room		445/597 (75%)	2133/2796 (76%)	0.0406
Surfactant		513/597 (86%)	2471/2796 (88%)	0.0732
Chorioamnionitis		90/580 (16%)	472/2796 (17%)	0.0370
Treatment for hypotension in the first 24hr after birth		166/596 (28%)	825/2796 (30%)	0.0366
Indomethacin in the first 24hr after birth		237/597 (40%)	1179/2796 (42%)	0.0502
Enteral feeds initiated within 7 days of age		511/594 (86%)	2460/2796 (88%)	0.0582
Highest mode of respiratory support at 24hr of age	High frequency ventilation	63/584 (11%)	373/2796 (13%)	0.1097
	Conventional ventilation	325/584 (56%)	1581/2796 (57%)	0.1097
	NIMV	36/584 (6%)	183/2796 (7%)	0.1097
	nCPAP	150/584 (26%)	603/2796 (22%)	0.1097
	NC, Hood, or No support	10/584 (2%)	56/2796 (2%)	0.1097
FiO_2 at 24hr of age	21%	190/586 (32%)	860/2796 (31%)	0.1826
	(21%, 30%]	210/586 (36%)	942/2796 (34%)	0.1826
	(30%, 40%]	102/586 (17%)	492/2796 (18%)	0.1826
	(40%, 50%]	41/586 (7%)	217/2796 (8%)	0.1826
	(50%, 100%)	25/586 (4%)	185/2796 (7%)	0.1826
	100%	18/586 (3%)	100/2796 (4%)	0.1826
Center	3	21/597 (4%)	103/2796 (4%)	0.4810
	4	3/597 (1%)	140/2796 (5%)	0.4810
	5	26/597 (4%)	126/2796 (5%)	0.4810

© 2023 Jensen EA et al. *JAMA Network Open.*

eTable 2. Characteristics of untreated infants who were excluded owing to missing data and those eligible for matching.

Variable		*Untreated – excluded due to missing data N = 597*	*Untreated – eligible for matching N = 2796*	*Standardized Difference*[a]
	9	29/597 (5%)	185/2796 (7%)	0.4810
	11	29/597 (5%)	199/2796 (7%)	0.4810
Center, *cont.*	12	19/597 (3%)	94/2796 (3%)	0.4810
	14	26/597 (4%)	147/2796 (5%)	0.4810
	15	31/597 (5%)	93/2796 (3%)	0.4810
	16	49/597 (8%)	287/2796 (10%)	0.4810
	18	59/597 (10%)	258/2796 (9%)	0.4810
	19	68/597 (11%)	318/2796 (11%)	0.4810
	24	32/597 (5%)	164/2796 (6%)	0.4810
	25	7/597 (1%)	21/2796 (1%)	0.4810
	26	7/597 (1%)	47/2796 (2%)	0.4810
	27	22/597 (4%)	180/2796 (6%)	0.4810
	28	83/597 (14%)	153/2796 (5%)	0.4810
	29	6/597 (1%)	29/2796 (1%)	0.4810
	30	73/597 (12%)	195/2796 (7%)	0.4810
	31	7/597 (1%)	57/2796 (2%)	0.4810

[a] Data are absolute values

Abbreviations: FiO_2, fraction of inspired oxygen; n, number of infants with evaluable data for the specified characteristic; NC, nasal cannula; nCPAP, *nasal continuous positive airway pressure; NIMV, non-invasive mechanical ventilation; SD, standard deviation*

© 2023 Jensen EA et al. *JAMA Network Open.*

eTable 3. Characteristics of the matched corticosteroid-treated infants and eligible but unmatched corticosteroid-treated infants (excludes infants with missing data)

Variable		*Matched corticosteroid treated N = 482*	*Unmatched corticosteroid treated N = 174*	*Standardized Difference[a]*
Antenatal corticosteroids		435/482 (90%)	162/174 (93%)	0.1035
Antenatal antibiotics		357/482 (74%)	140/174 (80%)	0.1530
C-section		313/482 (65%)	106/174 (61%)	0.0833
Antenatal magnesium sulfate		385/482 (80%)	141/174 (81%)	0.0292
Mother's education	< High school	63/482 (13%)	23/174 (13%)	0.0613
	High school	114/482 (24%)	37/174 (21%)	0.0613
	> High school	207/482 (43%)	76/174 (44%)	0.0613
	Missing	98/482 (20%)	38/174 (22%)	0.0613
Mother's race	Black	198/482 (41%)	79/174 (45%)	0.2416
	White	242/482 (50%)	85/174 (49%)	0.2416
	Other	29/482 (6%)	3/174 (2%)	0.2416
	Missing	13/482 (3%)	7/174 (4%)	0.2416
Hispanic origin of mother	Hispanic or Latino	53/482 (11%)	14/174 (8%)	0.1048
	Not Hispanic or Latino	425/482 (88%)	158/174 (91%)	0.1048
	Missing	4/482 (1%)	2/174 (1%)	0.1048
Multiple birth		127/482 (26%)	52/174 (30%)	0.0787
Private insurance		190/482 (39%)	72/174 (41%)	0.0400
Maternal hypertension		125/482 (26%)	49/174 (28%)	0.0502
Birth weight (g), n, mean (SD)		482, 682.1 (134.9)	174, 648.2 (133.7)	0.2525
Birth year	2011	63/482 (13%)	25/174 (14%)	0.1401
	2012	75/482 (16%)	22/174 (13%)	0.1401
	2013	71/482 (15%)	27/174 (16%)	0.1401
	2014	74/482 (15%)	21/174 (12%)	0.1401
	2015	82/482 (17%)	32/174 (18%)	0.1401
	2016	90/482 (19%)	36/174 (21%)	0.1401
	2017	27/482 (6%)	11/174 (6%)	0.1401
Gestational age (wk), n, mean (SD)		482, 24.4 (1.1)	174, 24.2 (1.2)	0.1263

© 2023 Jensen EA et al. *JAMA Network Open.*

eTable 3. Characteristics of the matched corticosteroid-treated infants and eligible but unmatched corticosteroid-treated infants (excludes infants with missing data)

Variable		*Matched corticosteroid treated N = 482*	*Unmatched corticosteroid treated N = 174*	*Standardized Difference*[a]
Outborn		8/482 (2%)	1/174 (1%)	0.1034
Male		270/482 (56%)	104/174 (60%)	0.0761
Small for gestational age		41/482 (9%)	20/174 (11%)	0.0997
Chest compressions/epinephrine in the delivery room		40/482 (8%)	10/174 (6%)	0.1000
Intubation in the delivery room		415/482 (86%)	158/174 (91%)	0.1476
Surfactant		463/482 (96%)	171/174 (98%)	0.1340
Chorioamnionitis		85/482 (18%)	43/174 (25%)	0.1739
Treatment for hypotension in the first 24hr after birth		191/482 (40%)	50/174 (29%)	0.2311
Indomethacin in the first 24hr after birth		182/482 (38%)	61/174 (35%)	0.0562
Enteral feeds initiated within 7 days of age		411/482 (85%)	145/174 (83%)	0.0532
Highest mode of respiratory support at 24hr of age	High frequency ventilation	117/482 (24%)	38/174 (22%)	0.1411
	Conventional ventilation	285/482 (59%)	113/174 (65%)	0.1411
	NIMV	22/482 (5%)	5/174 (3%)	0.1411
	nCPAP	53/482 (11%)	17/174 (10%)	0.1411
	NC, Hood, or No support	5/482 (1%)	1/174 (1%)	0.1411
FiO_2 at 24hr of age	21%	71/482 (15%)	25/174 (14%)	0.1011
	(21%, 30%]	185/482 (38%)	68/174 (39%)	0.1011
	(30%, 40%]	118/482 (24%)	42/174 (24%)	0.1011
	(40%, 50%]	47/482 (10%)	13/174 (7%)	0.1011
	(50%, 100%)	43/482 (9%)	18/174 (10%)	0.1011
	100%	18/482 (4%)	8/174 (5%)	0.1011
Center	3	35/482 (7%)	17/174 (10%)	0.7039
	4	9/482 (2%)	0/174 (0%)	0.7039
	5	5/482 (1%)	0/174 (0%)	0.7039

© 2023 Jensen EA et al. *JAMA Network Open.*

eTable 3. Characteristics of the matched corticosteroid-treated infants and eligible but unmatched corticosteroid-treated infants (excludes infants with missing data)

Variable		*Matched corticosteroid treated N = 482*	*Unmatched corticosteroid treated N = 174*	*Standardized Difference*[a]
	9	6/482 (1%)	0/174 (0%)	0.7039
	11	49/482 (10%)	26/174 (15%)	0.7039
Center, *cont.*	12	17/482 (4%)	0/174 (0%)	0.7039
	14	23/482 (5%)	14/174 (8%)	0.7039
	15	14/482 (3%)	0/174 (0%)	0.7039
	16	42/482 (9%)	20/174 (11%)	0.7039
	18	39/482 (8%)	10/174 (6%)	0.7039
	19	65/482 (13%)	28/174 (16%)	0.7039
	24	49/482 (10%)	4/174 (2%)	0.7039
	25	17/482 (4%)	9/174 (5%)	0.7039
	26	9/482 (2%)	5/174 (3%)	0.7039
	27	46/482 (10%)	20/174 (11%)	0.7039
	28	36/482 (7%)	12/174 (7%)	0.7039
	29	6/482 (1%)	0/174 (0%)	0.7039
	30	6/482 (1%)	1/174 (1%)	0.7039
	31	9/482 (2%)	8/174 (5%)	0.7039

[a] Data are absolute values

Abbreviations: FiO_2, fraction of inspired oxygen; n, number of infants with evaluable data for the specified characteristic; NC, nasal cannula; nCPAP, nasal continuous positive airway pressure; NIMV, non-invasive mechanical ventilation; SD, standard deviation

© 2023 Jensen EA et al. *JAMA Network Open.*

eTable 4. Characteristics of corticosteroid-treated infants and untreated controls eligible for matching (excludes infants with missing data)

Variable		*Corticosteroid treated N = 656*	*Untreated potential controls N = 2796*	*Standardized Difference*[a]
Antenatal corticosteroids		597/656 (91%)	2507/2796 (90%)	0.0454
Antenatal antibiotics		497/656 (76%)	2095/2796 (75%)	0.0193
C-section		419/656 (64%)	1781/2796 (64%)	0.0036
Antenatal magnesium sulfate		526/656 (80%)	2176/2796 (78%)	0.0579
Mother's education	< High school	86/656 (13%)	441/2796 (16%)	0.0951
	High school	151/656 (23%)	689/2796 (25%)	0.0951
	> High school	283/656 (43%)	1131/2796 (40%)	0.0951
	Missing	136/656 (21%)	535/2796 (19%)	0.0951
Mother's race	Black	277/656 (42%)	1243/2796 (44%)	0.0619
	White	327/656 (50%)	1320/2796 (47%)	0.0619
	Other	32/656 (5%)	156/2796 (6%)	0.0619
	Missing	20/656 (3%)	77/2796 (3%)	0.0619
Hispanic origin of mother	Hispanic or Latino	67/656 (10%)	419/2796 (15%)	0.1463
	Not Hispanic or Latino	583/656 (89%)	2346/2796 (84%)	0.1463
	Missing	6/656 (1%)	31/2796 (1%)	0.1463
Multiple birth		179/656 (27%)	695/2796 (25%)	0.0554
Private insurance		262/656 (40%)	1037/2796 (37%)	0.0586
Maternal hypertension		174/656 (27%)	642/2796 (23%)	0.0826
Birth weight (g), n, mean (SD)		656, 673.1 (135.3)	2796, 763.0 (166.3)	0.5930
Birth year	2011	88/656 (13%)	378/2796 (14%)	0.1972
	2012	97/656 (15%)	493/2796 (18%)	0.1972
	2013	98/656 (15%)	468/2796 (17%)	0.1972
	2014	95/656 (14%)	479/2796 (17%)	0.1972
	2015	114/656 (17%)	481/2796 (17%)	0.1972
	2016	126/656 (19%)	405/2796 (14%)	0.1972
	2017	38/656 (6%)	92/2796 (3%)	0.1972
Gestational age (wk), n, mean (SD)		656, 24.3 (1.1)	2796, 24.9 (1.1)	0.5690
Outborn		9/656 (1%)	52/2796 (2%)	0.0387

eTable 4. Characteristics of corticosteroid-treated infants and untreated controls eligible for matching (excludes infants with missing data)

Variable		*Corticosteroid treated N = 656*	*Untreated potential controls N = 2796*	*Standardized Difference*[a]
Male		374/656 (57%)	1362/2796 (49%)	0.1668
Small for gestational age		61/656 (9%)	160/2796 (6%)	0.1360
Chest compressions/epinephrine in the delivery room		50/656 (8%)	227/2796 (8%)	0.0184
Intubation in the delivery room		573/656 (87%)	2133/2796 (76%)	0.2897
Surfactant		634/656 (97%)	2471/2796 (88%)	0.3182
Chorioamnionitis		128/656 (20%)	472/2796 (17%)	0.0682
Treatment for hypotension in the first 24hr after birth		241/656 (37%)	825/2796 (30%)	0.1541
Indomethacin in the first 24hr after birth		243/656 (37%)	1179/2796 (42%)	0.1049
Enteral feeds initiated within 7 days of age		556/656 (85%)	2460/2796 (88%)	0.0941
Highest mode of respiratory support at 24hr of age	High frequency ventilation	155/656 (24%)	373/2796 (13%)	0.3989
	Conventional ventilation	398/656 (61%)	1581/2796 (57%)	0.3989
	NIMV	27/656 (4%)	183/2796 (7%)	0.3989
	nCPAP	70/656 (11%)	603/2796 (22%)	0.3989
	NC, Hood, or No support	6/656 (1%)	56/2796 (2%)	0.3989
FiO_2 at 24hr of age	21%	96/656 (15%)	860/2796 (31%)	0.4013
	(21%, 30%]	253/656 (39%)	942/2796 (34%)	0.4013
	(30%, 40%]	160/656 (24%)	492/2796 (18%)	0.4013
	(40%, 50%]	60/656 (9%)	217/2796 (8%)	0.4013
	(50%, 100%)	61/656 (9%)	185/2796 (7%)	0.4013
	100%	26/656 (4%)	100/2796 (4%)	0.4013
Center	3	52/656 (8%)	103/2796 (4%)	0.6772
	4	9/656 (1%)	140/2796 (5%)	0.6772
	5	5/656 (1%)	126/2796 (5%)	0.6772
	9	6/656 (1%)	185/2796 (7%)	0.6772
	11	75/656 (11%)	199/2796 (7%)	0.6772

© 2023 Jensen EA et al. *JAMA Network Open.*

eTable 4. Characteristics of corticosteroid-treated infants and untreated controls eligible for matching (excludes infants with missing data)

Variable		*Corticosteroid treated N = 656*	*Untreated potential controls N = 2796*	*Standardized Difference*[a]
Center, *cont.*	12	17/656 (3%)	94/2796 (3%)	0.6772
	14	37/656 (6%)	147/2796 (5%)	0.6772
	15	14/656 (2%)	93/2796 (3%)	0.6772
	16	62/656 (9%)	287/2796 (10%)	0.6772
	18	49/656 (7%)	258/2796 (9%)	0.6772
	19	93/656 (14%)	318/2796 (11%)	0.6772
	24	53/656 (8%)	164/2796 (6%)	0.6772
	25	26/656 (4%)	21/2796 (1%)	0.6772
	26	14/656 (2%)	47/2796 (2%)	0.6772
	27	66/656 (10%)	180/2796 (6%)	0.6772
	28	48/656 (7%)	153/2796 (5%)	0.6772
	29	6/656 (1%)	29/2796 (1%)	0.6772
	30	7/656 (1%)	195/2796 (7%)	0.6772
	31	17/656 (3%)	57/2796 (2%)	0.6772

[a] Data are absolute values

Abbreviations: FiO_2, fraction of inspired oxygen; n, number of infants with evaluable data for the specified characteristic; NC, nasal cannula; nCPAP, nasal continuous positive airway pressure; NIMV, non-invasive mechanical ventilation; SD, standard deviation

© 2023 Jensen EA et al. *JAMA Network Open.*

eTable 5. Characteristics of corticosteroid-treated infants and untreated controls in the final matched cohort

Variable		*Corticosteroid Treated N = 482*	*Untreated controls N = 482*	*Standardized Difference*[a]
Antenatal steroids		435/482 (90%)	427/482 (89%)	0.0540
Antenatal antibiotics		357/482 (74%)	345/482 (72%)	0.0560
C-section		313/482 (65%)	311/482 (65%)	0.0087
Antenatal magnesium sulfate		385/482 (80%)	373/482 (77%)	0.0608
Mother's education	< High school	63/482 (13%)	60/482 (12%)	0.0852
	High school	114/482 (24%)	99/482 (21%)	0.0852
	> High school	207/482 (43%)	223/482 (46%)	0.0852
	Missing	98/482 (20%)	100/482 (21%)	0.0852
Mother's race	Black	198/482 (41%)	201/482 (42%)	0.0675
	White	242/482 (50%)	247/482 (51%)	0.0675
	Other	29/482 (6%)	25/482 (5%)	0.0675
	Missing	13/482 (3%)	9/482 (2%)	0.0675
Hispanic origin of mother	Hispanic or Latino	53/482 (11%)	54/482 (11%)	0.0251
	Not Hispanic or Latino	425/482 (88%)	425/482 (88%)	0.0251
	Missing	4/482 (1%)	3/482 (1%)	0.0251
Multiple birth		127/482 (26%)	127/482 (26%)	0.0000
Private insurance		190/482 (39%)	194/482 (40%)	0.0170
Maternal hypertension		125/482 (26%)	133/482 (28%)	0.0375
Birth weight (g), n, mean (SD)		482, 682.1 (134.9)	482, 671.5 (146)	0.0756
Birth year	2011	63/482 (13%)	63/482 (13%)	0.1468
	2012	75/482 (16%)	70/482 (15%)	0.1468
	2013	71/482 (15%)	82/482 (17%)	0.1468
	2014	74/482 (15%)	84/482 (17%)	0.1468
	2015	82/482 (17%)	89/482 (18%)	0.1468
	2016	90/482 (19%)	77/482 (16%)	0.1468
	2017	27/482 (6%)	17/482 (4%)	0.1468
Gestational age (wk), n, mean (SD)		482, 24.4 (1.1)	482, 24.4 (1.1)	0.0000
Outborn		8/482 (2%)	10/482 (2%)	0.0307
Male		270/482 (56%)	270/482 (56%)	0.0000
Small for gestational age		41/482 (9%)	51/482 (11%)	0.0707

© 2023 Jensen EA et al. *JAMA Network Open.*

eTable 5. Characteristics of corticosteroid-treated infants and untreated controls in the final matched cohort

Variable		*Corticosteroid Treated N = 482*	*Untreated controls N = 482*	*Standardized Difference*[a]
Chest compressions/epinephrine in the delivery room		40/482 (8%)	41/482 (9%)	0.0075
Intubation in the delivery room		415/482 (86%)	426/482 (88%)	0.0684
Surfactant		463/482 (96%)	469/482 (97%)	0.0695
Chorioamnionitis		85/482 (18%)	78/482 (16%)	0.0388
Hypotension treatment in the first 24hr of age		191/482 (40%)	180/482 (37%)	0.0469
Indomethacin in the first 24hr of age		182/482 (38%)	187/482 (39%)	0.0213
Enteral feeds initiated within 7 days of age		411/482 (85%)	415/482 (86%)	0.0237
Highest mode of respiratory support at 24hr of age	High frequency ventilation	117/482 (24%)	124/482 (26%)	0.1128
	Conventional ventilation	285/482 (59%)	292/482 (61%)	0.1128
	NIMV	22/482 (5%)	15/482 (3%)	0.1128
	nCPAP	53/482 (11%)	49/482 (10%)	0.1128
	NC, Hood, or No support	5/482 (1%)	2/482 (0%)	0.1128
FiO_2 at 24hr of age	21%	71/482 (15%)	94/482 (20%)	0.1645
	22% to 30%	185/482 (38%)	176/482 (37%)	0.1645
	31% to 40%	118/482 (24%)	98/482 (20%)	0.1645
	41% to 50%	47/482 (10%)	45/482 (9%)	0.1645
	51% to 99%	43/482 (9%)	44/482 (9%)	0.1645
	100%	18/482 (4%)	25/482 (5%)	0.1645
Grade 3-4 intraventricular hemorrhage		68/482 (14%)	69/482 (14%)	0.0059
Surgical necrotizing enterocolitis or spontaneous intestinal perforation		35/482 (7%)	41/482 (9%)	0.0462
Culture confirmed sepsis or meningitis		102/482 (21%)	92/482 (19%)	0.0518
Treatment with nitric oxide		66/482 (14%)	68/482 (14%)	0.0120
Last recorded FiO_2, n, mean (SD)	21% to 30%	56/482 (12%)	57/482 (12%)	0.0345
	31% to 40%	105/482 (22%)	101/482 (21%)	0.0345
	41% to 50%	82/482 (17%)	85/482 (18%)	0.0345
	51% to 99%	172/482 (36%)	171/482 (35%)	0.0345

© 2023 Jensen EA et al. *JAMA Network Open.*

eTable 5. Characteristics of corticosteroid-treated infants and untreated controls in the final matched cohort

Variable		*Corticosteroid Treated N = 482*	*Untreated controls N = 482*	*Standardized Difference*[a]
Last recorded FiO_2, *cont.*	100%	62/482 (13%)	64/482 (13%)	0.0345
Last recorded highest mode of respiratory support	High frequency ventilation	213/482 (44%)	213/482 (44%)	0.0000
	Conventional ventilation	240/482 (50%)	240/482 (50%)	0.0000
	NIMV	14/482 (3%)	14/482 (3%)	0.0000
	nCPAP	14/482 (3%)	14/482 (3%)	0.0000
	NC, Hood, or No support	1/482 (0%)	1/482 (0%)	0.0000
Days on invasive ventilation, n, mean (SD)		482, 18.6 (7.8)	482, 18.5 (8)	0.0058
Days on positive airway pressure, n, mean (SD)		482, 20.4 (7.7)	482, 20.4 (7.6)	0.0016
Propensity score, n, mean (SD)		482, 0.52 (0.25)	482, 0.5 (0.24)	0.0839
Pre-treatment probability of death or grade 2-3 BPD, n, mean (SD)		482, 0.53 (0.22)	482, 0.53 (0.22)	0.0126
PMA at corticosteroid initiation (or equivalent for controls) (wk), n, mean (SD)		482, 27.82 (1.73)	482, 27.82 (1.73)	0.0000
PMA at corticosteroid initiation (or equivalent for controls) (wk)	23	3/482 (1%)	3/482 (1%)	0.0000
	24	10/482 (2%)	10/482 (2%)	0.0000
	25	31/482 (6%)	31/482 (6%)	0.0000
	26	68/482 (14%)	68/482 (14%)	0.0000
	27	89/482 (18%)	89/482 (18%)	0.0000
	28	108/482 (22%)	108/482 (22%)	0.0000
	29	94/482 (20%)	94/482 (20%)	0.0000
	30	50/482 (10%)	50/482 (10%)	0.0000
	31	25/482 (5%)	25/482 (5%)	0.0000
	32	4/482 (1%)	4/482 (1%)	0.0000
Day of respiratory support snapshot used in the analysis	Day 7	27/482 (6%)	27/482 (6%)	0.0000
	Day 14	211/482 (44%)	211/482 (44%)	0.0000
	Day 28	244/482 (51%)	244/482 (51%)	0.0000
Center	1	35/482 (7%)	26/482 (5%)	0.1647
	2	9/482 (2%)	11/482 (2%)	0.1647

© 2023 Jensen EA et al. *JAMA Network Open.*

eTable 5. Characteristics of corticosteroid-treated infants and untreated controls in the final matched cohort

Variable		*Corticosteroid Treated N = 482*	*Untreated controls N = 482*	*Standardized Difference*[a]
	3	5/482 (1%)	5/482 (1%)	0.1647
Center, *cont.*	4	6/482 (1%)	9/482 (2%)	0.1647
	5	49/482 (10%)	49/482 (10%)	0.1647
	6	17/482 (4%)	21/482 (4%)	0.1647
	7	23/482 (5%)	22/482 (5%)	0.1647
	8	14/482 (3%)	11/482 (2%)	0.1647
	9	42/482 (9%)	41/482 (9%)	0.1647
	10	39/482 (8%)	47/482 (10%)	0.1647
	11	65/482 (13%)	63/482 (13%)	0.1647
	12	49/482 (10%)	53/482 (11%)	0.1647
	13	17/482 (4%)	12/482 (2%)	0.1647
	14	9/482 (2%)	9/482 (2%)	0.1647
	15	46/482 (10%)	39/482 (8%)	0.1647
	16	36/482 (7%)	37/482 (8%)	0.1647
	17	6/482 (1%)	7/482 (1%)	0.1647
	18	6/482 (1%)	6/482 (1%)	0.1647
	19	9/482 (2%)	14/482 (3%)	0.1647

[a] Data are absolute values

Abbreviations: BPD, bronchopulmonary dysplasia; FiO_2, fraction of inspired oxygen; n, number of infants with evaluable data for the specified characteristic; NC, nasal cannula; nCPAP, nasal continuous positive airway pressure; NIMV, non-invasive mechanical ventilation; SD, standard deviation; PMA, postmenstrual age

© 2023 Jensen EA et al. *JAMA Network Open.*

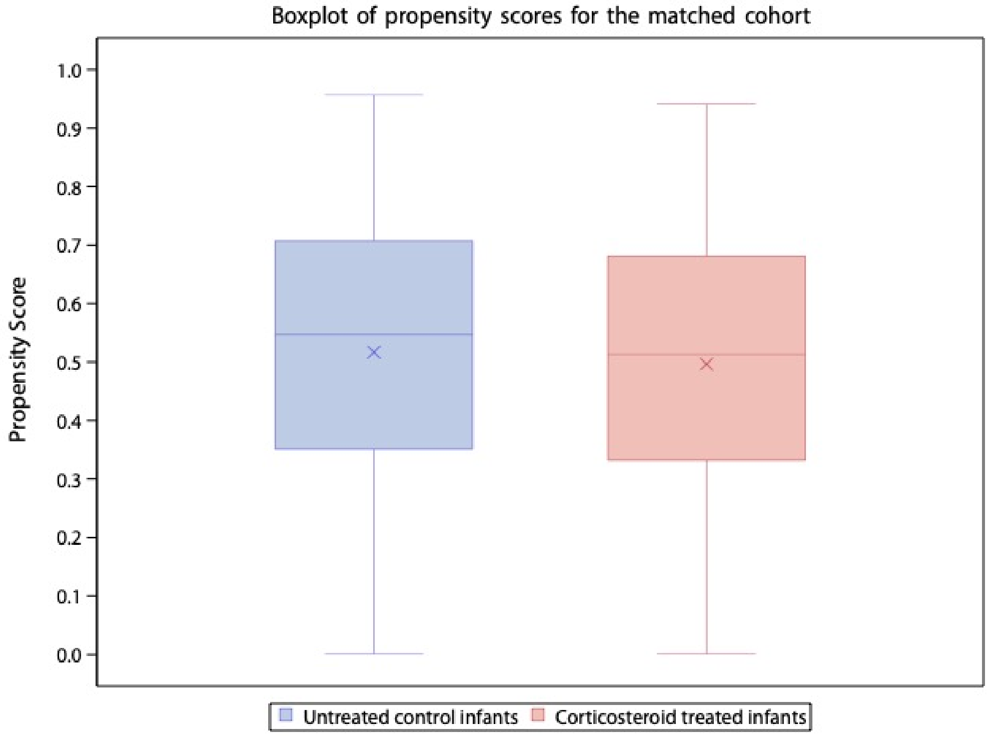

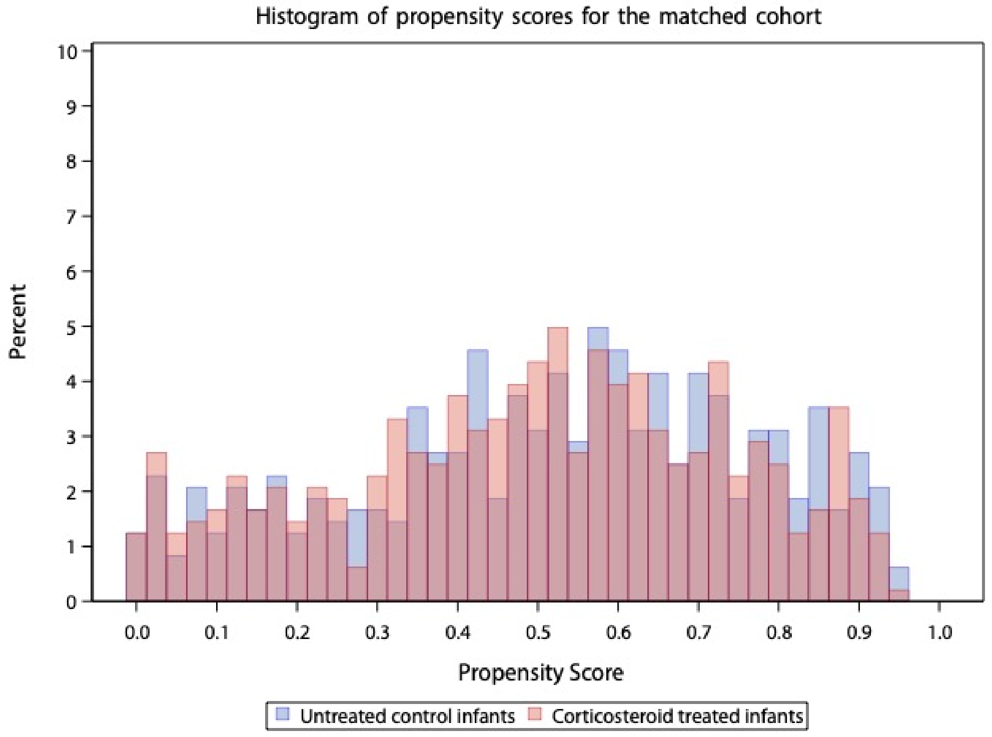

eFigure 1. Distribution of propensity scores in the matched cohort.

© 2023 Jensen EA et al. *JAMA Network Open.*

Box plots (top) and histograms (bottom) showing propensity score values calculated in the 482 corticosteroid-treated infants (red) and 482 untreated controls (blue) in the matched study cohort. The x in the top plot displays the mean propensity score values in the two groups.

© 2023 Jensen EA et al. *JAMA Network Open.*

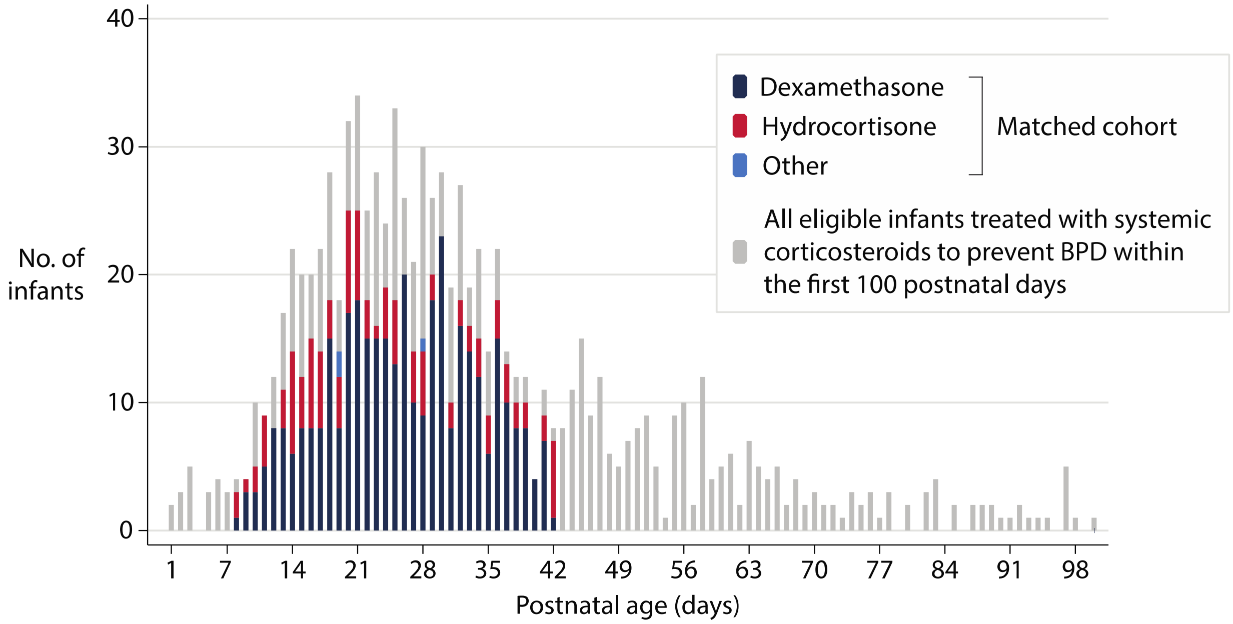

eFigure 2. Distribution of the timing of corticosteroid initiation.
The stacked colored bars represent the number of corticosteroid-treated infants in the matched cohort who initiated corticosteroid treatment between postnatal days 8 and 42, stratified by corticosteroid type. The mean (standard deviation) age at corticosteroid initiation among matched infants was 25.2 (8.5) days. The underlaid gray bars represent the total number of corticosteroid-treated infants who were eligible for the study and initiated corticosteroid therapy within the first 100 postnatal days.

© 2023 Jensen EA et al. *JAMA Network Open.*

eTable 6. Rates and timing of mortality in the matched cohort

Outcome	*Corticosteroid-treated infants N = 482*	*Untreated control infants N = 482*
Died prior to 36 weeks' PMA	49 (10.2)	103 (21.4)
Died between 36 weeks' PMA and 2-year follow-up	44 (9.1)	15 (3.1)
Survived to 2-year follow-up	389 (80.7)	364 (75.5)

Values displayed as n (%); PMA, postmenstrual age

© 2023 Jensen EA et al. *JAMA Network Open.*

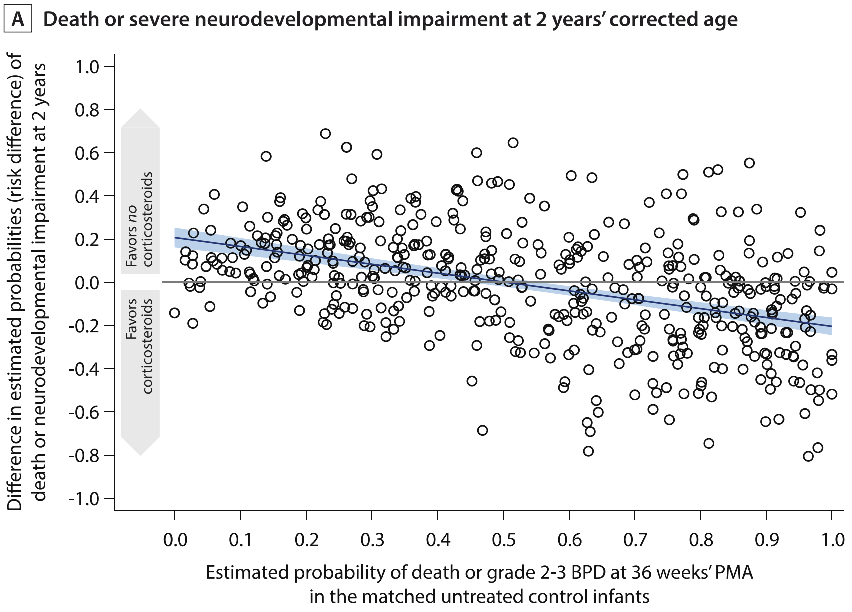

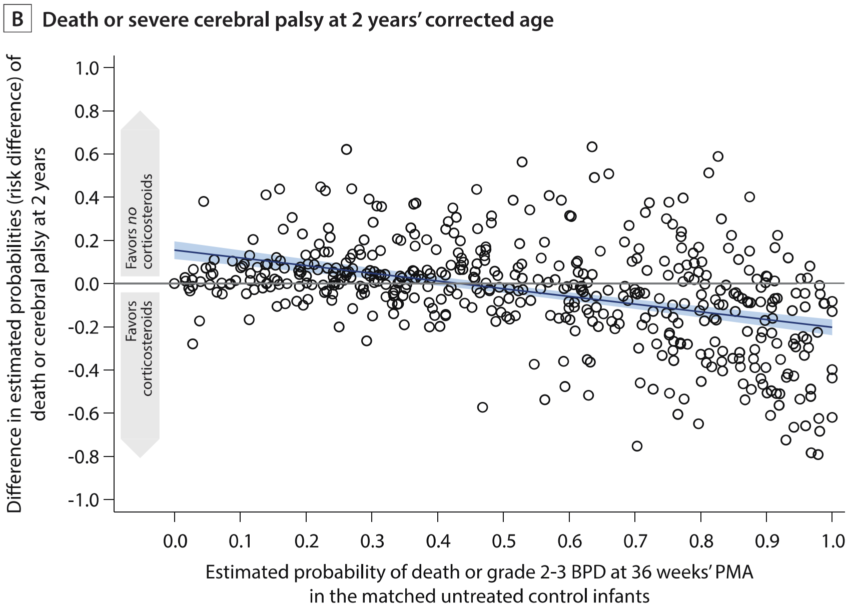

eFigure 3. Risk differences for death or severe disability versus the probability of death or grade 2-3 bronchopulmonary dysplasia (BPD) at 36 weeks' postmenstrual age (PMA).
Plot (A) shows the results for death or severe neurodevelopmental impairment (NDI) at 2 years' corrected age. Plot (B) shows the results for death or severe cerebral palsy (CP) at 2 years' corrected age. The probability of death or grade 2-3 BPD was estimated using data collected up until the time of corticosteroid initiation or the equivalent PMA week in the matched untreated control. Circles represent values computed for individual matched pairs. The fitted regression lines and their shaded 95% confidence intervals are shown. *Death or severe NDI:* For each 10% increase in the pre-treatment probability of death or grade 2-3 BPD, the risk difference for death or severe NDI decreased by 4.1% (95% CI 3.4% - 4.9%; regression line equation: y=0.207 – 0.411x). The fitted regression line crosses the x-axis at a probability of death or grade 2-3 BPD of 50% (95% confidence bands cross at 45% and 55%). *Death or severe CP:* For each 10% increase in the pre-treatment probability of death or grade 2-3 BPD, the risk difference for death or severe CP decreased by 3.6% (95% CI 2.9% - 4.2%; regression line

© 2023 Jensen EA et al. *JAMA Network Open.*

equation: y=0.155 – 0.357x). The regression line crosses the x-axis at a pre-treatment probability of death of grade 2-3 BPD of 43% (95% confidence bands cross at 37% and 49%).

© 2023 Jensen EA et al. *JAMA Network Open.*

A Death or moderate to severe neurodevelopmental impairment at 2 years' corrected age

Probability of death or grade 2-3 BPD	Matched pairs, n	Observed outcome rates, n (%) Corticosteroid treated	Observed outcome rates, n (%) Untreated controls	Adjusted OR (95% CI)
Full cohort	470	323 (68.7)	323 (68.7)	0.92 (0.68-1.24)
Subgroups				
0% to 25%	95	55 (57.9)	50 (52.6)	1.12 (0.61-2.04)
>25% to 45%	101	69 (68.3)	59 (58.4)	1.28 (0.70-2.33)
>45% to 65%	92	61 (66.3)	64 (69.6)	0.97 (0.51-1.84)
>65% to 85%	94	73 (77.7)	75 (79.8)	0.91 (0.44-1.89)
>85% to 100%	88	65 (73.9)	75 (85.2)	0.53 (0.24-1.16)

test of interaction p=0.51

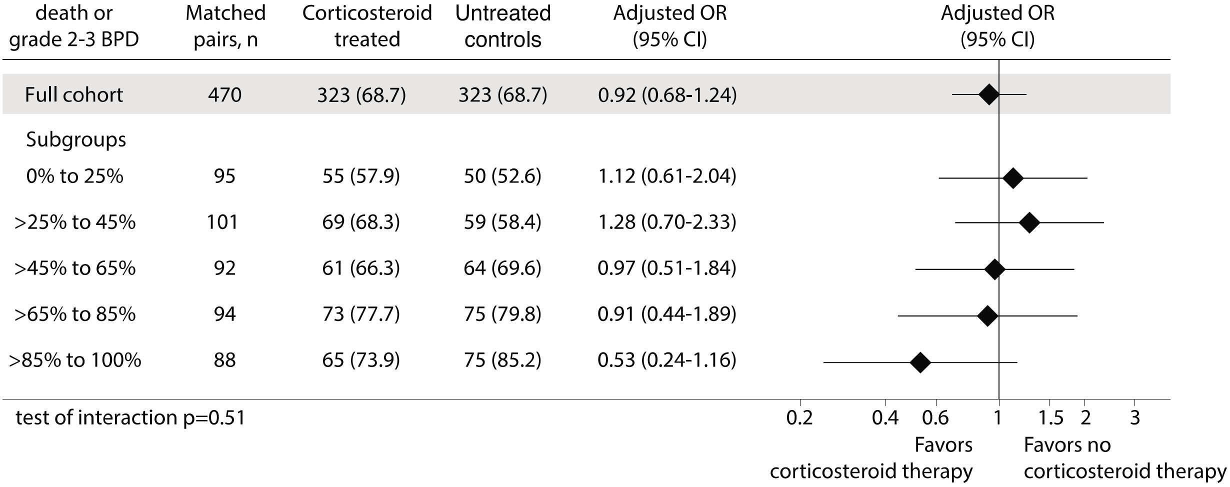

B Death or moderate to severe cerebral palsy at 2 years' corrected age

Probability of death or grade 2-3 BPD	Matched pairs, n	Observed outcome rates, n (%) Corticosteroid treated	Observed outcome rates, n (%) Untreated controls	Adjusted OR (95% CI)
Full cohort	482	130 (27.0)	154 (32.0)	0.83 (0.60-1.15)
Subgroups				
0% to 25%	95	14 (14.7)	12 (12.6)	1.14 (0.48-2.69)
>25% to 45%	105	19 (18.1)	17 (16.2)	1.04 (0.49-2.20)
>45% to 65%	95	27 (28.4)	22 (23.2)	1.49 (0.74-2.98)
>65% to 85%	98	37 (37.8)	46 (46.9)	0.66 (0.36-1.24)
>85% to 100%	89	33 (37.1)	57 (64.0)	0.34 (0.17-0.67)

test of interaction p=0.03

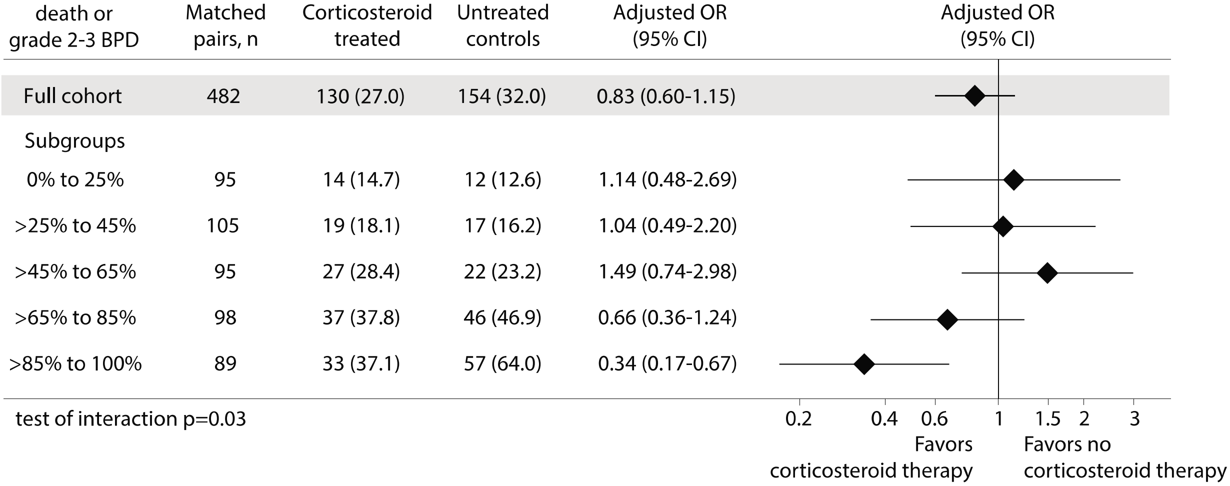

eFigure 4. Odds ratios for death or moderate-to-severe disability stratified by the pre-treatment probability of death or grade 2-3 BPD as a 5-level categorical variable.
Observed rates and corresponding adjusted odds ratios (OR) with 95% confidence intervals (CI) for (A) death or moderate-to-severe neurodevelopmental impairment at 2 years' corrected age and (B) death or moderate-to-severe cerebral palsy by 2 years' corrected age, in the full matched cohort and stratified by the estimated pre-treatment probability of death or grade 2-3 bronchopulmonary dysplasia (BPD) at 36 weeks' postmenstrual age (PMA). OR were estimated using logistic regression and adjusted for the following explanatory variables: corticosteroid treatment status, pre-treatment probability of early death or grade 2-3 BPD strata, corticosteroid treatment status × pre-treatment probability stratum interaction term, PMA week at corticosteroid initiation or the equivalent week in the untreated matched control, and center.
n, number of infants

© 2023 Jensen EA et al. *JAMA Network Open.*

A **Death or severe neurodevelopmental impairment at 2 years' corrected age**

Probability of death or grade 2-3 BPD	Matched pairs, n	Observed outcome rates, n (%) Corticosteroid treated	Observed outcome rates, n (%) Untreated controls	Adjusted OR (95% CI)
Full cohort	467	198 (42.4)	198 (42.4)	0.94 (0.70-1.24)
Subgroups				
0% to 25%	95	25 (26.3)	20 (21.1)	1.29 (0.65-2.56)
>25% to 45%	101	46 (45.5)	27 (26.7)	1.83 (1.01-3.31)
>45% to 65%	90	37 (41.1)	33 (36.7)	1.19 (0.64-2.21)
>65% to 85%	94	51 (54.3)	57 (60.6)	0.75 (0.41-1.39)
>85% to 100%	87	39 (44.8)	61 (70.1)	0.34 (0.17-0.66)

test of interaction p=0.004

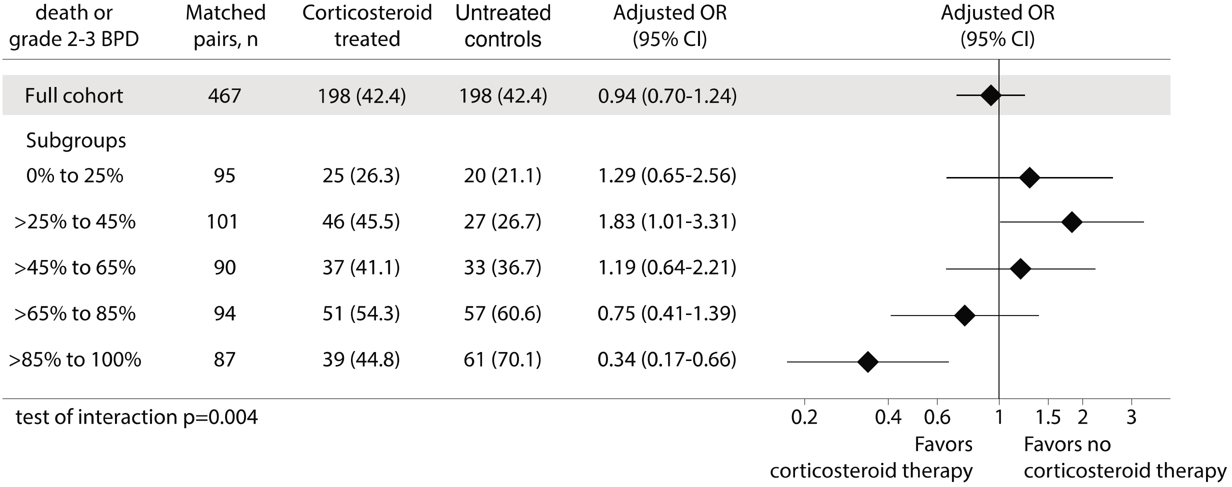

B **Death or severe cerebral palsy at 2 years' corrected age**

Probability of death or grade 2-3 BPD	Matched pairs, n	Observed outcome rates, n (%) Corticosteroid treated	Observed outcome rates, n (%) Untreated controls	Adjusted OR (95% CI)
Full cohort	482	109 (22.6)	127 (26.3)	0.94 (0.65-1.33)
Subgroups				
0% to 25%	95	12 (12.6)	10 (10.5)	1.22 (0.48-3.08)
>25% to 45%	105	17 (16.2)	11 (10.5)	1.57 (0.68-3.65)
>45% to 65%	95	17 (17.9)	15 (15.8)	1.26 (0.56-2.80)
>65% to 85%	98	32 (32.7)	39 (39.8)	0.73 (0.38-1.38)
>85% to 100%	89	31 (34.8)	52 (58.4)	0.41 (021-0.82)

test of interaction p=0.09

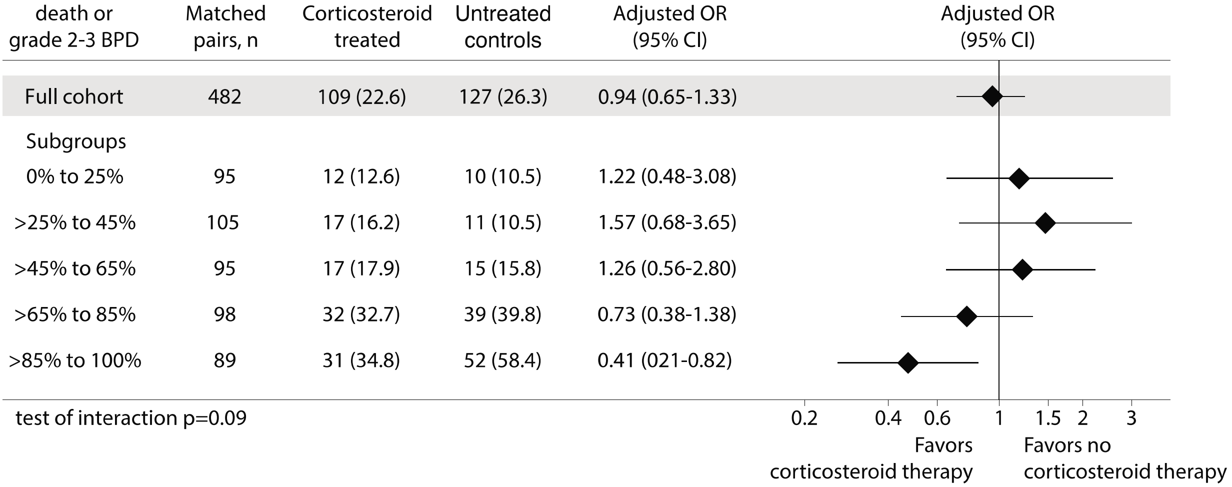

eFigure 5. Odds ratios for death or severe disability stratified by the pre-treatment probability of death or grade 2-3 BPD as a 5-level categorical variable.
Observed rates and corresponding adjusted odds ratios (OR) with 95% confidence intervals (CI) for (A) death or severe neurodevelopmental impairment at 2 years' corrected age and (B) death or severe cerebral palsy by 2 years' corrected age, in the full matched cohort and stratified by the estimated pre-treatment probability of death or grade 2-3 bronchopulmonary dysplasia (BPD) at 36 weeks' postmenstrual age (PMA). Odds ratios were estimated using logistic regression and adjusted for the following explanatory variables: corticosteroid treatment status, pre-treatment probability of early death or grade 2-3 BPD strata, corticosteroid treatment status × pre-treatment probability stratum interaction term, PMA week at corticosteroid initiation or the equivalent week in the untreated matched control, and center.
n, number of infants

© 2023 Jensen EA et al. *JAMA Network Open.*

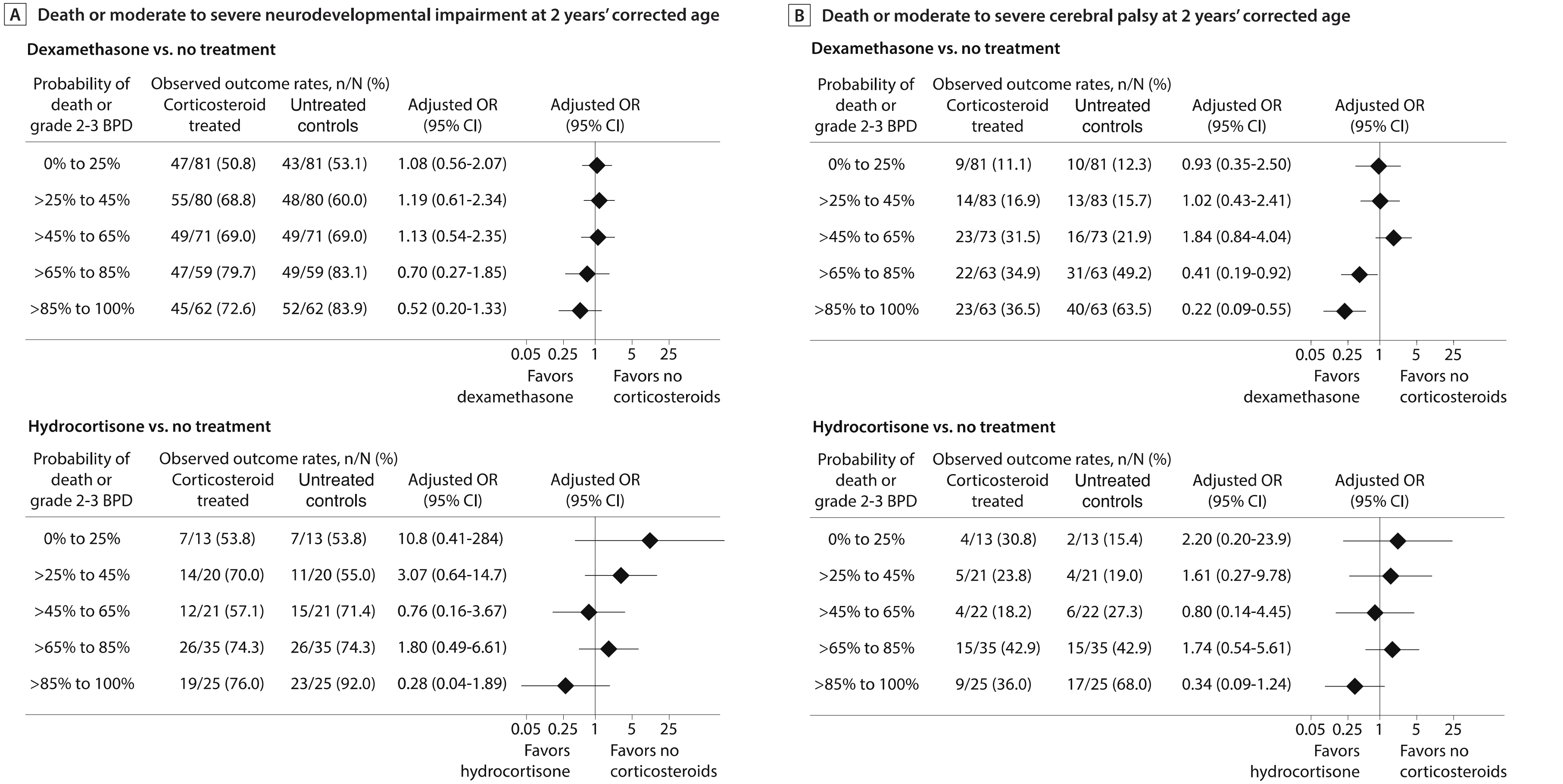

eFigure 6. Odds ratios for death or moderate to severe disability stratified by treatment with dexamethasone vs. hydrocortisone.

Observed rates and corresponding adjusted odds ratios (OR) with 95% confidence intervals (CI) for (A) death or moderate-to-severe neurodevelopmental impairment at 2 years' corrected age and (B) death or moderate-to-severe cerebral palsy by 2 years' corrected age, stratified by the estimated pre-treatment probability of death or grade 2-3 bronchopulmonary dysplasia (BPD) at 36 weeks' postmenstrual age (PMA) and by treatment with dexamethasone (top) or hydrocortisone (bottom). Observed outcome rates are reported for infants with a known 2-year outcome. ORs estimated using logistic regression and adjusted for the following explanatory variables: corticosteroid treatment status, pre-treatment probability of death or grade 2-3 BPD strata, corticosteroid treatment status × pre-treatment probability stratum interaction, PMA week at corticosteroid initiation or the equivalent week in the untreated matched control, and center.

© 2023 Jensen EA et al. *JAMA Network Open.*

n, number of infants with the specified outcome; N, number of infants with evaluable data for the specified outcome.

© 2023 Jensen EA et al. *JAMA Network Open.*
