# Supplementary material for: Assessment of Corticosteroid Therapy and Death or Disability According to Pretreatment Risk of Death or Bronchopulmonary Dysplasia in Extremely Preterm Infants
Source: JAMA Netw Open. 2023 May 8;6(5):e2312277. doi: 10.1001/jamanetworkopen.2023.12277 (PMC10167571; doi:10.1001/jamanetworkopen.2023.12277)
Supplement: Supplement 2. — Nonauthor Contributors [file jamanetwopen-e2312277-s002.pdf]

*Indicates required information. Only first name, last name, and suffix will appear in PubMed.

| *Group Name(s): Eunice Kennedy Shriver National Institute of Child Health and Human Development Neonatal Research Network | | | | | | | |
|---|---|---|---|---|---|---|---|
| *First Name and Middle Initial(s) | *Last Name | *Suffix (eg, Jr, III) | Academic Degrees | Institution | Location (city, state/province, country) | Role or Contribution, eg, chair, principal investigator | Group (if more than 1 Group listed in the byline) and/or Subgroup (eg, Steering Committee) |
| Richard A. | Polin | | MD | College of Physicians and Surgeons, Columbia University | New York, New York | Chair | Eunice Kennedy Shriver National Institute of Child Health and Human Development Neonatal Research Network |
| Abbot R. | Laptook | | MD | Alpert School of Brown University and Women & Infant's Hospital of Rhode Island | Providence, Rhode Island | Non-Author Contributor | Eunice Kennedy Shriver National Institute of Child Health and Human Development Neonatal Research Network |
| Martin | Keszler | | MD | Alpert School of Brown University and Women & Infant's Hospital of Rhode Island | Providence, Rhode Island | Non-Author Contributor | Eunice Kennedy Shriver National Institute of Child Health and Human Development Neonatal Research Network |
| Betty R. | Vohr | | MD | Alpert School of Brown University and Women & Infant's Hospital of Rhode Island | Providence, Rhode Island | Non-Author Contributor | Eunice Kennedy Shriver National Institute of Child Health and Human Development Neonatal Research Network |
| Angelita M. | Hensman | | PhD RNC-NIC | Alpert School of Brown University and Women & Infant's Hospital of Rhode Island | Providence, Rhode Island | Non-Author Contributor | Eunice Kennedy Shriver National Institute of Child Health and Human Development Neonatal Research Network |
| Elisa | Vieira | | RN BSN | Alpert School of Brown University and Women & Infant's Hospital of Rhode Island | Providence, Rhode Island | Non-Author Contributor | Eunice Kennedy Shriver National Institute of Child Health and Human Development Neonatal Research Network |
| Lucille St. | Pierre | | BS | Alpert School of Brown University and Women & Infant's Hospital of Rhode Island | Providence, Rhode Island | Non-Author Contributor | Eunice Kennedy Shriver National Institute of Child Health and Human Development Neonatal Research Network |
| Robert T. | Burke | | MD MPH | Alpert School of Brown University and Women & Infant's Hospital of Rhode Island | Providence, Rhode Island | Non-Author Contributor | Eunice Kennedy Shriver National Institute of Child Health and Human Development Neonatal Research Network |
| Barbara | Alksninis | | RNC PNP | Alpert School of Brown University and Women & Infant's Hospital of Rhode Island | Providence, Rhode Island | Non-Author Contributor | Eunice Kennedy Shriver National Institute of Child Health and Human Development Neonatal Research Network |
| Andrea | Knoll | | | Alpert School of Brown University and Women & Infant's Hospital of Rhode Island | Providence, Rhode Island | Non-Author Contributor | Eunice Kennedy Shriver National Institute of Child Health and Human Development Neonatal Research Network |
| Mary L. | Keszler | | MD | Alpert School of Brown University and Women & Infant's Hospital of Rhode Island | Providence, Rhode Island | Non-Author Contributor | Eunice Kennedy Shriver National Institute of Child Health and Human Development Neonatal Research Network |
| Teresa M. | Leach | | MEd CAES | Alpert School of Brown University and Women & Infant's Hospital of Rhode Island | Providence, Rhode Island | Non-Author Contributor | Eunice Kennedy Shriver National Institute of Child Health and Human Development Neonatal Research Network |
| Elisabeth C. | McGowan | | MD | Alpert School of Brown University and Women & Infant's Hospital of Rhode Island | Providence, Rhode Island | Non-Author Contributor | Eunice Kennedy Shriver National Institute of Child Health and Human Development Neonatal Research Network |
| Victoria E. | Watson | | MS CAS | Alpert School of Brown University and Women & Infant's Hospital of Rhode Island | Providence, Rhode Island | Non-Author Contributor | Eunice Kennedy Shriver National Institute of Child Health and Human Development Neonatal Research Network |

Supplemental Online Content: Nonauthor Collaborators
*Indicates required information. Only first name, last name, and suffix will appear in PubMed.

| ***First Name and Middle Initial(s)** | ***Last Name** | ***Suffix (eg, Jr, III)** | Academic Degrees | Institution | Location (city, state/province, country) | Role or Contribution, eg, chair, principal investigator | Group (if more than 1 Group listed in the byline) and/or Subgroup (eg, Steering Committee) |
|---|---|---|---|---|---|---|---|
| Anna Maria | Hibbs | | MD MSCE | Case Western Reserve University, Rainbow Babies & Children's Hospital | Cleveland, Ohio | Non-Author Contributor | Eunice Kennedy Shriver National Institute of Child Health and Human Development Neonatal Research Network |
| Nancy S. | Newman | | RN | Case Western Reserve University, Rainbow Babies & Children's Hospital | Cleveland, Ohio | Non-Author Contributor | Eunice Kennedy Shriver National Institute of Child Health and Human Development Neonatal Research Network |
| Deanne E. | Wilson-Costello | | MD | Case Western Reserve University, Rainbow Babies & Children's Hospital | Cleveland, Ohio | Non-Author Contributor | Eunice Kennedy Shriver National Institute of Child Health and Human Development Neonatal Research Network |
| Bonnie S. | Siner | | RN | Case Western Reserve University, Rainbow Babies & Children's Hospital | Cleveland, Ohio | Non-Author Contributor | Eunice Kennedy Shriver National Institute of Child Health and Human Development Neonatal Research Network |
| Harriet G. | Friedman | | MA | Case Western Reserve University, Rainbow Babies & Children's Hospital | Cleveland, Ohio | Non-Author Contributor | Eunice Kennedy Shriver National Institute of Child Health and Human Development Neonatal Research Network |
| William E. | Truog | | MD | Children's Mercy Hospital | Kansas City, MO | Non-Author Contributor | Eunice Kennedy Shriver National Institute of Child Health and Human Development Neonatal Research Network |
| Eugenia K. | Pallotto | | MD MSCE | Children's Mercy Hospital | Kansas City, MO | Non-Author Contributor | Eunice Kennedy Shriver National Institute of Child Health and Human Development Neonatal Research Network |
| Howard W. | Kilbride | | MD | Children's Mercy Hospital | Kansas City, MO | Non-Author Contributor | Eunice Kennedy Shriver National Institute of Child Health and Human Development Neonatal Research Network |
| Cheri | Gauldin | | RN BS CCRC | Children's Mercy Hospital | Kansas City, MO | Non-Author Contributor | Eunice Kennedy Shriver National Institute of Child Health and Human Development Neonatal Research Network |
| Anne | Holmes | | RN MSN MBA-HCM CCRC | Children's Mercy Hospital | Kansas City, MO | Non-Author Contributor | Eunice Kennedy Shriver National Institute of Child Health and Human Development Neonatal Research Network |
| Kathy | Johnson | | RN CCRC | Children's Mercy Hospital | Kansas City, MO | Non-Author Contributor | Eunice Kennedy Shriver National Institute of Child Health and Human Development Neonatal Research Network |
| Allison | Scott | | BSN RNC-NIC | Children's Mercy Hospital | Kansas City, MO | Non-Author Contributor | Eunice Kennedy Shriver National Institute of Child Health and Human Development Neonatal Research Network |
| Prabhu S. | Parimi | | MD | Children's Mercy Hospital | Kansas City, MO | Non-Author Contributor | Eunice Kennedy Shriver National Institute of Child Health and Human Development Neonatal Research Network |
| Lisa | Gaetano | | RN MSN | Children's Mercy Hospital | Kansas City, MO | Non-Author Contributor | Eunice Kennedy Shriver National Institute of Child Health and Human Development Neonatal Research Network |
| Stephani L. | Merhar | | MD MS | Cincinnati Children's Hospital Medical Center, University Hospital, and Good Samaritan Hospital | Cincinnati, Ohio | Non-Author Contributor | Eunice Kennedy Shriver National Institute of Child Health and Human Development Neonatal Research Network |

| *First Name and Middle Initial(s) | *Last Name | *Suffix (eg, Jr, III) | Academic Degrees | Institution | Location (city, state/province, country) | Role or Contribution, eg, chair, principal investigator | Group (if more than 1 Group listed in the byline) and/or Subgroup (eg, Steering Committee) |
|---|---|---|---|---|---|---|---|
| Kurt | Schibler | | MD | Cincinnati Children's Hospital Medical Center, University Hospital, and Good Samaritan Hospital | Cincinnati, Ohio | Non-Author Contributor | Eunice Kennedy Shriver National Institute of Child Health and Human Development Neonatal Research Network |
| Brenda B. | Poindexter | | MD MS | Cincinnati Children's Hospital Medical Center, University Hospital, and Good Samaritan Hospital | Cincinnati, Ohio | Non-Author Contributor | Eunice Kennedy Shriver National Institute of Child Health and Human Development Neonatal Research Network |
| Kimberly | Yolton | | PhD | Cincinnati Children's Hospital Medical Center, University Hospital, and Good Samaritan Hospital | Cincinnati, Ohio | Non-Author Contributor | Eunice Kennedy Shriver National Institute of Child Health and Human Development Neonatal Research Network |
| Tanya E. | Cahill | | MD | Cincinnati Children's Hospital Medical Center, University Hospital, and Good Samaritan Hospital | Cincinnati, Ohio | Non-Author Contributor | Eunice Kennedy Shriver National Institute of Child Health and Human Development Neonatal Research Network |
| Teresa L. | Gratton | | PA | Cincinnati Children's Hospital Medical Center, University Hospital, and Good Samaritan Hospital | Cincinnati, Ohio | Non-Author Contributor | Eunice Kennedy Shriver National Institute of Child Health and Human Development Neonatal Research Network |
| Cathy | Grisby | | BSN CCRC | Cincinnati Children's Hospital Medical Center, University Hospital, and Good Samaritan Hospital | Cincinnati, Ohio | Non-Author Contributor | Eunice Kennedy Shriver National Institute of Child Health and Human Development Neonatal Research Network |
| Kristin | Kirker | | CRC | Cincinnati Children's Hospital Medical Center, University Hospital, and Good Samaritan Hospital | Cincinnati, Ohio | Non-Author Contributor | Eunice Kennedy Shriver National Institute of Child Health and Human Development Neonatal Research Network |
| Sandra | Wuertz | | RN BSN CLC | Cincinnati Children's Hospital Medical Center, University Hospital, and Good Samaritan Hospital | Cincinnati, Ohio | Non-Author Contributor | Eunice Kennedy Shriver National Institute of Child Health and Human Development Neonatal Research Network |
| Michael C. | Cotten | | MD MHS | Duke University SOM, University Hospital, University of North Carolina, Duke Regional Hospital, and WakeMed Health & Hospitals | Durham, Chapel Hill, and Raleigh, NC | Non-Author Contributor | Eunice Kennedy Shriver National Institute of Child Health and Human Development Neonatal Research Network |
| Ronald N. | Goldberg | | MD | Duke University SOM, University Hospital, University of North Carolina, Duke Regional Hospital, and WakeMed Health & Hospitals | Durham, Chapel Hill, and Raleigh, NC | Non-Author Contributor | Eunice Kennedy Shriver National Institute of Child Health and Human Development Neonatal Research Network |
| Ricki F. | Goldstein | | MD | Duke University SOM, University Hospital, University of North Carolina, Duke Regional Hospital, and WakeMed Health & Hospitals | Durham, Chapel Hill, and Raleigh, NC | Non-Author Contributor | Eunice Kennedy Shriver National Institute of Child Health and Human Development Neonatal Research Network |
| William F. | Malcolm | | MD | Duke University SOM, University Hospital, University of North Carolina, Duke Regional Hospital, and WakeMed Health & Hospitals | Durham, Chapel Hill, and Raleigh, NC | Non-Author Contributor | Eunice Kennedy Shriver National Institute of Child Health and Human Development Neonatal Research Network |
| Patricia L. | Ashley | | MD | Duke University SOM, University Hospital, University of North Carolina, Duke Regional Hospital, and WakeMed Health & Hospitals | Durham, Chapel Hill, and Raleigh, NC | Non-Author Contributor | Eunice Kennedy Shriver National Institute of Child Health and Human Development Neonatal Research Network |
| Deesha | Mago-Shah | | MD | Duke University SOM, University Hospital, University of North Carolina, Duke Regional Hospital, and WakeMed Health & Hospitals | Durham, Chapel Hill, and Raleigh, NC | Non-Author Contributor | Eunice Kennedy Shriver National Institute of Child Health and Human Development Neonatal Research Network |
| Joanne | Finkle | | RN JD | Duke University SOM, University Hospital, University of North Carolina, Duke Regional Hospital, and WakeMed Health & Hospitals | Durham, Chapel Hill, and Raleigh, NC | Non-Author Contributor | Eunice Kennedy Shriver National Institute of Child Health and Human Development Neonatal Research Network |

*Indicates required information. Only first name, last name, and suffix will appear in PubMed.

| ***First Name and Middle Initial(s)** | ***Last Name** | ***Suffix (eg, Jr, III)** | Academic Degrees | Institution | Location (city, state/province, country) | Role or Contribution, eg, chair, principal investigator | Group (if more than 1 Group listed in the byline) and/or Subgroup (eg, Steering Committee) |
|---|---|---|---|---|---|---|---|
| Kimberley A. | Fisher | | PhD FNP-BC IBCLC | Duke University SOM, University Hospital, University of North Carolina, Duke Regional Hospital, and WakeMed Health & Hospitals | Durham, Chapel Hill, and Raleigh, NC | Non-Author Contributor | Eunice Kennedy Shriver National Institute of Child Health and Human Development Neonatal Research Network |
| Kathryn E. | Gustafson | | PhD | Duke University SOM, University Hospital, University of North Carolina, Duke Regional Hospital, and WakeMed Health & Hospitals | Durham, Chapel Hill, and Raleigh, NC | Non-Author Contributor | Eunice Kennedy Shriver National Institute of Child Health and Human Development Neonatal Research Network |
| Carl L. | Bose | | MD | Duke University SOM, University Hospital, University of North Carolina, Duke Regional Hospital, and WakeMed Health & Hospitals | Durham, Chapel Hill, and Raleigh, NC | Non-Author Contributor | Eunice Kennedy Shriver National Institute of Child Health and Human Development Neonatal Research Network |
| Janice | Bernhardt | | MS RN | Duke University SOM, University Hospital, University of North Carolina, Duke Regional Hospital, and WakeMed Health & Hospitals | Durham, Chapel Hill, and Raleigh, NC | Non-Author Contributor | Eunice Kennedy Shriver National Institute of Child Health and Human Development Neonatal Research Network |
| Gennie | Bose | | RN | Duke University SOM, University Hospital, University of North Carolina, Duke Regional Hospital, and WakeMed Health & Hospitals | Durham, Chapel Hill, and Raleigh, NC | Non-Author Contributor | Eunice Kennedy Shriver National Institute of Child Health and Human Development Neonatal Research Network |
| Janice | Wereszczak | | CPNP-AC/PC | Duke University SOM, University Hospital, University of North Carolina, Duke Regional Hospital, and WakeMed Health & Hospitals | Durham, Chapel Hill, and Raleigh, NC | Non-Author Contributor | Eunice Kennedy Shriver National Institute of Child Health and Human Development Neonatal Research Network |
| Diane | Warner | | MD MPH | Duke University SOM, University Hospital, University of North Carolina, Duke Regional Hospital, and WakeMed Health & Hospitals | Durham, Chapel Hill, and Raleigh, NC | Non-Author Contributor | Eunice Kennedy Shriver National Institute of Child Health and Human Development Neonatal Research Network |
| Jennifer | Talbert | | MS RN | Duke University SOM, University Hospital, University of North Carolina, Duke Regional Hospital, and WakeMed Health & Hospitals | Durham, Chapel Hill, and Raleigh, NC | Non-Author Contributor | Eunice Kennedy Shriver National Institute of Child Health and Human Development Neonatal Research Network |
| Stephen D. | Kicklighter | | MD | Duke University SOM, University Hospital, University of North Carolina, Duke Regional Hospital, and WakeMed Health & Hospitals | Durham, Chapel Hill, and Raleigh, NC | Non-Author Contributor | Eunice Kennedy Shriver National Institute of Child Health and Human Development Neonatal Research Network |
| Alexandra | Bentley | | MD | Duke University SOM, University Hospital, University of North Carolina, Duke Regional Hospital, and WakeMed Health & Hospitals | Durham, Chapel Hill, and Raleigh, NC | Non-Author Contributor | Eunice Kennedy Shriver National Institute of Child Health and Human Development Neonatal Research Network |
| Laura | Edwards | | MD | Duke University SOM, University Hospital, University of North Carolina, Duke Regional Hospital, and WakeMed Health & Hospitals | Durham, Chapel Hill, and Raleigh, NC | Non-Author Contributor | Eunice Kennedy Shriver National Institute of Child Health and Human Development Neonatal Research Network |
| Ginger | Rhodes-Ryan | | ARNP MSN NNP-BC | Duke University SOM, University Hospital, University of North Carolina, Duke Regional Hospital, and WakeMed Health & Hospitals | Durham, Chapel Hill, and Raleigh, NC | Non-Author Contributor | Eunice Kennedy Shriver National Institute of Child Health and Human Development Neonatal Research Network |
| Donna | White | | RN-BC BSN | Duke University SOM, University Hospital, University of North Carolina, Duke Regional Hospital, and WakeMed Health & Hospitals | Durham, Chapel Hill, and Raleigh, NC | Non-Author Contributor | Eunice Kennedy Shriver National Institute of Child Health and Human Development Neonatal Research Network |
| Ravi M. | Patel | | MD MSc | Emory University, Children's Healthcare of Atlanta, Grady Memorial, and Emory University Hospital Midtown | Atlanta, Georgia | Non-Author Contributor | Eunice Kennedy Shriver National Institute of Child Health and Human Development Neonatal Research Network |
| David P. | Carlton | | MD | Emory University, Children's Healthcare of Atlanta, Grady Memorial, and Emory University Hospital Midtown | Atlanta, Georgia | Non-Author Contributor | Eunice Kennedy Shriver National Institute of Child Health and Human Development Neonatal Research Network |

*Indicates required information. Only first name, last name, and suffix will appear in PubMed.

| ***First Name and Middle Initial(s)** | ***Last Name** | ***Suffix (eg, Jr, III)** | Academic Degrees | Institution | Location (city, state/province, country) | Role or Contribution, eg, chair, principal investigator | Group (if more than 1 Group listed in the byline) and/or Subgroup (eg, Steering Committee) |
|---|---|---|---|---|---|---|---|
| Yvonne | Loggins | | RN | Emory University, Children's Healthcare of Atlanta, Grady Memorial, and Emory University Hospital Midtown | Atlanta, Georgia | Non-Author Contributor | Eunice Kennedy Shriver National Institute of Child Health and Human Development Neonatal Research Network |
| Ira | Adams-Chapman (deceased) | | MD | Emory University, Children's Healthcare of Atlanta, Grady Memorial, and Emory University Hospital Midtown | Atlanta, Georgia | Non-Author Contributor | Eunice Kennedy Shriver National Institute of Child Health and Human Development Neonatal Research Network |
| Diane | Bottcher | | RN | Emory University, Children's Healthcare of Atlanta, Grady Memorial, and Emory University Hospital Midtown | Atlanta, Georgia | Non-Author Contributor | Eunice Kennedy Shriver National Institute of Child Health and Human Development Neonatal Research Network |
| Sheena L. | Carter | | PhD | Emory University, Children's Healthcare of Atlanta, Grady Memorial, and Emory University Hospital Midtown | Atlanta, Georgia | Non-Author Contributor | Eunice Kennedy Shriver National Institute of Child Health and Human Development Neonatal Research Network |
| Ellen C. | Hale | | BS RN CCRC | Emory University, Children's Healthcare of Atlanta, Grady Memorial, and Emory University Hospital Midtown | Atlanta, Georgia | Non-Author Contributor | Eunice Kennedy Shriver National Institute of Child Health and Human Development Neonatal Research Network |
| Salathiel | Kendrick-Allwood | | MD | Emory University, Children's Healthcare of Atlanta, Grady Memorial, and Emory University Hospital Midtown | Atlanta, Georgia | Non-Author Contributor | Eunice Kennedy Shriver National Institute of Child Health and Human Development Neonatal Research Network |
| Maureen | Mulligan LaRossa | | RN | Emory University, Children's Healthcare of Atlanta, Grady Memorial, and Emory University Hospital Midtown | Atlanta, Georgia | Non-Author Contributor | Eunice Kennedy Shriver National Institute of Child Health and Human Development Neonatal Research Network |
| Colleen | Mackie | | RRT | Emory University, Children's Healthcare of Atlanta, Grady Memorial, and Emory University Hospital Midtown | Atlanta, Georgia | Non-Author Contributor | Eunice Kennedy Shriver National Institute of Child Health and Human Development Neonatal Research Network |
| Amy | Sanders | | PsyD | Emory University, Children's Healthcare of Atlanta, Grady Memorial, and Emory University Hospital Midtown | Atlanta, Georgia | Non-Author Contributor | Eunice Kennedy Shriver National Institute of Child Health and Human Development Neonatal Research Network |
| Gloria | Smikle | | PNP | Emory University, Children's Healthcare of Atlanta, Grady Memorial, and Emory University Hospital Midtown | Atlanta, Georgia | Non-Author Contributor | Eunice Kennedy Shriver National Institute of Child Health and Human Development Neonatal Research Network |
| Lynn | Wineski | | NNP | Emory University, Children's Healthcare of Atlanta, Grady Memorial, and Emory University Hospital Midtown | Atlanta, Georgia | Non-Author Contributor | Eunice Kennedy Shriver National Institute of Child Health and Human Development Neonatal Research Network |
| Andrew A. | Bremer | | MD PhD | Eunice Kennedy Shriver National Institute of Child Health and Human Development | Bethesda, MD | Non-Author Contributor | Eunice Kennedy Shriver National Institute of Child Health and Human Development Neonatal Research Network |
| Rosemary D. | Higgins | | MD | Eunice Kennedy Shriver National Institute of Child Health and Human Development | Bethesda, MD | Non-Author Contributor | Eunice Kennedy Shriver National Institute of Child Health and Human Development Neonatal Research Network |
| Stephanie | Wilson Archer | | MA | Eunice Kennedy Shriver National Institute of Child Health and Human Development | Bethesda, MD | Non-Author Contributor | Eunice Kennedy Shriver National Institute of Child Health and Human Development Neonatal Research Network |
| Gregory M. | Sokol | | MD | Indiana University, University Hospital, Methodist Hospital, Riley Hospital for Children, and Wishard Health Services | Indianapolis, IN | Non-Author Contributor | Eunice Kennedy Shriver National Institute of Child Health and Human Development Neonatal Research Network |

*Indicates required information. Only first name, last name, and suffix will appear in PubMed.

| ***First Name and Middle Initial(s)** | ***Last Name** | ***Suffix (eg, Jr, III)** | Academic Degrees | Institution | Location (city, state/province, country) | Role or Contribution, eg, chair, principal investigator | Group (if more than 1 Group listed in the byline) and/or Subgroup (eg, Steering Committee) |
|---|---|---|---|---|---|---|---|
| Brenda B. | Poindexter | | MD MS | Indiana University, University Hospital, Methodist Hospital, Riley Hospital for Children, and Wishard Health Services | Indianapolis, IN | Non-Author Contributor | Eunice Kennedy Shriver National Institute of Child Health and Human Development Neonatal Research Network |
| Heidi | Harmon | | MD MS | Indiana University, University Hospital, Methodist Hospital, Riley Hospital for Children, and Wishard Health Services | Indianapolis, IN | Non-Author Contributor | Eunice Kennedy Shriver National Institute of Child Health and Human Development Neonatal Research Network |
| Lu Ann | Papile | | MD | Indiana University, University Hospital, Methodist Hospital, Riley Hospital for Children, and Wishard Health Services | Indianapolis, IN | Non-Author Contributor | Eunice Kennedy Shriver National Institute of Child Health and Human Development Neonatal Research Network |
| Dianne E. | Herron | | RN CCRC | Indiana University, University Hospital, Methodist Hospital, Riley Hospital for Children, and Wishard Health Services | Indianapolis, IN | Non-Author Contributor | Eunice Kennedy Shriver National Institute of Child Health and Human Development Neonatal Research Network |
| Abbey C. | Hines | | PsyD | Indiana University, University Hospital, Methodist Hospital, Riley Hospital for Children, and Wishard Health Services | Indianapolis, IN | Non-Author Contributor | Eunice Kennedy Shriver National Institute of Child Health and Human Development Neonatal Research Network |
| Carolyn | Lytle | | MD MPH | Indiana University, University Hospital, Methodist Hospital, Riley Hospital for Children, and Wishard Health Services | Indianapolis, IN | Non-Author Contributor | Eunice Kennedy Shriver National Institute of Child Health and Human Development Neonatal Research Network |
| Lucy | Smiley | | CCRC | Indiana University, University Hospital, Methodist Hospital, Riley Hospital for Children, and Wishard Health Services | Indianapolis, IN | Non-Author Contributor | Eunice Kennedy Shriver National Institute of Child Health and Human Development Neonatal Research Network |
| Leslie Dawn | Wilson | | BSN CCRC | Indiana University, University Hospital, Methodist Hospital, Riley Hospital for Children, and Wishard Health Services | Indianapolis, IN | Non-Author Contributor | Eunice Kennedy Shriver National Institute of Child Health and Human Development Neonatal Research Network |
| Jon E. | Tyson | | MD MPH | McGovern Medical School at The University of Texas Health Science Center at Houson, Children's Memorial Hermann Hospital, and Memorial Hermann Southwest Hospital | Houston, TX | Non-Author Contributor | Eunice Kennedy Shriver National Institute of Child Health and Human Development Neonatal Research Network |
| Amir M. | Khan | | MD | McGovern Medical School at The University of Texas Health Science Center at Houson, Children's Memorial Hermann Hospital, and Memorial Hermann Southwest Hospital | Houston, TX | Non-Author Contributor | Eunice Kennedy Shriver National Institute of Child Health and Human Development Neonatal Research Network |
| Kathleen A. | Kennedy | | MD MPH | McGovern Medical School at The University of Texas Health Science Center at Houson, Children's Memorial Hermann Hospital, and Memorial Hermann Southwest Hospital | Houston, TX | Non-Author Contributor | Eunice Kennedy Shriver National Institute of Child Health and Human Development Neonatal Research Network |
| Barbara J. | Stoll | | MD | McGovern Medical School at The University of Texas Health Science Center at Houson, Children's Memorial Hermann Hospital, and Memorial Hermann Southwest Hospital | Houston, TX | Non-Author Contributor | Eunice Kennedy Shriver National Institute of Child Health and Human Development Neonatal Research Network |
| Riciardo A. | Mosquera | | MD MS | McGovern Medical School at The University of Texas Health Science Center at Houson, Children's Memorial Hermann Hospital, and Memorial Hermann Southwest Hospital | Houston, TX | Non-Author Contributor | Eunice Kennedy Shriver National Institute of Child Health and Human Development Neonatal Research Network |
| Andrea F. | Duncan | | MD | McGovern Medical School at The University of Texas Health Science Center at Houson, Children's Memorial Hermann Hospital, and Memorial Hermann Southwest Hospital | Houston, TX | Non-Author Contributor | Eunice Kennedy Shriver National Institute of Child Health and Human Development Neonatal Research Network |
| Emily | Stephens | | BSN RNC-NIC | McGovern Medical School at The University of Texas Health Science Center at Houson, Children's Memorial Hermann Hospital, and Memorial Hermann Southwest Hospital | Houston, TX | Non-Author Contributor | Eunice Kennedy Shriver National Institute of Child Health and Human Development Neonatal Research Network |

| *First Name and Middle Initial(s) | *Last Name | *Suffix (eg, Jr, III) | Academic Degrees | Institution | Location (city, state/province, country) | Role or Contribution, eg, chair, principal investigator | Group (if more than 1 Group listed in the byline) and/or Subgroup (eg, Steering Committee) |
|---|---|---|---|---|---|---|---|
| Elizabeth | Allain | | PhD | McGovern Medical School at The University of Texas Health Science Center at Houson, Children's Memorial Hermann Hospital, and Memorial Hermann Southwest Hospital | Houston, TX | Non-Author Contributor | Eunice Kennedy Shriver National Institute of Child Health and Human Development Neonatal Research Network |
| Julie | Arldt-McAlister | | RN BSN | McGovern Medical School at The University of Texas Health Science Center at Houson, Children's Memorial Hermann Hospital, and Memorial Hermann Southwest Hospital | Houston, TX | Non-Author Contributor | Eunice Kennedy Shriver National Institute of Child Health and Human Development Neonatal Research Network |
| Fatima | Boricha | | MD | McGovern Medical School at The University of Texas Health Science Center at Houson, Children's Memorial Hermann Hospital, and Memorial Hermann Southwest Hospital | Houston, TX | Non-Author Contributor | Eunice Kennedy Shriver National Institute of Child Health and Human Development Neonatal Research Network |
| Katrina | Burson | | RN BSN | McGovern Medical School at The University of Texas Health Science Center at Houson, Children's Memorial Hermann Hospital, and Memorial Hermann Southwest Hospital | Houston, TX | Non-Author Contributor | Eunice Kennedy Shriver National Institute of Child Health and Human Development Neonatal Research Network |
| Allison G. | Dempsey | | PhD | McGovern Medical School at The University of Texas Health Science Center at Houson, Children's Memorial Hermann Hospital, and Memorial Hermann Southwest Hospital | Houston, TX | Non-Author Contributor | Eunice Kennedy Shriver National Institute of Child Health and Human Development Neonatal Research Network |
| Patricia W. | Evans | | MD | McGovern Medical School at The University of Texas Health Science Center at Houson, Children's Memorial Hermann Hospital, and Memorial Hermann Southwest Hospital | Houston, TX | Non-Author Contributor | Eunice Kennedy Shriver National Institute of Child Health and Human Development Neonatal Research Network |
| Carmen | Garcia | | RN CCRP | McGovern Medical School at The University of Texas Health Science Center at Houson, Children's Memorial Hermann Hospital, and Memorial Hermann Southwest Hospital | Houston, TX | Non-Author Contributor | Eunice Kennedy Shriver National Institute of Child Health and Human Development Neonatal Research Network |
| Donna J. | Hall | | RN | McGovern Medical School at The University of Texas Health Science Center at Houson, Children's Memorial Hermann Hospital, and Memorial Hermann Southwest Hospital | Houston, TX | Non-Author Contributor | Eunice Kennedy Shriver National Institute of Child Health and Human Development Neonatal Research Network |
| Margarita | Jiminez | | MD MPH | McGovern Medical School at The University of Texas Health Science Center at Houson, Children's Memorial Hermann Hospital, and Memorial Hermann Southwest Hospital | Houston, TX | Non-Author Contributor | Eunice Kennedy Shriver National Institute of Child Health and Human Development Neonatal Research Network |
| Janice | John | | CPNP | McGovern Medical School at The University of Texas Health Science Center at Houson, Children's Memorial Hermann Hospital, and Memorial Hermann Southwest Hospital | Houston, TX | Non-Author Contributor | Eunice Kennedy Shriver National Institute of Child Health and Human Development Neonatal Research Network |
| Patrick M. | Jones | | MD MA | McGovern Medical School at The University of Texas Health Science Center at Houson, Children's Memorial Hermann Hospital, and Memorial Hermann Southwest Hospital | Houston, TX | Non-Author Contributor | Eunice Kennedy Shriver National Institute of Child Health and Human Development Neonatal Research Network |
| M. Layne | Lillie | | RN BSN | McGovern Medical School at The University of Texas Health Science Center at Houson, Children's Memorial Hermann Hospital, and Memorial Hermann Southwest Hospital | Houston, TX | Non-Author Contributor | Eunice Kennedy Shriver National Institute of Child Health and Human Development Neonatal Research Network |
| Karen | Martin | | RN | McGovern Medical School at The University of Texas Health Science Center at Houson, Children's Memorial Hermann Hospital, and Memorial Hermann Southwest Hospital | Houston, TX | Non-Author Contributor | Eunice Kennedy Shriver National Institute of Child Health and Human Development Neonatal Research Network |
| Sara C. | Martin | | RN BSN | McGovern Medical School at The University of Texas Health Science Center at Houson, Children's Memorial Hermann Hospital, and Memorial Hermann Southwest Hospital | Houston, TX | Non-Author Contributor | Eunice Kennedy Shriver National Institute of Child Health and Human Development Neonatal Research Network |
| Georgia E. | McDavid | | RN | McGovern Medical School at The University of Texas Health Science Center at Houson, Children's Memorial Hermann Hospital, and Memorial Hermann Southwest Hospital | Houston, TX | Non-Author Contributor | Eunice Kennedy Shriver National Institute of Child Health and Human Development Neonatal Research Network |

| *First Name and Middle Initial(s) | *Last Name | *Suffix (eg, Jr, III) | Academic Degrees | Institution | Location (city, state/province, country) | Role or Contribution, eg, chair, principal investigator | Group (if more than 1 Group listed in the byline) and/or Subgroup (eg, Steering Committee) |
|---|---|---|---|---|---|---|---|
| Shannon L. | McKee | | EdS | McGovern Medical School at The University of Texas Health Science Center at Houson, Children's Memorial Hermann Hospital, and Memorial Hermann Southwest Hospital | Houston, TX | Non-Author Contributor | Eunice Kennedy Shriver National Institute of Child Health and Human Development Neonatal Research Network |
| Michelle | Poe | | PhD RN | McGovern Medical School at The University of Texas Health Science Center at Houson, Children's Memorial Hermann Hospital, and Memorial Hermann Southwest Hospital | Houston, TX | Non-Author Contributor | Eunice Kennedy Shriver National Institute of Child Health and Human Development Neonatal Research Network |
| Kimberly | Rennie | | PhD | McGovern Medical School at The University of Texas Health Science Center at Houson, Children's Memorial Hermann Hospital, and Memorial Hermann Southwest Hospital | Houston, TX | Non-Author Contributor | Eunice Kennedy Shriver National Institute of Child Health and Human Development Neonatal Research Network |
| Tina | Reddy | | MD | McGovern Medical School at The University of Texas Health Science Center at Houson, Children's Memorial Hermann Hospital, and Memorial Hermann Southwest Hospital | Houston, TX | Non-Author Contributor | Eunice Kennedy Shriver National Institute of Child Health and Human Development Neonatal Research Network |
| Shawna | Rodgers | | RN BSN | McGovern Medical School at The University of Texas Health Science Center at Houson, Children's Memorial Hermann Hospital, and Memorial Hermann Southwest Hospital | Houston, TX | Non-Author Contributor | Eunice Kennedy Shriver National Institute of Child Health and Human Development Neonatal Research Network |
| Saba | Khan Siddiki | | MD | McGovern Medical School at The University of Texas Health Science Center at Houson, Children's Memorial Hermann Hospital, and Memorial Hermann Southwest Hospital | Houston, TX | Non-Author Contributor | Eunice Kennedy Shriver National Institute of Child Health and Human Development Neonatal Research Network |
| Daniel | Sperry | | RN | McGovern Medical School at The University of Texas Health Science Center at Houson, Children's Memorial Hermann Hospital, and Memorial Hermann Southwest Hospital | Houston, TX | Non-Author Contributor | Eunice Kennedy Shriver National Institute of Child Health and Human Development Neonatal Research Network |
| Patti L. | Pierce Tate | | RCP | McGovern Medical School at The University of Texas Health Science Center at Houson, Children's Memorial Hermann Hospital, and Memorial Hermann Southwest Hospital | Houston, TX | Non-Author Contributor | Eunice Kennedy Shriver National Institute of Child Health and Human Development Neonatal Research Network |
| Sharon L. | Wright | | MT (ASCP) | McGovern Medical School at The University of Texas Health Science Center at Houson, Children's Memorial Hermann Hospital, and Memorial Hermann Southwest Hospital | Houston, TX | Non-Author Contributor | Eunice Kennedy Shriver National Institute of Child Health and Human Development Neonatal Research Network |
| Pablo J. | Sánchez | | MD | Nationwide Children's Hospital, The Abigal Wexner Research Institute at Nationwide Children's Hospital, Center for Perinatal Research, The Ohio State College of Medicine, The Ohio State University Wexner Medical Center, and Riverside Methodist Hospital | Columbus, OH | Non-Author Contributor | Eunice Kennedy Shriver National Institute of Child Health and Human Development Neonatal Research Network |
| Jonathan L. | Slaughter | | MD MPH | Nationwide Children's Hospital, The Abigal Wexner Research Institute at Nationwide Children's Hospital, Center for Perinatal Research, The Ohio State College of Medicine, The Ohio State University Wexner Medical Center, and Riverside Methodist Hospital | Columbus, OH | Non-Author Contributor | Eunice Kennedy Shriver National Institute of Child Health and Human Development Neonatal Research Network |
| Leif D. | Nelin | | MD | Nationwide Children's Hospital, The Abigal Wexner Research Institute at Nationwide Children's Hospital, Center for Perinatal Research, The Ohio State College of Medicine, The Ohio State University Wexner Medical Center, and Riverside Methodist Hospital | Columbus, OH | Non-Author Contributor | Eunice Kennedy Shriver National Institute of Child Health and Human Development Neonatal Research Network |
| Sudarshan R. | Jadcherla | | MD | Nationwide Children's Hospital, The Abigal Wexner Research Institute at Nationwide Children's Hospital, Center for Perinatal Research, The Ohio State College of Medicine, The Ohio State University Wexner Medical Center, and Riverside Methodist Hospital | Columbus, OH | Non-Author Contributor | Eunice Kennedy Shriver National Institute of Child Health and Human Development Neonatal Research Network |
| Nathalie L. | Maitre | | MD PhD | Nationwide Children's Hospital, The Abigal Wexner Research Institute at Nationwide Children's Hospital, Center for Perinatal Research, The Ohio State College of Medicine, The Ohio State University Wexner Medical Center, and Riverside Methodist Hospital | Columbus, OH | Non-Author Contributor | Eunice Kennedy Shriver National Institute of Child Health and Human Development Neonatal Research Network |
| Christopher | Timan | | MD | Nationwide Children's Hospital, The Abigal Wexner Research Institute at Nationwide Children's Hospital, Center for Perinatal Research, The Ohio State College of Medicine, The Ohio State University Wexner Medical Center, and Riverside Methodist Hospital | Columbus, OH | Non-Author Contributor | Eunice Kennedy Shriver National Institute of Child Health and Human Development Neonatal Research Network |

*Indicates required information. Only first name, last name, and suffix will appear in PubMed.

| *First Name and Middle Initial(s) | *Last Name | *Suffix (eg, Jr, III) | Academic Degrees | Institution | Location (city, state/province, country) | Role or Contribution, eg, chair, principal investigator | Group (if more than 1 Group listed in the byline) and/or Subgroup (eg, Steering Committee) |
|---|---|---|---|---|---|---|---|
| Keith O. | Yeates | | Md PhD | Nationwide Children's Hospital, The Abigal Wexner Research Institute at Nationwide Children's Hospital, Center for Perinatal Research, The Ohio State College of Medicine, The Ohio State University Wexner Medical Center, and Riverside Methodist Hospital | Columbus, OH | Non-Author Contributor | Eunice Kennedy Shriver National Institute of Child Health and Human Development Neonatal Research Network |
| Patricia | Luzader | | RN | Nationwide Children's Hospital, The Abigal Wexner Research Institute at Nationwide Children's Hospital, Center for Perinatal Research, The Ohio State College of Medicine, The Ohio State University Wexner Medical Center, and Riverside Methodist Hospital | Columbus, OH | Non-Author Contributor | Eunice Kennedy Shriver National Institute of Child Health and Human Development Neonatal Research Network |
| Nancy | Batterson | | OT/L | Nationwide Children's Hospital, The Abigal Wexner Research Institute at Nationwide Children's Hospital, Center for Perinatal Research, The Ohio State College of Medicine, The Ohio State University Wexner Medical Center, and Riverside Methodist Hospital | Columbus, OH | Non-Author Contributor | Eunice Kennedy Shriver National Institute of Child Health and Human Development Neonatal Research Network |
| Hallie | Baugher | | BS MSN | Nationwide Children's Hospital, The Abigal Wexner Research Institute at Nationwide Children's Hospital, Center for Perinatal Research, The Ohio State College of Medicine, The Ohio State University Wexner Medical Center, and Riverside Methodist Hospital | Columbus, OH | Non-Author Contributor | Eunice Kennedy Shriver National Institute of Child Health and Human Development Neonatal Research Network |
| Demi R. | Beckford | | MHS | Nationwide Children's Hospital, The Abigal Wexner Research Institute at Nationwide Children's Hospital, Center for Perinatal Research, The Ohio State College of Medicine, The Ohio State University Wexner Medical Center, and Riverside Methodist Hospital | Columbus, OH | Non-Author Contributor | Eunice Kennedy Shriver National Institute of Child Health and Human Development Neonatal Research Network |
| Stephanie | Burkhardt | | BS MPH | Nationwide Children's Hospital, The Abigal Wexner Research Institute at Nationwide Children's Hospital, Center for Perinatal Research, The Ohio State College of Medicine, The Ohio State University Wexner Medical Center, and Riverside Methodist Hospital | Columbus, OH | Non-Author Contributor | Eunice Kennedy Shriver National Institute of Child Health and Human Development Neonatal Research Network |
| Helen | Carey | | PT DHSc | Nationwide Children's Hospital, The Abigal Wexner Research Institute at Nationwide Children's Hospital, Center for Perinatal Research, The Ohio State College of Medicine, The Ohio State University Wexner Medical Center, and Riverside Methodist Hospital | Columbus, OH | Non-Author Contributor | Eunice Kennedy Shriver National Institute of Child Health and Human Development Neonatal Research Network |
| Michelle | Chao | | BS | Nationwide Children's Hospital, The Abigal Wexner Research Institute at Nationwide Children's Hospital, Center for Perinatal Research, The Ohio State College of Medicine, The Ohio State University Wexner Medical Center, and Riverside Methodist Hospital | Columbus, OH | Non-Author Contributor | Eunice Kennedy Shriver National Institute of Child Health and Human Development Neonatal Research Network |
| Courtney | Cira | | BS | Nationwide Children's Hospital, The Abigal Wexner Research Institute at Nationwide Children's Hospital, Center for Perinatal Research, The Ohio State College of Medicine, The Ohio State University Wexner Medical Center, and Riverside Methodist Hospital | Columbus, OH | Non-Author Contributor | Eunice Kennedy Shriver National Institute of Child Health and Human Development Neonatal Research Network |
| Erna | Clark | | BA | Nationwide Children's Hospital, The Abigal Wexner Research Institute at Nationwide Children's Hospital, Center for Perinatal Research, The Ohio State College of Medicine, The Ohio State University Wexner Medical Center, and Riverside Methodist Hospital | Columbus, OH | Non-Author Contributor | Eunice Kennedy Shriver National Institute of Child Health and Human Development Neonatal Research Network |
| Brittany | DeSantis | | BS | Nationwide Children's Hospital, The Abigal Wexner Research Institute at Nationwide Children's Hospital, Center for Perinatal Research, The Ohio State College of Medicine, The Ohio State University Wexner Medical Center, and Riverside Methodist Hospital | Columbus, OH | Non-Author Contributor | Eunice Kennedy Shriver National Institute of Child Health and Human Development Neonatal Research Network |
| Christine A. | Fortney | | RN PhD | Nationwide Children's Hospital, The Abigal Wexner Research Institute at Nationwide Children's Hospital, Center for Perinatal Research, The Ohio State College of Medicine, The Ohio State University Wexner Medical Center, and Riverside Methodist Hospital | Columbus, OH | Non-Author Contributor | Eunice Kennedy Shriver National Institute of Child Health and Human Development Neonatal Research Network |
| Aubrey | Fowler | | BS | Nationwide Children's Hospital, The Abigal Wexner Research Institute at Nationwide Children's Hospital, Center for Perinatal Research, The Ohio State College of Medicine, The Ohio State University Wexner Medical Center, and Riverside Methodist Hospital | Columbus, OH | Non-Author Contributor | Eunice Kennedy Shriver National Institute of Child Health and Human Development Neonatal Research Network |
| Julie | Gutentag | | RN BSN | Nationwide Children's Hospital, The Abigal Wexner Research Institute at Nationwide Children's Hospital, Center for Perinatal Research, The Ohio State College of Medicine, The Ohio State University Wexner Medical Center, and Riverside Methodist Hospital | Columbus, OH | Non-Author Contributor | Eunice Kennedy Shriver National Institute of Child Health and Human Development Neonatal Research Network |
| Jennifer L. | Grothause | | BA RN BSN | Nationwide Children's Hospital, The Abigal Wexner Research Institute at Nationwide Children's Hospital, Center for Perinatal Research, The Ohio State College of Medicine, The Ohio State University Wexner Medical Center, and Riverside Methodist Hospital | Columbus, OH | Non-Author Contributor | Eunice Kennedy Shriver National Institute of Child Health and Human Development Neonatal Research Network |

| *First Name and Middle Initial(s) | *Last Name | *Suffix (eg, Jr, III) | Academic Degrees | Institution | Location (city, state/province, country) | Role or Contribution, eg, chair, principal investigator | Group (if more than 1 Group listed in the byline) and/or Subgroup (eg, Steering Committee) |
|---|---|---|---|---|---|---|---|
| Cole D. | Hague | | BA MS | Nationwide Children's Hospital, The Abigal Wexner Research Institute at Nationwide Children's Hospital, Center for Perinatal Research, The Ohio State College of Medicine, The Ohio State University Wexner Medical Center, and Riverside Methodist Hospital | Columbus, OH | Non-Author Contributor | Eunice Kennedy Shriver National Institute of Child Health and Human Development Neonatal Research Network |
| Sarah A. | Keim | | PhD MA MS | Nationwide Children's Hospital, The Abigal Wexner Research Institute at Nationwide Children's Hospital, Center for Perinatal Research, The Ohio State College of Medicine, The Ohio State University Wexner Medical Center, and Riverside Methodist Hospital | Columbus, OH | Non-Author Contributor | Eunice Kennedy Shriver National Institute of Child Health and Human Development Neonatal Research Network |
| Katelyn | Levengood | | PT DPT | Nationwide Children's Hospital, The Abigal Wexner Research Institute at Nationwide Children's Hospital, Center for Perinatal Research, The Ohio State College of Medicine, The Ohio State University Wexner Medical Center, and Riverside Methodist Hospital | Columbus, OH | Non-Author Contributor | Eunice Kennedy Shriver National Institute of Child Health and Human Development Neonatal Research Network |
| Laura | Marzec | | MD | Nationwide Children's Hospital, The Abigal Wexner Research Institute at Nationwide Children's Hospital, Center for Perinatal Research, The Ohio State College of Medicine, The Ohio State University Wexner Medical Center, and Riverside Methodist Hospital | Columbus, OH | Non-Author Contributor | Eunice Kennedy Shriver National Institute of Child Health and Human Development Neonatal Research Network |
| Jacqueline | McCool | | | Nationwide Children's Hospital, The Abigal Wexner Research Institute at Nationwide Children's Hospital, Center for Perinatal Research, The Ohio State College of Medicine, The Ohio State University Wexner Medical Center, and Riverside Methodist Hospital | Columbus, OH | Non-Author Contributor | Eunice Kennedy Shriver National Institute of Child Health and Human Development Neonatal Research Network |
| Bethany | Miller | | RN BSN | Nationwide Children's Hospital, The Abigal Wexner Research Institute at Nationwide Children's Hospital, Center for Perinatal Research, The Ohio State College of Medicine, The Ohio State University Wexner Medical Center, and Riverside Methodist Hospital | Columbus, OH | Non-Author Contributor | Eunice Kennedy Shriver National Institute of Child Health and Human Development Neonatal Research Network |
| Mary Ann | Nelin | | MD | Nationwide Children's Hospital, The Abigal Wexner Research Institute at Nationwide Children's Hospital, Center for Perinatal Research, The Ohio State College of Medicine, The Ohio State University Wexner Medical Center, and Riverside Methodist Hospital | Columbus, OH | Non-Author Contributor | Eunice Kennedy Shriver National Institute of Child Health and Human Development Neonatal Research Network |
| Julia | Newton | | MPH | Nationwide Children's Hospital, The Abigal Wexner Research Institute at Nationwide Children's Hospital, Center for Perinatal Research, The Ohio State College of Medicine, The Ohio State University Wexner Medical Center, and Riverside Methodist Hospital | Columbus, OH | Non-Author Contributor | Eunice Kennedy Shriver National Institute of Child Health and Human Development Neonatal Research Network |
| Courtney | Park | | RN BSN | Nationwide Children's Hospital, The Abigal Wexner Research Institute at Nationwide Children's Hospital, Center for Perinatal Research, The Ohio State College of Medicine, The Ohio State University Wexner Medical Center, and Riverside Methodist Hospital | Columbus, OH | Non-Author Contributor | Eunice Kennedy Shriver National Institute of Child Health and Human Development Neonatal Research Network |
| Lindsay | Pietruszewski | | PT DPT | Nationwide Children's Hospital, The Abigal Wexner Research Institute at Nationwide Children's Hospital, Center for Perinatal Research, The Ohio State College of Medicine, The Ohio State University Wexner Medical Center, and Riverside Methodist Hospital | Columbus, OH | Non-Author Contributor | Eunice Kennedy Shriver National Institute of Child Health and Human Development Neonatal Research Network |
| Jessica | Purnell | | BS CCRC | Nationwide Children's Hospital, The Abigal Wexner Research Institute at Nationwide Children's Hospital, Center for Perinatal Research, The Ohio State College of Medicine, The Ohio State University Wexner Medical Center, and Riverside Methodist Hospital | Columbus, OH | Non-Author Contributor | Eunice Kennedy Shriver National Institute of Child Health and Human Development Neonatal Research Network |
| Ruth | Seabrook | | MD | Nationwide Children's Hospital, The Abigal Wexner Research Institute at Nationwide Children's Hospital, Center for Perinatal Research, The Ohio State College of Medicine, The Ohio State University Wexner Medical Center, and Riverside Methodist Hospital | Columbus, OH | Non-Author Contributor | Eunice Kennedy Shriver National Institute of Child Health and Human Development Neonatal Research Network |
| Julie C. | Shadd | | BSN RD | Nationwide Children's Hospital, The Abigal Wexner Research Institute at Nationwide Children's Hospital, Center for Perinatal Research, The Ohio State College of Medicine, The Ohio State University Wexner Medical Center, and Riverside Methodist Hospital | Columbus, OH | Non-Author Contributor | Eunice Kennedy Shriver National Institute of Child Health and Human Development Neonatal Research Network |
| Kristi | Small | | BS | Nationwide Children's Hospital, The Abigal Wexner Research Institute at Nationwide Children's Hospital, Center for Perinatal Research, The Ohio State College of Medicine, The Ohio State University Wexner Medical Center, and Riverside Methodist Hospital | Columbus, OH | Non-Author Contributor | Eunice Kennedy Shriver National Institute of Child Health and Human Development Neonatal Research Network |
| Melanie | Stein | | RRT BBS | Nationwide Children's Hospital, The Abigal Wexner Research Institute at Nationwide Children's Hospital, Center for Perinatal Research, The Ohio State College of Medicine, The Ohio State University Wexner Medical Center, and Riverside Methodist Hospital | Columbus, OH | Non-Author Contributor | Eunice Kennedy Shriver National Institute of Child Health and Human Development Neonatal Research Network |

*Indicates required information. Only first name, last name, and suffix will appear in PubMed.

| *First Name and Middle Initial(s) | *Last Name | *Suffix (eg, Jr, III) | Academic Degrees | Institution | Location (city, state/province, country) | Role or Contribution, eg, chair, principal investigator | Group (if more than 1 Group listed in the byline) and/or Subgroup (eg, Steering Committee) |
|---|---|---|---|---|---|---|---|
| Margaret | Sullivan | | BS | Nationwide Children's Hospital, The Abigal Wexner Research Institute at Nationwide Children's Hospital, Center for Perinatal Research, The Ohio State College of Medicine, The Ohio State University Wexner Medical Center, and Riverside Methodist Hospital | Columbus, OH | Non-Author Contributor | Eunice Kennedy Shriver National Institute of Child Health and Human Development Neonatal Research Network |
| Rox Ann | Sullivan | | RN BSN | Nationwide Children's Hospital, The Abigal Wexner Research Institute at Nationwide Children's Hospital, Center for Perinatal Research, The Ohio State College of Medicine, The Ohio State University Wexner Medical Center, and Riverside Methodist Hospital | Columbus, OH | Non-Author Contributor | Eunice Kennedy Shriver National Institute of Child Health and Human Development Neonatal Research Network |
| Krystin | Warnimont | | BS | Nationwide Children's Hospital, The Abigal Wexner Research Institute at Nationwide Children's Hospital, Center for Perinatal Research, The Ohio State College of Medicine, The Ohio State University Wexner Medical Center, and Riverside Methodist Hospital | Columbus, OH | Non-Author Contributor | Eunice Kennedy Shriver National Institute of Child Health and Human Development Neonatal Research Network |
| Lina | Yossef-Salameh | | MD | Nationwide Children's Hospital, The Abigal Wexner Research Institute at Nationwide Children's Hospital, Center for Perinatal Research, The Ohio State College of Medicine, The Ohio State University Wexner Medical Center, and Riverside Methodist Hospital | Columbus, OH | Non-Author Contributor | Eunice Kennedy Shriver National Institute of Child Health and Human Development Neonatal Research Network |
| Courtney | Cira | | BS | Nationwide Children's Hospital, The Abigal Wexner Research Institute at Nationwide Children's Hospital, Center for Perinatal Research, The Ohio State College of Medicine, The Ohio State University Wexner Medical Center, and Riverside Methodist Hospital | Columbus, OH | Non-Author Contributor | Eunice Kennedy Shriver National Institute of Child Health and Human Development Neonatal Research Network |
| Erin | Fearns | | | Nationwide Children's Hospital, The Abigal Wexner Research Institute at Nationwide Children's Hospital, Center for Perinatal Research, The Ohio State College of Medicine, The Ohio State University Wexner Medical Center, and Riverside Methodist Hospital | Columbus, OH | Non-Author Contributor | Eunice Kennedy Shriver National Institute of Child Health and Human Development Neonatal Research Network |
| Abhik | Das | | PhD | RTI International | Research Triangle Park, North Carolina | Non-Author Contributor | Eunice Kennedy Shriver National Institute of Child Health and Human Development Neonatal Research Network |
| Marie G. | Gantz | | PhD | RTI International | Research Triangle Park, North Carolina | Non-Author Contributor | Eunice Kennedy Shriver National Institute of Child Health and Human Development Neonatal Research Network |
| Carla M. | Bann | | PhD | RTI International | Research Triangle Park, North Carolina | Non-Author Contributor | Eunice Kennedy Shriver National Institute of Child Health and Human Development Neonatal Research Network |
| Dennis | Wallace | | PhD | RTI International | Research Triangle Park, North Carolina | Non-Author Contributor | Eunice Kennedy Shriver National Institute of Child Health and Human Development Neonatal Research Network |
| Jeanette | O'Donnell Auman | | BS | RTI International | Research Triangle Park, North Carolina | Non-Author Contributor | Eunice Kennedy Shriver National Institute of Child Health and Human Development Neonatal Research Network |
| Margaret | Crawford | | BS | RTI International | Research Triangle Park, North Carolina | Non-Author Contributor | Eunice Kennedy Shriver National Institute of Child Health and Human Development Neonatal Research Network |
| Jenna | Gabrio | | BS MPH | RTI International | Research Triangle Park, North Carolina | Non-Author Contributor | Eunice Kennedy Shriver National Institute of Child Health and Human Development Neonatal Research Network |
| Jamie E. | Newman | | PhD MPH | RTI International | Research Triangle Park, North Carolina | Non-Author Contributor | Eunice Kennedy Shriver National Institute of Child Health and Human Development Neonatal Research Network |
| Lindsay | Parlberg | | BS | RTI International | Research Triangle Park, North Carolina | Non-Author Contributor | Eunice Kennedy Shriver National Institute of Child Health and Human Development Neonatal Research Network |

| *First Name and Middle Initial(s) | *Last Name | *Suffix (eg, Jr, III) | Academic Degrees | Institution | Location (city, state/province, country) | Role or Contribution, eg, chair, principal investigator | Group (if more than 1 Group listed in the byline) and/or Subgroup (eg, Steering Committee) |
|---|---|---|---|---|---|---|---|
| Carolyn M. | Petrie Huitema | | MS | RTI International | Research Triangle Park, North Carolina | Non-Author Contributor | Eunice Kennedy Shriver National Institute of Child Health and Human Development Neonatal Research Network |
| Kristin M. | Zaterka-Baxter | | RN BSN | RTI International | Research Triangle Park, North Carolina | Non-Author Contributor | Eunice Kennedy Shriver National Institute of Child Health and Human Development Neonatal Research Network |
| Krisa P. | Van Meurs | | MD | Stanford University, El Camino Hospital, and Lucile Packard Children's Hospital | Stanford, Mountain View and Palo Alto, CA | Non-Author Contributor | Eunice Kennedy Shriver National Institute of Child Health and Human Development Neonatal Research Network |
| Valerie Y. | Chock | | MD MS Epi | Stanford University, El Camino Hospital, and Lucile Packard Children's Hospital | Stanford, Mountain View and Palo Alto, CA | Non-Author Contributor | Eunice Kennedy Shriver National Institute of Child Health and Human Development Neonatal Research Network |
| David K. | Stevenson | | MD | Stanford University, El Camino Hospital, and Lucile Packard Children's Hospital | Stanford, Mountain View and Palo Alto, CA | Non-Author Contributor | Eunice Kennedy Shriver National Institute of Child Health and Human Development Neonatal Research Network |
| Susan R. | Hintz | | MD MS Epi | Stanford University, El Camino Hospital, and Lucile Packard Children's Hospital | Stanford, Mountain View and Palo Alto, CA | Non-Author Contributor | Eunice Kennedy Shriver National Institute of Child Health and Human Development Neonatal Research Network |
| Bethany M. | Ball | | CCRC | Stanford University, El Camino Hospital, and Lucile Packard Children's Hospital | Stanford, Mountain View and Palo Alto, CA | Non-Author Contributor | Eunice Kennedy Shriver National Institute of Child Health and Human Development Neonatal Research Network |
| Marian M. | Adams | | MD | Stanford University, El Camino Hospital, and Lucile Packard Children's Hospital | Stanford, Mountain View and Palo Alto, CA | Non-Author Contributor | Eunice Kennedy Shriver National Institute of Child Health and Human Development Neonatal Research Network |
| Dona | Bahmani | | CRC | Stanford University, El Camino Hospital, and Lucile Packard Children's Hospital | Stanford, Mountain View and Palo Alto, CA | Non-Author Contributor | Eunice Kennedy Shriver National Institute of Child Health and Human Development Neonatal Research Network |
| Barbara | Bentley | | PsychD MSEd | Stanford University, El Camino Hospital, and Lucile Packard Children's Hospital | Stanford, Mountain View and Palo Alto, CA | Non-Author Contributor | Eunice Kennedy Shriver National Institute of Child Health and Human Development Neonatal Research Network |
| Maria Elena | DeAnda | | PhD | Stanford University, El Camino Hospital, and Lucile Packard Children's Hospital | Stanford, Mountain View and Palo Alto, CA | Non-Author Contributor | Eunice Kennedy Shriver National Institute of Child Health and Human Development Neonatal Research Network |
| Anne M. | DeBattista | | RN PNP PhD | Stanford University, El Camino Hospital, and Lucile Packard Children's Hospital | Stanford, Mountain View and Palo Alto, CA | Non-Author Contributor | Eunice Kennedy Shriver National Institute of Child Health and Human Development Neonatal Research Network |
| Beth | Earhart | | PhD | Stanford University, El Camino Hospital, and Lucile Packard Children's Hospital | Stanford, Mountain View and Palo Alto, CA | Non-Author Contributor | Eunice Kennedy Shriver National Institute of Child Health and Human Development Neonatal Research Network |
| Lynne C. | Huffman | | MD | Stanford University, El Camino Hospital, and Lucile Packard Children's Hospital | Stanford, Mountain View and Palo Alto, CA | Non-Author Contributor | Eunice Kennedy Shriver National Institute of Child Health and Human Development Neonatal Research Network |
| Casey E. | Krueger | | PhD | Stanford University, El Camino Hospital, and Lucile Packard Children's Hospital | Stanford, Mountain View and Palo Alto, CA | Non-Author Contributor | Eunice Kennedy Shriver National Institute of Child Health and Human Development Neonatal Research Network |

| *First Name and Middle Initial(s) | *Last Name | *Suffix (eg, Jr, III) | Academic Degrees | Institution | Location (city, state/province, country) | Role or Contribution, eg, chair, principal investigator | Group (if more than 1 Group listed in the byline) and/or Subgroup (eg, Steering Committee) |
|---|---|---|---|---|---|---|---|
| Ryan E. | Lucash | | PhD | Stanford University, El Camino Hospital, and Lucile Packard Children's Hospital | Stanford, Mountain View and Palo Alto, CA | Non-Author Contributor | Eunice Kennedy Shriver National Institute of Child Health and Human Development Neonatal Research Network |
| Melinda S. | Proud | | RCP | Stanford University, El Camino Hospital, and Lucile Packard Children's Hospital | Stanford, Mountain View and Palo Alto, CA | Non-Author Contributor | Eunice Kennedy Shriver National Institute of Child Health and Human Development Neonatal Research Network |
| Elizabeth N. | Reichert | | MA CCRC | Stanford University, El Camino Hospital, and Lucile Packard Children's Hospital | Stanford, Mountain View and Palo Alto, CA | Non-Author Contributor | Eunice Kennedy Shriver National Institute of Child Health and Human Development Neonatal Research Network |
| Heather | Taylor | | PhD | Stanford University, El Camino Hospital, and Lucile Packard Children's Hospital | Stanford, Mountain View and Palo Alto, CA | Non-Author Contributor | Eunice Kennedy Shriver National Institute of Child Health and Human Development Neonatal Research Network |
| Hali E. | Weiss | | MD | Stanford University, El Camino Hospital, and Lucile Packard Children's Hospital | Stanford, Mountain View and Palo Alto, CA | Non-Author Contributor | Eunice Kennedy Shriver National Institute of Child Health and Human Development Neonatal Research Network |
| Jordan R. | Williams | | MD | Stanford University, El Camino Hospital, and Lucile Packard Children's Hospital | Stanford, Mountain View and Palo Alto, CA | Non-Author Contributor | Eunice Kennedy Shriver National Institute of Child Health and Human Development Neonatal Research Network |
| Waldemar A. | Carlo | | MD | University of Alabama at Birmingham Health System and Children's Hospital of Alabama | Birmingham, AL | Non-Author Contributor | Eunice Kennedy Shriver National Institute of Child Health and Human Development Neonatal Research Network |
| Namasivayam | Ambalavanan | | MD | University of Alabama at Birmingham Health System and Children's Hospital of Alabama | Birmingham, AL | Non-Author Contributor | Eunice Kennedy Shriver National Institute of Child Health and Human Development Neonatal Research Network |
| Myriam | Peralta-Carcelen | | MD MPH | University of Alabama at Birmingham Health System and Children's Hospital of Alabama | Birmingham, AL | Non-Author Contributor | Eunice Kennedy Shriver National Institute of Child Health and Human Development Neonatal Research Network |
| Monica V. | Collins | | RN BSN MaEd | University of Alabama at Birmingham Health System and Children's Hospital of Alabama | Birmingham, AL | Non-Author Contributor | Eunice Kennedy Shriver National Institute of Child Health and Human Development Neonatal Research Network |
| Shirley S. | Cosby | | RN BSN | University of Alabama at Birmingham Health System and Children's Hospital of Alabama | Birmingham, AL | Non-Author Contributor | Eunice Kennedy Shriver National Institute of Child Health and Human Development Neonatal Research Network |
| Kirstin J. | Bailey | | PhD | University of Alabama at Birmingham Health System and Children's Hospital of Alabama | Birmingham, AL | Non-Author Contributor | Eunice Kennedy Shriver National Institute of Child Health and Human Development Neonatal Research Network |
| Fred J. | Biasini | | PhD | University of Alabama at Birmingham Health System and Children's Hospital of Alabama | Birmingham, AL | Non-Author Contributor | Eunice Kennedy Shriver National Institute of Child Health and Human Development Neonatal Research Network |
| Stephanie A. | Chopko | | PhD | University of Alabama at Birmingham Health System and Children's Hospital of Alabama | Birmingham, AL | Non-Author Contributor | Eunice Kennedy Shriver National Institute of Child Health and Human Development Neonatal Research Network |
| Kristy A. | Domnanovich | | PhD | University of Alabama at Birmingham Health System and Children's Hospital of Alabama | Birmingham, AL | Non-Author Contributor | Eunice Kennedy Shriver National Institute of Child Health and Human Development Neonatal Research Network |

| *First Name and Middle Initial(s) | *Last Name | *Suffix (eg, Jr, III) | Academic Degrees | Institution | Location (city, state/province, country) | Role or Contribution, eg, chair, principal investigator | Group (if more than 1 Group listed in the byline) and/or Subgroup (eg, Steering Committee) |
|---|---|---|---|---|---|---|---|
| Chantel J. | Jno-Finn | | PT DPT | University of Alabama at Birmingham Health System and Children's Hospital of Alabama | Birmingham, AL | Non-Author Contributor | Eunice Kennedy Shriver National Institute of Child Health and Human Development Neonatal Research Network |
| Morissa | Ladinsky | | MD | University of Alabama at Birmingham Health System and Children's Hospital of Alabama | Birmingham, AL | Non-Author Contributor | Eunice Kennedy Shriver National Institute of Child Health and Human Development Neonatal Research Network |
| Mary Beth | Moses | | PT MS PCS | University of Alabama at Birmingham Health System and Children's Hospital of Alabama | Birmingham, AL | Non-Author Contributor | Eunice Kennedy Shriver National Institute of Child Health and Human Development Neonatal Research Network |
| Tara E. | McNair | | RN BSN | University of Alabama at Birmingham Health System and Children's Hospital of Alabama | Birmingham, AL | Non-Author Contributor | Eunice Kennedy Shriver National Institute of Child Health and Human Development Neonatal Research Network |
| Vivien A. | Phillips | | RN BSN | University of Alabama at Birmingham Health System and Children's Hospital of Alabama | Birmingham, AL | Non-Author Contributor | Eunice Kennedy Shriver National Institute of Child Health and Human Development Neonatal Research Network |
| Julie | Preskitt | | MSOT MPH | University of Alabama at Birmingham Health System and Children's Hospital of Alabama | Birmingham, AL | Non-Author Contributor | Eunice Kennedy Shriver National Institute of Child Health and Human Development Neonatal Research Network |
| Richard V. | Rector | | PhD | University of Alabama at Birmingham Health System and Children's Hospital of Alabama | Birmingham, AL | Non-Author Contributor | Eunice Kennedy Shriver National Institute of Child Health and Human Development Neonatal Research Network |
| Kimberlly | Stringer | | MD MPH | University of Alabama at Birmingham Health System and Children's Hospital of Alabama | Birmingham, AL | Non-Author Contributor | Eunice Kennedy Shriver National Institute of Child Health and Human Development Neonatal Research Network |
| Sally | Whitley | | MA OTR-L FAOTA | University of Alabama at Birmingham Health System and Children's Hospital of Alabama | Birmingham, AL | Non-Author Contributor | Eunice Kennedy Shriver National Institute of Child Health and Human Development Neonatal Research Network |
| Sheree | York Chapman | | PT DPT PCS | University of Alabama at Birmingham Health System and Children's Hospital of Alabama | Birmingham, AL | Non-Author Contributor | Eunice Kennedy Shriver National Institute of Child Health and Human Development Neonatal Research Network |
| Uday | Devaskar | | MD | University of California - Los Angeles, Mattel Children's Hospital, Santa Monica Hospital, Los Robles Hospital and Medical Center, and Olive View Medical Center | Los Angeles, Santa Monica, Thousand Oaks, and Sylmar, CA | Non-Author Contributor | Eunice Kennedy Shriver National Institute of Child Health and Human Development Neonatal Research Network |
| Meena | Garg | | MD | University of California - Los Angeles, Mattel Children's Hospital, Santa Monica Hospital, Los Robles Hospital and Medical Center, and Olive View Medical Center | Los Angeles, Santa Monica, Thousand Oaks, and Sylmar, CA | Non-Author Contributor | Eunice Kennedy Shriver National Institute of Child Health and Human Development Neonatal Research Network |
| Isabell B. | Purdy | | PhD CPNP | University of California - Los Angeles, Mattel Children's Hospital, Santa Monica Hospital, Los Robles Hospital and Medical Center, and Olive View Medical Center | Los Angeles, Santa Monica, Thousand Oaks, and Sylmar, CA | Non-Author Contributor | Eunice Kennedy Shriver National Institute of Child Health and Human Development Neonatal Research Network |
| Teresa | Chanlaw | | MPH | University of California - Los Angeles, Mattel Children's Hospital, Santa Monica Hospital, Los Robles Hospital and Medical Center, and Olive View Medical Center | Los Angeles, Santa Monica, Thousand Oaks, and Sylmar, CA | Non-Author Contributor | Eunice Kennedy Shriver National Institute of Child Health and Human Development Neonatal Research Network |
| Rachel | Geller | | RN BSN | University of California - Los Angeles, Mattel Children's Hospital, Santa Monica Hospital, Los Robles Hospital and Medical Center, and Olive View Medical Center | Los Angeles, Santa Monica, Thousand Oaks, and Sylmar, CA | Non-Author Contributor | Eunice Kennedy Shriver National Institute of Child Health and Human Development Neonatal Research Network |

*Indicates required information. Only first name, last name, and suffix will appear in PubMed.

| ***First Name and Middle Initial(s)** | ***Last Name** | ***Suffix (eg, Jr, III)** | Academic Degrees | Institution | Location (city, state/province, country) | Role or Contribution, eg, chair, principal investigator | Group (if more than 1 Group listed in the byline) and/or Subgroup (eg, Steering Committee) |
|---|---|---|---|---|---|---|---|
| Tarah T. | Colaizy | | MD MPH | University of Iowa, Mercy Medical Center, and Sanford Health | Iowa City and Sheldon, IA | Non-Author Contributor | Eunice Kennedy Shriver National Institute of Child Health and Human Development Neonatal Research Network |
| John A. | Widness | | MD | University of Iowa, Mercy Medical Center, and Sanford Health | Iowa City and Sheldon, IA | Non-Author Contributor | Eunice Kennedy Shriver National Institute of Child Health and Human Development Neonatal Research Network |
| Jane E. | Brumbaugh | | MD | University of Iowa, Mercy Medical Center, and Sanford Health | Iowa City and Sheldon, IA | Non-Author Contributor | Eunice Kennedy Shriver National Institute of Child Health and Human Development Neonatal Research Network |
| Heidi M. | Harmon | | MD | University of Iowa, Mercy Medical Center, and Sanford Health | Iowa City and Sheldon, IA | Non-Author Contributor | Eunice Kennedy Shriver National Institute of Child Health and Human Development Neonatal Research Network |
| Karen J. | Johnson | | RN BSN | University of Iowa, Mercy Medical Center, and Sanford Health | Iowa City and Sheldon, IA | Non-Author Contributor | Eunice Kennedy Shriver National Institute of Child Health and Human Development Neonatal Research Network |
| Mendi L. | Schmelzel | | RN MSN | University of Iowa, Mercy Medical Center, and Sanford Health | Iowa City and Sheldon, IA | Non-Author Contributor | Eunice Kennedy Shriver National Institute of Child Health and Human Development Neonatal Research Network |
| Jacky R. | Walker | | RN | University of Iowa, Mercy Medical Center, and Sanford Health | Iowa City and Sheldon, IA | Non-Author Contributor | Eunice Kennedy Shriver National Institute of Child Health and Human Development Neonatal Research Network |
| Claire A. | Goeke | | RN | University of Iowa, Mercy Medical Center, and Sanford Health | Iowa City and Sheldon, IA | Non-Author Contributor | Eunice Kennedy Shriver National Institute of Child Health and Human Development Neonatal Research Network |
| Diane L. | Eastman | | RN CPNP MA | University of Iowa, Mercy Medical Center, and Sanford Health | Iowa City and Sheldon, IA | Non-Author Contributor | Eunice Kennedy Shriver National Institute of Child Health and Human Development Neonatal Research Network |
| Michelle L. | Baack | | MD | University of Iowa, Mercy Medical Center, and Sanford Health | Iowa City and Sheldon, IA | Non-Author Contributor | Eunice Kennedy Shriver National Institute of Child Health and Human Development Neonatal Research Network |
| Laurie A. | Hogden | | MD | University of Iowa, Mercy Medical Center, and Sanford Health | Iowa City and Sheldon, IA | Non-Author Contributor | Eunice Kennedy Shriver National Institute of Child Health and Human Development Neonatal Research Network |
| Megan M. | Henning | | RN | University of Iowa, Mercy Medical Center, and Sanford Health | Iowa City and Sheldon, IA | Non-Author Contributor | Eunice Kennedy Shriver National Institute of Child Health and Human Development Neonatal Research Network |
| Chelsey | Elenkiwich | | RN BSN | University of Iowa, Mercy Medical Center, and Sanford Health | Iowa City and Sheldon, IA | Non-Author Contributor | Eunice Kennedy Shriver National Institute of Child Health and Human Development Neonatal Research Network |
| Megan | Broadbent | | RN BSN | University of Iowa, Mercy Medical Center, and Sanford Health | Iowa City and Sheldon, IA | Non-Author Contributor | Eunice Kennedy Shriver National Institute of Child Health and Human Development Neonatal Research Network |
| Dan L. | Ellsbury | | MD | University of Iowa, Mercy Medical Center, and Sanford Health | Iowa City and Sheldon, IA | Non-Author Contributor | Eunice Kennedy Shriver National Institute of Child Health and Human Development Neonatal Research Network |

*Indicates required information. Only first name, last name, and suffix will appear in PubMed.

| *First Name and Middle Initial(s) | *Last Name | *Suffix (eg, Jr, III) | Academic Degrees | Institution | Location (city, state/province, country) | Role or Contribution, eg, chair, principal investigator | Group (if more than 1 Group listed in the byline) and/or Subgroup (eg, Steering Committee) |
|---|---|---|---|---|---|---|---|
| Donia B. | Bass | | RNC-NIC | University of Iowa, Mercy Medical Center, and Sanford Health | Iowa City and Sheldon, IA | Non-Author Contributor | Eunice Kennedy Shriver National Institute of Child Health and Human Development Neonatal Research Network |
| Tracy L. | Tud | | RN | University of Iowa, Mercy Medical Center, and Sanford Health | Iowa City and Sheldon, IA | Non-Author Contributor | Eunice Kennedy Shriver National Institute of Child Health and Human Development Neonatal Research Network |
| Janell | Fuller | | MD | University of New Mexico Health Sciences Center | Albuquerque, NM | Non-Author Contributor | Eunice Kennedy Shriver National Institute of Child Health and Human Development Neonatal Research Network |
| Robin K. | Ohls | | MD | University of New Mexico Health Sciences Center | Albuquerque, NM | Non-Author Contributor | Eunice Kennedy Shriver National Institute of Child Health and Human Development Neonatal Research Network |
| Conra | Backstrom Lacy | | RN | University of New Mexico Health Sciences Center | Albuquerque, NM | Non-Author Contributor | Eunice Kennedy Shriver National Institute of Child Health and Human Development Neonatal Research Network |
| Carol | Hartenberger | | BSN MPH | University of New Mexico Health Sciences Center | Albuquerque, NM | Non-Author Contributor | Eunice Kennedy Shriver National Institute of Child Health and Human Development Neonatal Research Network |
| Sandra | Sundquist Beauman | | MSN RNC | University of New Mexico Health Sciences Center | Albuquerque, NM | Non-Author Contributor | Eunice Kennedy Shriver National Institute of Child Health and Human Development Neonatal Research Network |
| Mary | Hanson | | RN BSN | University of New Mexico Health Sciences Center | Albuquerque, NM | Non-Author Contributor | Eunice Kennedy Shriver National Institute of Child Health and Human Development Neonatal Research Network |
| Jean R. | Lowe | | PhD | University of New Mexico Health Sciences Center | Albuquerque, NM | Non-Author Contributor | Eunice Kennedy Shriver National Institute of Child Health and Human Development Neonatal Research Network |
| Elizabeth | Kuan | | RN BSN | University of New Mexico Health Sciences Center | Albuquerque, NM | Non-Author Contributor | Eunice Kennedy Shriver National Institute of Child Health and Human Development Neonatal Research Network |
| Barbara | Schmidt | | MD MSc | University of Pennsylvania, Hospital of the University of Pennsylvania, Pennsylvania Hospital, Children's Hospital of Philadelphia, and Virtua Voorhees Hospital | Philadelphia, PA and Voorhees, NJ | Non-Author Contributor | Eunice Kennedy Shriver National Institute of Child Health and Human Development Neonatal Research Network |
| Haresh | Kirpalani | | MB MSc | University of Pennsylvania, Hospital of the University of Pennsylvania, Pennsylvania Hospital, Children's Hospital of Philadelphia, and Virtua Voorhees Hospital | Philadelphia, PA and Voorhees, NJ | Non-Author Contributor | Eunice Kennedy Shriver National Institute of Child Health and Human Development Neonatal Research Network |
| Soraya | Abbasi | | MD | University of Pennsylvania, Hospital of the University of Pennsylvania, Pennsylvania Hospital, Children's Hospital of Philadelphia, and Virtua Voorhees Hospital | Philadelphia, PA and Voorhees, NJ | Non-Author Contributor | Eunice Kennedy Shriver National Institute of Child Health and Human Development Neonatal Research Network |
| Aasma S. | Chaudhary | | BS RRT | University of Pennsylvania, Hospital of the University of Pennsylvania, Pennsylvania Hospital, Children's Hospital of Philadelphia, and Virtua Voorhees Hospital | Philadelphia, PA and Voorhees, NJ | Non-Author Contributor | Eunice Kennedy Shriver National Institute of Child Health and Human Development Neonatal Research Network |
| Toni | Mancini | | RN BSN CCRC | University of Pennsylvania, Hospital of the University of Pennsylvania, Pennsylvania Hospital, Children's Hospital of Philadelphia, and Virtua Voorhees Hospital | Philadelphia, PA and Voorhees, NJ | Non-Author Contributor | Eunice Kennedy Shriver National Institute of Child Health and Human Development Neonatal Research Network |

| ***First Name and Middle Initial(s)** | ***Last Name** | ***Suffix (eg, Jr, III)** | Academic Degrees | Institution | Location (city, state/province, country) | Role or Contribution, eg, chair, principal investigator | Group (if more than 1 Group listed in the byline) and/or Subgroup (eg, Steering Committee) |
|---|---|---|---|---|---|---|---|
| Dara M. | Cucinotta | | RN | University of Pennsylvania, Hospital of the University of Pennsylvania, Pennsylvania Hospital, Children's Hospital of Philadelphia, and Virtua Voorhees Hospital | Philadelphia, PA and Voorhees, NJ | Non-Author Contributor | Eunice Kennedy Shriver National Institute of Child Health and Human Development Neonatal Research Network |
| Judy C. | Bernbaum | | MD | University of Pennsylvania, Hospital of the University of Pennsylvania, Pennsylvania Hospital, Children's Hospital of Philadelphia, and Virtua Voorhees Hospital | Philadelphia, PA and Voorhees, NJ | Non-Author Contributor | Eunice Kennedy Shriver National Institute of Child Health and Human Development Neonatal Research Network |
| Marsha | Gerdes | | PhD | University of Pennsylvania, Hospital of the University of Pennsylvania, Pennsylvania Hospital, Children's Hospital of Philadelphia, and Virtua Voorhees Hospital | Philadelphia, PA and Voorhees, NJ | Non-Author Contributor | Eunice Kennedy Shriver National Institute of Child Health and Human Development Neonatal Research Network |
| Sarvin | Ghayam | | MD | University of Pennsylvania, Hospital of the University of Pennsylvania, Pennsylvania Hospital, Children's Hospital of Philadelphia, and Virtua Voorhees Hospital | Philadelphia, PA and Voorhees, NJ | Non-Author Contributor | Eunice Kennedy Shriver National Institute of Child Health and Human Development Neonatal Research Network |
| Hallam | Hurt | | MD | University of Pennsylvania, Hospital of the University of Pennsylvania, Pennsylvania Hospital, Children's Hospital of Philadelphia, and Virtua Voorhees Hospital | Philadelphia, PA and Voorhees, NJ | Non-Author Contributor | Eunice Kennedy Shriver National Institute of Child Health and Human Development Neonatal Research Network |
| Jonathan | Snyder | | RN BSN | University of Pennsylvania, Hospital of the University of Pennsylvania, Pennsylvania Hospital, Children's Hospital of Philadelphia, and Virtua Voorhees Hospital | Philadelphia, PA and Voorhees, NJ | Non-Author Contributor | Eunice Kennedy Shriver National Institute of Child Health and Human Development Neonatal Research Network |
| Kristina | Ziolkowski | | CMA (AAMA) CCRP | University of Pennsylvania, Hospital of the University of Pennsylvania, Pennsylvania Hospital, Children's Hospital of Philadelphia, and Virtua Voorhees Hospital | Philadelphia, PA and Voorhees, NJ | Non-Author Contributor | Eunice Kennedy Shriver National Institute of Child Health and Human Development Neonatal Research Network |
| Carl T. | D'Angio | | MD | University of Rochester Medical Center, Golisano Children's Hospital, and The University of Buffalo Women's and Children's Hospital of Buffalo | Rochester and Buffalo, NY | Non-Author Contributor | Eunice Kennedy Shriver National Institute of Child Health and Human Development Neonatal Research Network |
| Ronnie | Guillet | | MD PhD | University of Rochester Medical Center, Golisano Children's Hospital, and The University of Buffalo Women's and Children's Hospital of Buffalo | Rochester and Buffalo, NY | Non-Author Contributor | Eunice Kennedy Shriver National Institute of Child Health and Human Development Neonatal Research Network |
| Gary J. | Myers | | MD | University of Rochester Medical Center, Golisano Children's Hospital, and The University of Buffalo Women's and Children's Hospital of Buffalo | Rochester and Buffalo, NY | Non-Author Contributor | Eunice Kennedy Shriver National Institute of Child Health and Human Development Neonatal Research Network |
| Anne Marie | Reynolds | | MD | University of Rochester Medical Center, Golisano Children's Hospital, and The University of Buffalo Women's and Children's Hospital of Buffalo | Rochester and Buffalo, NY | Non-Author Contributor | Eunice Kennedy Shriver National Institute of Child Health and Human Development Neonatal Research Network |
| Satyan | Lakshminrusimha | | MD | University of Rochester Medical Center, Golisano Children's Hospital, and The University of Buffalo Women's and Children's Hospital of Buffalo | Rochester and Buffalo, NY | Non-Author Contributor | Eunice Kennedy Shriver National Institute of Child Health and Human Development Neonatal Research Network |
| Holly I.M. | Wadkins | | | University of Rochester Medical Center, Golisano Children's Hospital, and The University of Buffalo Women's and Children's Hospital of Buffalo | Rochester and Buffalo, NY | Non-Author Contributor | Eunice Kennedy Shriver National Institute of Child Health and Human Development Neonatal Research Network |
| Michael G. | Sacilowski | | BS | University of Rochester Medical Center, Golisano Children's Hospital, and The University of Buffalo Women's and Children's Hospital of Buffalo | Rochester and Buffalo, NY | Non-Author Contributor | Eunice Kennedy Shriver National Institute of Child Health and Human Development Neonatal Research Network |
| Rosemary L. | Jensen | | | University of Rochester Medical Center, Golisano Children's Hospital, and The University of Buffalo Women's and Children's Hospital of Buffalo | Rochester and Buffalo, NY | Non-Author Contributor | Eunice Kennedy Shriver National Institute of Child Health and Human Development Neonatal Research Network |

| *First Name and Middle Initial(s) | *Last Name | *Suffix (eg, Jr, III) | Academic Degrees | Institution | Location (city, state/province, country) | Role or Contribution, eg, chair, principal investigator | Group (if more than 1 Group listed in the byline) and/or Subgroup (eg, Steering Committee) |
|---|---|---|---|---|---|---|---|
| Joan | Merzbach | | LMSW | University of Rochester Medical Center, Golisano Children's Hospital, and The University of Buffalo Women's and Children's Hospital of Buffalo | Rochester and Buffalo, NY | Non-Author Contributor | Eunice Kennedy Shriver National Institute of Child Health and Human Development Neonatal Research Network |
| William | Zorn | | PhD | University of Rochester Medical Center, Golisano Children's Hospital, and The University of Buffalo Women's and Children's Hospital of Buffalo | Rochester and Buffalo, NY | Non-Author Contributor | Eunice Kennedy Shriver National Institute of Child Health and Human Development Neonatal Research Network |
| Osman | Farooq | | MD | University of Rochester Medical Center, Golisano Children's Hospital, and The University of Buffalo Women's and Children's Hospital of Buffalo | Rochester and Buffalo, NY | Non-Author Contributor | Eunice Kennedy Shriver National Institute of Child Health and Human Development Neonatal Research Network |
| Dee | Maffett | | RN | University of Rochester Medical Center, Golisano Children's Hospital, and The University of Buffalo Women's and Children's Hospital of Buffalo | Rochester and Buffalo, NY | Non-Author Contributor | Eunice Kennedy Shriver National Institute of Child Health and Human Development Neonatal Research Network |
| Ashley | Williams | | MSEd | University of Rochester Medical Center, Golisano Children's Hospital, and The University of Buffalo Women's and Children's Hospital of Buffalo | Rochester and Buffalo, NY | Non-Author Contributor | Eunice Kennedy Shriver National Institute of Child Health and Human Development Neonatal Research Network |
| Julianne | Hunn | | BS | University of Rochester Medical Center, Golisano Children's Hospital, and The University of Buffalo Women's and Children's Hospital of Buffalo | Rochester and Buffalo, NY | Non-Author Contributor | Eunice Kennedy Shriver National Institute of Child Health and Human Development Neonatal Research Network |
| Stephanie | Guilford | | BS | University of Rochester Medical Center, Golisano Children's Hospital, and The University of Buffalo Women's and Children's Hospital of Buffalo | Rochester and Buffalo, NY | Non-Author Contributor | Eunice Kennedy Shriver National Institute of Child Health and Human Development Neonatal Research Network |
| Kelley | Yost | | PhD | University of Rochester Medical Center, Golisano Children's Hospital, and The University of Buffalo Women's and Children's Hospital of Buffalo | Rochester and Buffalo, NY | Non-Author Contributor | Eunice Kennedy Shriver National Institute of Child Health and Human Development Neonatal Research Network |
| Mary | Rowan | | RN | University of Rochester Medical Center, Golisano Children's Hospital, and The University of Buffalo Women's and Children's Hospital of Buffalo | Rochester and Buffalo, NY | Non-Author Contributor | Eunice Kennedy Shriver National Institute of Child Health and Human Development Neonatal Research Network |
| Diane | Prinzing | | | University of Rochester Medical Center, Golisano Children's Hospital, and The University of Buffalo Women's and Children's Hospital of Buffalo | Rochester and Buffalo, NY | Non-Author Contributor | Eunice Kennedy Shriver National Institute of Child Health and Human Development Neonatal Research Network |
| Karen | Wynn | | RN | University of Rochester Medical Center, Golisano Children's Hospital, and The University of Buffalo Women's and Children's Hospital of Buffalo | Rochester and Buffalo, NY | Non-Author Contributor | Eunice Kennedy Shriver National Institute of Child Health and Human Development Neonatal Research Network |
| Melissa | Bowman | | RN NP | University of Rochester Medical Center, Golisano Children's Hospital, and The University of Buffalo Women's and Children's Hospital of Buffalo | Rochester and Buffalo, NY | Non-Author Contributor | Eunice Kennedy Shriver National Institute of Child Health and Human Development Neonatal Research Network |
| Linda J. | Reubens | | RN CCRC | University of Rochester Medical Center, Golisano Children's Hospital, and The University of Buffalo Women's and Children's Hospital of Buffalo | Rochester and Buffalo, NY | Non-Author Contributor | Eunice Kennedy Shriver National Institute of Child Health and Human Development Neonatal Research Network |
| Ann Marie | Scorsone | | MS CCRC | University of Rochester Medical Center, Golisano Children's Hospital, and The University of Buffalo Women's and Children's Hospital of Buffalo | Rochester and Buffalo, NY | Non-Author Contributor | Eunice Kennedy Shriver National Institute of Child Health and Human Development Neonatal Research Network |
| Michelle | Hartley-McAndrew | | MD | University of Rochester Medical Center, Golisano Children's Hospital, and The University of Buffalo Women's and Children's Hospital of Buffalo | Rochester and Buffalo, NY | Non-Author Contributor | Eunice Kennedy Shriver National Institute of Child Health and Human Development Neonatal Research Network |

| *First Name and Middle Initial(s) | *Last Name | *Suffix (eg, Jr, III) | Academic Degrees | Institution | Location (city, state/province, country) | Role or Contribution, eg, chair, principal investigator | Group (if more than 1 Group listed in the byline) and/or Subgroup (eg, Steering Committee) |
|---|---|---|---|---|---|---|---|
| Caitlin | Fallone | | MA | University of Rochester Medical Center, Golisano Children's Hospital, and The University of Buffalo Women's and Children's Hospital of Buffalo | Rochester and Buffalo, NY | Non-Author Contributor | Eunice Kennedy Shriver National Institute of Child Health and Human Development Neonatal Research Network |
| Kyle | Binion | | BS | University of Rochester Medical Center, Golisano Children's Hospital, and The University of Buffalo Women's and Children's Hospital of Buffalo | Rochester and Buffalo, NY | Non-Author Contributor | Eunice Kennedy Shriver National Institute of Child Health and Human Development Neonatal Research Network |
| Constance | Orme | | | University of Rochester Medical Center, Golisano Children's Hospital, and The University of Buffalo Women's and Children's Hospital of Buffalo | Rochester and Buffalo, NY | Non-Author Contributor | Eunice Kennedy Shriver National Institute of Child Health and Human Development Neonatal Research Network |
| Premini | Sabaratnam | | MPH | University of Rochester Medical Center, Golisano Children's Hospital, and The University of Buffalo Women's and Children's Hospital of Buffalo | Rochester and Buffalo, NY | Non-Author Contributor | Eunice Kennedy Shriver National Institute of Child Health and Human Development Neonatal Research Network |
| Alison | Kent | | BMBS FRACP MD | University of Rochester Medical Center, Golisano Children's Hospital, and The University of Buffalo Women's and Children's Hospital of Buffalo | Rochester and Buffalo, NY | Non-Author Contributor | Eunice Kennedy Shriver National Institute of Child Health and Human Development Neonatal Research Network |
| Cassandra A. | Horihan | | MS | University of Rochester Medical Center, Golisano Children's Hospital, and The University of Buffalo Women's and Children's Hospital of Buffalo | Rochester and Buffalo, NY | Non-Author Contributor | Eunice Kennedy Shriver National Institute of Child Health and Human Development Neonatal Research Network |
| Rachel | Jones | | | University of Rochester Medical Center, Golisano Children's Hospital, and The University of Buffalo Women's and Children's Hospital of Buffalo | Rochester and Buffalo, NY | Non-Author Contributor | Eunice Kennedy Shriver National Institute of Child Health and Human Development Neonatal Research Network |
| Elizabeth | Boylin | | BA | University of Rochester Medical Center, Golisano Children's Hospital, and The University of Buffalo Women's and Children's Hospital of Buffalo | Rochester and Buffalo, NY | Non-Author Contributor | Eunice Kennedy Shriver National Institute of Child Health and Human Development Neonatal Research Network |
| Daisy | Rochez | | BS MHA | University of Rochester Medical Center, Golisano Children's Hospital, and The University of Buffalo Women's and Children's Hospital of Buffalo | Rochester and Buffalo, NY | Non-Author Contributor | Eunice Kennedy Shriver National Institute of Child Health and Human Development Neonatal Research Network |
| Emily | Li | | BA | University of Rochester Medical Center, Golisano Children's Hospital, and The University of Buffalo Women's and Children's Hospital of Buffalo | Rochester and Buffalo, NY | Non-Author Contributor | Eunice Kennedy Shriver National Institute of Child Health and Human Development Neonatal Research Network |
| Jennifer | Kachelmeyer | | BS | University of Rochester Medical Center, Golisano Children's Hospital, and The University of Buffalo Women's and Children's Hospital of Buffalo | Rochester and Buffalo, NY | Non-Author Contributor | Eunice Kennedy Shriver National Institute of Child Health and Human Development Neonatal Research Network |
| Kimberly G. | McKee | | BS | University of Rochester Medical Center, Golisano Children's Hospital, and The University of Buffalo Women's and Children's Hospital of Buffalo | Rochester and Buffalo, NY | Non-Author Contributor | Eunice Kennedy Shriver National Institute of Child Health and Human Development Neonatal Research Network |
| Kelly R. | Coleman | | PsyD | University of Rochester Medical Center, Golisano Children's Hospital, and The University of Buffalo Women's and Children's Hospital of Buffalo | Rochester and Buffalo, NY | Non-Author Contributor | Eunice Kennedy Shriver National Institute of Child Health and Human Development Neonatal Research Network |
| Myra H. | Wyckoff | | MD | University of Texas Southwestern Medical Center, Parkland Health & Hospital System, and Children's Medical Center Dallas | Dallas, TX | Non-Author Contributor | Eunice Kennedy Shriver National Institute of Child Health and Human Development Neonatal Research Network |
| Luc P. | Brion | | MD | University of Texas Southwestern Medical Center, Parkland Health & Hospital System, and Children's Medical Center Dallas | Dallas, TX | Non-Author Contributor | Eunice Kennedy Shriver National Institute of Child Health and Human Development Neonatal Research Network |

| *First Name and Middle Initial(s) | *Last Name | *Suffix (eg, Jr, III) | Academic Degrees | Institution | Location (city, state/province, country) | Role or Contribution, eg, chair, principal investigator | Group (if more than 1 Group listed in the byline) and/or Subgroup (eg, Steering Committee) |
|---|---|---|---|---|---|---|---|
| Roy J. | Heyne | | MD | University of Texas Southwestern Medical Center, Parkland Health & Hospital System, and Children's Medical Center Dallas | Dallas, TX | Non-Author Contributor | Eunice Kennedy Shriver National Institute of Child Health and Human Development Neonatal Research Network |
| Diana M. | Vasil | | MSN BSN RNC-NIC | University of Texas Southwestern Medical Center, Parkland Health & Hospital System, and Children's Medical Center Dallas | Dallas, TX | Non-Author Contributor | Eunice Kennedy Shriver National Institute of Child Health and Human Development Neonatal Research Network |
| Sally S. | Adams | | MS RN CPNP | University of Texas Southwestern Medical Center, Parkland Health & Hospital System, and Children's Medical Center Dallas | Dallas, TX | Non-Author Contributor | Eunice Kennedy Shriver National Institute of Child Health and Human Development Neonatal Research Network |
| Lijun | Chen | | RN PHD | University of Texas Southwestern Medical Center, Parkland Health & Hospital System, and Children's Medical Center Dallas | Dallas, TX | Non-Author Contributor | Eunice Kennedy Shriver National Institute of Child Health and Human Development Neonatal Research Network |
| Maria M. | De Leon | | RN BSN | University of Texas Southwestern Medical Center, Parkland Health & Hospital System, and Children's Medical Center Dallas | Dallas, TX | Non-Author Contributor | Eunice Kennedy Shriver National Institute of Child Health and Human Development Neonatal Research Network |
| Frances | Eubanks | | RN BSN | University of Texas Southwestern Medical Center, Parkland Health & Hospital System, and Children's Medical Center Dallas | Dallas, TX | Non-Author Contributor | Eunice Kennedy Shriver National Institute of Child Health and Human Development Neonatal Research Network |
| Rebecca | McDougald | | MSN APRN CPNP-PC/AC | University of Texas Southwestern Medical Center, Parkland Health & Hospital System, and Children's Medical Center Dallas | Dallas, TX | Non-Author Contributor | Eunice Kennedy Shriver National Institute of Child Health and Human Development Neonatal Research Network |
| Lara | Payageau | | MD | University of Texas Southwestern Medical Center, Parkland Health & Hospital System, and Children's Medical Center Dallas | Dallas, TX | Non-Author Contributor | Eunice Kennedy Shriver National Institute of Child Health and Human Development Neonatal Research Network |
| Pollieanna | Sepulveda | | RN | University of Texas Southwestern Medical Center, Parkland Health & Hospital System, and Children's Medical Center Dallas | Dallas, TX | Non-Author Contributor | Eunice Kennedy Shriver National Institute of Child Health and Human Development Neonatal Research Network |
| Alicia | Guzman | | | University of Texas Southwestern Medical Center, Parkland Health & Hospital System, and Children's Medical Center Dallas | Dallas, TX | Non-Author Contributor | Eunice Kennedy Shriver National Institute of Child Health and Human Development Neonatal Research Network |
| Elizabeth | Heyne | | PsyD PA-C | University of Texas Southwestern Medical Center, Parkland Health & Hospital System, and Children's Medical Center Dallas | Dallas, TX | Non-Author Contributor | Eunice Kennedy Shriver National Institute of Child Health and Human Development Neonatal Research Network |
| Linda A. | Madden | | RN BSN CPNP | University of Texas Southwestern Medical Center, Parkland Health & Hospital System, and Children's Medical Center Dallas | Dallas, TX | Non-Author Contributor | Eunice Kennedy Shriver National Institute of Child Health and Human Development Neonatal Research Network |
| Lizette E. | Lee | | RN | University of Texas Southwestern Medical Center, Parkland Health & Hospital System, and Children's Medical Center Dallas | Dallas, TX | Non-Author Contributor | Eunice Kennedy Shriver National Institute of Child Health and Human Development Neonatal Research Network |
| Cathy | Twell Boatman | | MS CIMI | University of Texas Southwestern Medical Center, Parkland Health & Hospital System, and Children's Medical Center Dallas | Dallas, TX | Non-Author Contributor | Eunice Kennedy Shriver National Institute of Child Health and Human Development Neonatal Research Network |
| Azucena | Vera | | AS | University of Texas Southwestern Medical Center, Parkland Health & Hospital System, and Children's Medical Center Dallas | Dallas, TX | Non-Author Contributor | Eunice Kennedy Shriver National Institute of Child Health and Human Development Neonatal Research Network |

| *First Name and Middle Initial(s) | *Last Name | *Suffix (eg, Jr, III) | Academic Degrees | Institution | Location (city, state/province, country) | Role or Contribution, eg, chair, principal investigator | Group (if more than 1 Group listed in the byline) and/or Subgroup (eg, Steering Committee) |
|---|---|---|---|---|---|---|---|
| Jillian | Waterbury | | DNP RN CPNP-PC | University of Texas Southwestern Medical Center, Parkland Health & Hospital System, and Children's Medical Center Dallas | Dallas, TX | Non-Author Contributor | Eunice Kennedy Shriver National Institute of Child Health and Human Development Neonatal Research Network |
| Bradley A. | Yoder | | MD | University of Utah Medical Center, Intermountain Medical Center, McKay-Dee Hospital, Utah Valley Hospital, and Primary Children's Medical Center | Murray, Ogden, Provo, and Salt Lake City, UT | Non-Author Contributor | Eunice Kennedy Shriver National Institute of Child Health and Human Development Neonatal Research Network |
| Mariana | Baserga | | MD MSCI | University of Utah Medical Center, Intermountain Medical Center, McKay-Dee Hospital, Utah Valley Hospital, and Primary Children's Medical Center | Murray, Ogden, Provo, and Salt Lake City, UT | Non-Author Contributor | Eunice Kennedy Shriver National Institute of Child Health and Human Development Neonatal Research Network |
| Roger G. | Faix | | MD | University of Utah Medical Center, Intermountain Medical Center, McKay-Dee Hospital, Utah Valley Hospital, and Primary Children's Medical Center | Murray, Ogden, Provo, and Salt Lake City, UT | Non-Author Contributor | Eunice Kennedy Shriver National Institute of Child Health and Human Development Neonatal Research Network |
| Stephen D. | Minton | | MD | University of Utah Medical Center, Intermountain Medical Center, McKay-Dee Hospital, Utah Valley Hospital, and Primary Children's Medical Center | Murray, Ogden, Provo, and Salt Lake City, UT | Non-Author Contributor | Eunice Kennedy Shriver National Institute of Child Health and Human Development Neonatal Research Network |
| Mark J. | Sheffield | | MD | University of Utah Medical Center, Intermountain Medical Center, McKay-Dee Hospital, Utah Valley Hospital, and Primary Children's Medical Center | Murray, Ogden, Provo, and Salt Lake City, UT | Non-Author Contributor | Eunice Kennedy Shriver National Institute of Child Health and Human Development Neonatal Research Network |
| Carrie A. | Rau | | RN BSN CCRC | University of Utah Medical Center, Intermountain Medical Center, McKay-Dee Hospital, Utah Valley Hospital, and Primary Children's Medical Center | Murray, Ogden, Provo, and Salt Lake City, UT | Non-Author Contributor | Eunice Kennedy Shriver National Institute of Child Health and Human Development Neonatal Research Network |
| Shawna | Baker | | RN | University of Utah Medical Center, Intermountain Medical Center, McKay-Dee Hospital, Utah Valley Hospital, and Primary Children's Medical Center | Murray, Ogden, Provo, and Salt Lake City, UT | Non-Author Contributor | Eunice Kennedy Shriver National Institute of Child Health and Human Development Neonatal Research Network |
| Jill | Burnett | | RNC BSN | University of Utah Medical Center, Intermountain Medical Center, McKay-Dee Hospital, Utah Valley Hospital, and Primary Children's Medical Center | Murray, Ogden, Provo, and Salt Lake City, UT | Non-Author Contributor | Eunice Kennedy Shriver National Institute of Child Health and Human Development Neonatal Research Network |
| Susan | Christensen | | RN | University of Utah Medical Center, Intermountain Medical Center, McKay-Dee Hospital, Utah Valley Hospital, and Primary Children's Medical Center | Murray, Ogden, Provo, and Salt Lake City, UT | Non-Author Contributor | Eunice Kennedy Shriver National Institute of Child Health and Human Development Neonatal Research Network |
| Sean D. | Cunningham | | PhD | University of Utah Medical Center, Intermountain Medical Center, McKay-Dee Hospital, Utah Valley Hospital, and Primary Children's Medical Center | Murray, Ogden, Provo, and Salt Lake City, UT | Non-Author Contributor | Eunice Kennedy Shriver National Institute of Child Health and Human Development Neonatal Research Network |
| Brandy | Davis | | RN BSN | University of Utah Medical Center, Intermountain Medical Center, McKay-Dee Hospital, Utah Valley Hospital, and Primary Children's Medical Center | Murray, Ogden, Provo, and Salt Lake City, UT | Non-Author Contributor | Eunice Kennedy Shriver National Institute of Child Health and Human Development Neonatal Research Network |
| Jennifer O. | Elmont | | RN BSN | University of Utah Medical Center, Intermountain Medical Center, McKay-Dee Hospital, Utah Valley Hospital, and Primary Children's Medical Center | Murray, Ogden, Provo, and Salt Lake City, UT | Non-Author Contributor | Eunice Kennedy Shriver National Institute of Child Health and Human Development Neonatal Research Network |
| Becky | Hall | | APRN | University of Utah Medical Center, Intermountain Medical Center, McKay-Dee Hospital, Utah Valley Hospital, and Primary Children's Medical Center | Murray, Ogden, Provo, and Salt Lake City, UT | Non-Author Contributor | Eunice Kennedy Shriver National Institute of Child Health and Human Development Neonatal Research Network |
| Erika R. | Jensen | | APRN | University of Utah Medical Center, Intermountain Medical Center, McKay-Dee Hospital, Utah Valley Hospital, and Primary Children's Medical Center | Murray, Ogden, Provo, and Salt Lake City, UT | Non-Author Contributor | Eunice Kennedy Shriver National Institute of Child Health and Human Development Neonatal Research Network |

*Indicates required information. Only first name, last name, and suffix will appear in PubMed.

| ***First Name and Middle Initial(s)** | ***Last Name** | ***Suffix (eg, Jr, III)** | Academic Degrees | Institution | Location (city, state/province, country) | Role or Contribution, eg, chair, principal investigator | Group (if more than 1 Group listed in the byline) and/or Subgroup (eg, Steering Committee) |
|---|---|---|---|---|---|---|---|
| Manndi C. | Loertscher | | BS CCRP | University of Utah Medical Center, Intermountain Medical Center, McKay-Dee Hospital, Utah Valley Hospital, and Primary Children's Medical Center | Murray, Ogden, Provo, and Salt Lake City, UT | Non-Author Contributor | Eunice Kennedy Shriver National Institute of Child Health and Human Development Neonatal Research Network |
| Trisha | Marchant | | RNC BSN | University of Utah Medical Center, Intermountain Medical Center, McKay-Dee Hospital, Utah Valley Hospital, and Primary Children's Medical Center | Murray, Ogden, Provo, and Salt Lake City, UT | Non-Author Contributor | Eunice Kennedy Shriver National Institute of Child Health and Human Development Neonatal Research Network |
| Earl | Maxson | | RN CCRN | University of Utah Medical Center, Intermountain Medical Center, McKay-Dee Hospital, Utah Valley Hospital, and Primary Children's Medical Center | Murray, Ogden, Provo, and Salt Lake City, UT | Non-Author Contributor | Eunice Kennedy Shriver National Institute of Child Health and Human Development Neonatal Research Network |
| Kandace M. | McGrath | | BS | University of Utah Medical Center, Intermountain Medical Center, McKay-Dee Hospital, Utah Valley Hospital, and Primary Children's Medical Center | Murray, Ogden, Provo, and Salt Lake City, UT | Non-Author Contributor | Eunice Kennedy Shriver National Institute of Child Health and Human Development Neonatal Research Network |
| Hena G. | Mickelsen | | BA | University of Utah Medical Center, Intermountain Medical Center, McKay-Dee Hospital, Utah Valley Hospital, and Primary Children's Medical Center | Murray, Ogden, Provo, and Salt Lake City, UT | Non-Author Contributor | Eunice Kennedy Shriver National Institute of Child Health and Human Development Neonatal Research Network |
| Galina | Morshedzadeh | | BSN APRN | University of Utah Medical Center, Intermountain Medical Center, McKay-Dee Hospital, Utah Valley Hospital, and Primary Children's Medical Center | Murray, Ogden, Provo, and Salt Lake City, UT | Non-Author Contributor | Eunice Kennedy Shriver National Institute of Child Health and Human Development Neonatal Research Network |
| Melody D. | Parry | | RN BSN | University of Utah Medical Center, Intermountain Medical Center, McKay-Dee Hospital, Utah Valley Hospital, and Primary Children's Medical Center | Murray, Ogden, Provo, and Salt Lake City, UT | Non-Author Contributor | Eunice Kennedy Shriver National Institute of Child Health and Human Development Neonatal Research Network |
| Susan T. | Schaefer | | RN BSN RRT | University of Utah Medical Center, Intermountain Medical Center, McKay-Dee Hospital, Utah Valley Hospital, and Primary Children's Medical Center | Murray, Ogden, Provo, and Salt Lake City, UT | Non-Author Contributor | Eunice Kennedy Shriver National Institute of Child Health and Human Development Neonatal Research Network |
| Kelly | Stout | | PhD | University of Utah Medical Center, Intermountain Medical Center, McKay-Dee Hospital, Utah Valley Hospital, and Primary Children's Medical Center | Murray, Ogden, Provo, and Salt Lake City, UT | Non-Author Contributor | Eunice Kennedy Shriver National Institute of Child Health and Human Development Neonatal Research Network |
| Ashley L. | Stuart | | PhD | University of Utah Medical Center, Intermountain Medical Center, McKay-Dee Hospital, Utah Valley Hospital, and Primary Children's Medical Center | Murray, Ogden, Provo, and Salt Lake City, UT | Non-Author Contributor | Eunice Kennedy Shriver National Institute of Child Health and Human Development Neonatal Research Network |
| Kimberlee | Weaver-Lewis | | RN MS | University of Utah Medical Center, Intermountain Medical Center, McKay-Dee Hospital, Utah Valley Hospital, and Primary Children's Medical Center | Murray, Ogden, Provo, and Salt Lake City, UT | Non-Author Contributor | Eunice Kennedy Shriver National Institute of Child Health and Human Development Neonatal Research Network |
| Sarah | Winter | | MD | University of Utah Medical Center, Intermountain Medical Center, McKay-Dee Hospital, Utah Valley Hospital, and Primary Children's Medical Center | Murray, Ogden, Provo, and Salt Lake City, UT | Non-Author Contributor | Eunice Kennedy Shriver National Institute of Child Health and Human Development Neonatal Research Network |
| Kathryn D. | Woodbury | | RN BSN | University of Utah Medical Center, Intermountain Medical Center, McKay-Dee Hospital, Utah Valley Hospital, and Primary Children's Medical Center | Murray, Ogden, Provo, and Salt Lake City, UT | Non-Author Contributor | Eunice Kennedy Shriver National Institute of Child Health and Human Development Neonatal Research Network |
| Seetha | Shankaran | | MD | Wayne State University, Hutzel Women's Hospital, and Children's Hospital of Michigan | Detroit, MI | Non-Author Contributor | Eunice Kennedy Shriver National Institute of Child Health and Human Development Neonatal Research Network |
| Girija | Natarajan | | MD | Wayne State University, Hutzel Women's Hospital, and Children's Hospital of Michigan | Detroit, MI | Non-Author Contributor | Eunice Kennedy Shriver National Institute of Child Health and Human Development Neonatal Research Network |

*Indicates required information. Only first name, last name, and suffix will appear in PubMed.

| ***First Name and Middle Initial(s)** | ***Last Name** | ***Suffix (eg, Jr, III)** | Academic Degrees | Institution | Location (city, state/province, country) | Role or Contribution, eg, chair, principal investigator | Group (if more than 1 Group listed in the byline) and/or Subgroup (eg, Steering Committee) |
|---|---|---|---|---|---|---|---|
| Athina | Pappas | | MD | Wayne State University, Hutzel Women's Hospital, and Children's Hospital of Michigan | Detroit, MI | Non-Author Contributor | Eunice Kennedy Shriver National Institute of Child Health and Human Development Neonatal Research Network |
| Beena G. | Sood | | MD MS | Wayne State University, Hutzel Women's Hospital, and Children's Hospital of Michigan | Detroit, MI | Non-Author Contributor | Eunice Kennedy Shriver National Institute of Child Health and Human Development Neonatal Research Network |
| Monika | Bajaj | | MD | Wayne State University, Hutzel Women's Hospital, and Children's Hospital of Michigan | Detroit, MI | Non-Author Contributor | Eunice Kennedy Shriver National Institute of Child Health and Human Development Neonatal Research Network |
| Melissa | February | | MD | Wayne State University, Hutzel Women's Hospital, and Children's Hospital of Michigan | Detroit, MI | Non-Author Contributor | Eunice Kennedy Shriver National Institute of Child Health and Human Development Neonatal Research Network |
| Prashant | Agarwal | | MD | Wayne State University, Hutzel Women's Hospital, and Children's Hospital of Michigan | Detroit, MI | Non-Author Contributor | Eunice Kennedy Shriver National Institute of Child Health and Human Development Neonatal Research Network |
| Sanjay | Chawla | | MD | Wayne State University, Hutzel Women's Hospital, and Children's Hospital of Michigan | Detroit, MI | Non-Author Contributor | Eunice Kennedy Shriver National Institute of Child Health and Human Development Neonatal Research Network |
| Rebecca | Bara | | RN BSN | Wayne State University, Hutzel Women's Hospital, and Children's Hospital of Michigan | Detroit, MI | Non-Author Contributor | Eunice Kennedy Shriver National Institute of Child Health and Human Development Neonatal Research Network |
| Kirsten | Childs | | RN BSN | Wayne State University, Hutzel Women's Hospital, and Children's Hospital of Michigan | Detroit, MI | Non-Author Contributor | Eunice Kennedy Shriver National Institute of Child Health and Human Development Neonatal Research Network |
| Eunice | Woldt | | RN MSN | Wayne State University, Hutzel Women's Hospital, and Children's Hospital of Michigan | Detroit, MI | Non-Author Contributor | Eunice Kennedy Shriver National Institute of Child Health and Human Development Neonatal Research Network |
| Laura | Goldston | | MA | Wayne State University, Hutzel Women's Hospital, and Children's Hospital of Michigan | Detroit, MI | Non-Author Contributor | Eunice Kennedy Shriver National Institute of Child Health and Human Development Neonatal Research Network |
| John | Barks | | MD | Wayne State University, Hutzel Women's Hospital, and Children's Hospital of Michigan | Detroit, MI | Non-Author Contributor | Eunice Kennedy Shriver National Institute of Child Health and Human Development Neonatal Research Network |
| Stephanie A. | Wiggins | | MS | Wayne State University, Hutzel Women's Hospital, and Children's Hospital of Michigan | Detroit, MI | Non-Author Contributor | Eunice Kennedy Shriver National Institute of Child Health and Human Development Neonatal Research Network |
| Mary K. | Christensen | | BA RRT | Wayne State University, Hutzel Women's Hospital, and Children's Hospital of Michigan | Detroit, MI | Non-Author Contributor | Eunice Kennedy Shriver National Institute of Child Health and Human Development Neonatal Research Network |
| Martha | Carlson | | MD | Wayne State University, Hutzel Women's Hospital, and Children's Hospital of Michigan | Detroit, MI | Non-Author Contributor | Eunice Kennedy Shriver National Institute of Child Health and Human Development Neonatal Research Network |
| Diane F. | White | | RRT CCRP | Wayne State University, Hutzel Women's Hospital, and Children's Hospital of Michigan | Detroit, MI | Non-Author Contributor | Eunice Kennedy Shriver National Institute of Child Health and Human Development Neonatal Research Network |
