# Supplementary material for: Assessment of Corticosteroid Therapy and Death or Disability According to Pretreatment Risk of Death or Bronchopulmonary Dysplasia in Extremely Preterm Infants
Source: JAMA Netw Open. 2023 May 8;6(5):e2312277. doi: 10.1001/jamanetworkopen.2023.12277 (PMC10167571; doi:10.1001/jamanetworkopen.2023.12277)
Supplement: Supplement 3. — Data Sharing Statement [file jamanetwopen-e2312277-s003.pdf]

Data Sharing Statement

Jensen. Assessment of Corticosteroid Therapy and Death or Disability According to Pretreatment Risk of Death or Bronchopulmonary Dysplasia in Extremely Preterm Infants. *JAMA Netw Open*. Published May 08, 2023. doi:10.1001/jamanetworkopen.2023.12277

Data
Data available: Yes
Data types: Deidentified participant data
How to access data: RTI.org
When available: With publication

Supporting Documents
Document types: None

Additional Information
Who can access the data: researchers whose proposed use of the data has been approved by the NICHD neonatal research network.
Types of analyses: Use will be considered based on NRN approval.
Mechanisms of data availability: Details available at RTI.org
